# Expression of long noncoding RNAs in peripheral blood mononuclear cells of patients with type 1 diabetes mellitus: potential biomarkers for disease onset

**DOI:** 10.20945/2359-4292-2024-0496

**Published:** 2025-10-08

**Authors:** Cristine Dieter, Natália Emerim Lemos, Eliandra Girardi, Eloisa Toscan Massignam, Thayne Woycinck Kowalski, Mariana Recamonde-Mendoza, Márcia Puñales, Taís Silveira Assmann, Daisy Crispim

**Affiliations:** 1 Serviço de Endocrinologia, Hospital de Clínicas de Porto Alegre, Porto Alegre, RS, Brasil; 2 Programa de Pós-graduação em Ciências Médicas: Endocrinologia, Faculdade de Medicina, Departamento de Medicina Interna, Universidade Federal do Rio Grande do Sul, Porto Alegre, RS, Brasil; 3 Programa de Pós-graduação em Saúde e Desenvolvimento Humano, Universidade La Salle, Canoas, RS, Brasil; 4 Departamento de Bioquímica, Instituto de Química, Universidade de São Paulo, São Paulo, SP, Brasil; 5 Microbial Ecology and Genomics Laboratory, Istituto Zooprofilattico Sperimentale dele Venezie, Legnaro, Italy; 6 National PhD Program in One Health Approaches to Infectious Diseases and Life Science Research, Department of Public Health, Experimental and Forensic Medicine, University of Pavia, Pavia, Italy; 7 Núcleo de Bioinformática, Hospital de Clínicas de Porto Alegre, Porto Alegre, RS, Brasil; 8 Instituto de Informática, Universidade Federal do Rio Grande do Sul, Porto Alegre, RS, Brasil; 9 Instituto da Criança com Diabetes, Hospital Nossa Senhora da Conceição, Porto Alegre, RS, Brasil

**Keywords:** Type 1 diabetes mellitus, LncRNAs, MALAT1, MEG3, TUG1

## Abstract

**Objective:**

Long non-coding RNAs (lncRNAs) do not encode proteins and are transcripts longer than 200 nucleotides. The precise involvement of lncRNAs in type 1 diabetes mellitus (T1DM) pathogenesis remains unclear. Therefore, this study aimed to analyze the expressions of five lncRNAs in peripheral blood mononuclear cells of individuals with T1DM and without DM.

**Materials and methods:**

This study comprised 27 patients with T1DM (cases) and 13 individuals without DM (controls). The case group was divided into two subgroups based on T1DM duration: < 5 years of diagnosis group and long-term diabetes group (≥5 years). LncRNA expression was evaluated by qPCR.

**Results:**

*MALAT1* and *TUG1* were upregulated in patients within the first five years of diagnosis of T1DM compared to the other groups. *MEG3* was upregulated in the case group of < 5 years of diagnosis compared to controls. *TUG1* and *MALAT1* levels were negatively correlated with the duration of T1DM, while *TUG1* and *MEG3* were positively correlated with glycated hemoglobin levels. Bioinformatics analysis revealed that *MALAT1, MEG3*, and *TUG1* regulate and interact with protein-codifying genes and microRNAs involved in T1DM-related pathways.

**Conclusion:**

Our study revealed MALAT1, MEG3, and TUG1 upregulation in patients within the first five years of diagnosis of T1DM.

## INTRODUCTION

Type 1 diabetes mellitus (T1DM) is a metabolic disease caused by the autoimmune destruction of pancreatic beta-cells, which leads to exogenous insulin dependence in patients with this disease (^1,2^). T1DM accounts for 10%-15% of all diabetes cases and can occur in people at any age, although it typically develops in children and young adults (^1^). The autoimmunity against beta-cells is triggered by a complex interaction between genetic, epigenetic, and environmental factors (^1,2^).

Epigenetic factors are heritable modifications in gene expression that do not change the nucleotide sequence of DNA (^3^). The main epigenetic mechanisms are DNA methylation, posttranslational modifications of histones, and gene expression regulation by non-coding RNAs (ncRNAs) (^4^). NcRNAs can regulate gene expression by various mechanisms, such as repressing or activating transcription, modifying chromatin structure, and post-transcriptional regulation (^5,6^). NncRNAs can generally be classified into short (<200 nucleotides) and long (>200 nucleotides) types based on their length (^7^). Long non-coding RNAs (lncRNAs) can participate in numerous gene regulatory activities, such as transcription, splicing, protein degradation, and chromatin modifications, thereby modifying chromatin states and influencing gene expression (^8,9^). They also have a key role in regulating the expression of microRNAs (miRNAs), which are short ncRNAs that regulate gene expression (^10^).

Several studies have reported that numerous lncRNAs contribute to inflammation, apoptosis, insulin secretion, and autoimmune dysfunction in both immune cells and beta-cells (^11^-^14^). Yin and cols. (^15^) revealed that silencing the lncRNA *TUG1* led to higher beta-cell apoptotic rates, resulting in reduced insulin secretion. Dysregulations in lncRNAs have also been described in pancreatic cells and murine models of diabetes mellitus (DM). The lncRNA *Malat1* was upregulated in serum from insulin-resistant C57BL/6J mice compared to control mice (^16^). Knockdown of the lncRNA *Pvt1* ameliorated streptozotocin-induced oxidative stress and apoptosis and elevated the insulin secretory capacity of beta-cells (^17^). Moreover, Dieter and cols. (^18^) conducted a systematic review that highlighted six lncRNAs, including *MIAT, MALAT1*, and *MEG3*, that were dysregulated in patients with DM (mainly type 2 DM) compared to controls in a number of studies. This systematic review also pointed out the lack of studies focused on lncRNA expressions in individuals with T1DM.

Therefore, we conducted a case-control study to analyze the expressions of six lncRNAs, namely *MIAT, MALAT1, MEG3, TUG1*, and *PVT1* in peripheral blood mononuclear cells (PBMCs) from individuals with and without T1DM. Additionally, we performed bioinformatics analyses to explore the potential targets and biological pathways regulated by the lncRNAs of interest.

## MATERIALS AND METHODS

### Study population

This study was designed following the STROBE guidelines for performing and reporting observational studies (^19^). The sample comprised 27 patients with T1DM [14 of them had < 5 years of diagnosis and 13 had ≥ 5 years of diagnosis (long-term diabetes group)] and 13 individuals without DM (controls). All T1DM patients were recruited from *Hospital de Clínicas de Porto Alegre* (HCPA) and *Instituto da Criança com Diabetes* (ICD) - Grupo Hospitalar Conceição (Rio Grande do Sul, Brazil) between November 2019 and May 2022. T1DM diagnosis followed the American Diabetes Association recommendations (^20^). The exclusion criteria were: a febrile episode within the last months, chronic inflammatory or rheumatic diseases, hepatitis, any active infection, HIV, hereditary dyslipidemia, errors of metabolism (except for DM), or glucocorticoid treatment.

The control group comprised blood donors recruited from the HCPA between November 2019 and May 2022. Only individuals with glycated hemoglobin (HbA1c) ≤ 5.7% were included in this group (^20^). Moreover, individuals who had any active infection, or a family history of diabetes were not included in the control group.

We collected clinical information using a standard questionnaire for the T1DM group. As previously reported, all patients underwent comprehensive physical and laboratory evaluations (^21^). For the control group, we collected data on age, ethnicity, family history of DM or other diseases, and occurrence of other exclusion criteria. Weight and height were measured to calculate body mass index (BMI) and blood samples were collected to measure HbA1c levels. Both case and control subjects self-classified their ethnic group.

The study was approved by the Ethic Committees in Research from HCPA and ICD - Grupo Hospital Conceição, and all subjects signed the written informed consent before their participation in the study (CAAE number: 97779118.4.0000.5327).

### RNA extraction and quantification of lncRNA expressions by RT-qPCR

Samples of 4 mL of peripheral blood were collected from individuals with and without T1DM and then 2 mL of blood was mixed with an equal volume of phosphate-buffered saline (Sigma, Missouri, EUA). Total PBMCs were isolated from blood by density gradient centrifugation using the Ficoll-paqueTM plus (GE HealthCare, Uppsala, Sweden) (^22^) and were stored at -80 °C until RNA extraction.

Total RNA was isolated from PBMCs using the PureLink RNA Mini Kit (Thermo Fisher Scientific, Waltham, MA, USA). The NanoDrop ND-1000 Spectrophotometer (Thermo Fisher Scientific) was used to analyze purity and concentration of RNA samples. Only the samples with acceptable purity ratios (A260/A280 = 1.9-2.1) were selected for the following analyses (^23^).

Reverse-transcription real-time quantitative PCR (RT-qPCR) was performed using a two-step protocol. In the first step, the total RNA was reverse transcribed into cDNA using the SuperScript VILO Master Mix IV (Thermo Fisher Scientific) according to the instruction of the manufacturer. In the second step, the cDNA was amplified by qPCR in a ViiA^TM^ 7 Fast Real-Time PCR System (Thermo Fisher Scientific). The qPCR reactions included 0.5 µL of TaqMan Gene Expression Assay (20X) (Thermo Fisher Scientific) for *MALAT1* (Hs00273907_s1), *MIAT* (Hs03300285_g1), *MEG3* (Hs00292028_m1), *PVT1* (Hs00413039_m1), *TUG1* (Hs05579214_s1) or *GAPDH* (Hs02786624_g1), 5 µL of TaqMan Fast Advanced Master Mix (Thermo Fisher Scientific), 1 µL of cDNA (200 ng/µL for *MEG3*, 100 ng/µL for *MIAT, PVT1*, and *TUG1* and 25 ng/µL for *MALAT1*), and sterile water to complete a volume of 10 µL. Each sample was analyzed in triplicate and a negative control was added to each plate. The cycling conditions were as follows: 50 °C for 2 min, 95 °C for 10 min, and 45 cycles of 95 °C for 1s and 60 °C for 20s. The quantification of the lncRNAs of interest was conducted using the 2^-∆∆Cq^ method with the *GAPDH* as the reference gene, and the results are shown as n-folds in relation to the calibrator sample, which was a pool of all cDNA samples analyzed (^23^).

### Bioinformatics analyses

Potential genes targeted by the dysregulated lncRNAs were retrieved from the Encori database (^24^). No restrictions were applied to the type of target, although only protein coding genes identified by two or more studies were included in the subsequent analyses. We also investigated miRNAs regulated by the differently expressed lncRNAs between groups. These data were obtained from the Encori database (^24^) and analyzed via Venn Diagrams. For the common miRNAs regulated by the differently expressed lncRNAs, their target genes were searched via MultimiR package in R (^25^), which is a collection of miRNA/target genes from external resources, including validated and predicted miRNA-target databases (^25^). Although we explored the predicted target genes of miRNAs, we included only targets validated in at least two miRNA databases in our analyses.

Functional overrepresentation analysis of biological processes of the retrieved target genes was performed using KEGG pathways and Gene Ontology (GO) databases, which were incorporated in the clusterProfiler package in the R environment (^26,27^). GO analysis is a commonly used approach for identifying the biological attributes of genes, gene products, and sequences (^28^), while KEGG is a collection of databases that provides information on genomes, biological pathways, diseases, and chemical substances (^29^). GO and KEGG pathways were considered significant if adjusted P-values < 0.05 (q-*values*), with adjustment performed using the Benjamini-Hochberg method.

### Statistical analyses

Kolmogorov Smirnov and Shapiro-Wilk tests were used to evaluate the distribution of variables. Those variables with a normal distribution are reported as mean ± SD, while variables with a skewed distribution were log-transformed before the analyses and are reported as median (25-75th percentiles). Categorical variables are shown as %. Clinical and laboratory variables, as well as lncRNA expressions, were compared between groups using appropriate tests such as One-way ANOVA, Student’s *t,* or χ^2^ tests. Pearson’s correlation test was applied to analyze correlations between quantitative variables. The statistical analyses were conducted using the SPSS statistical package (v.18.0) for Windows (SPSS Inc, Chicago, IL), and P-values < 0.05 were significant.

The adequate sample size was estimated using the OpenEpi site (www.openepi.com), considering a power of 80% (α = 0.05) to detect two-fold (±1.5 SD) differences in lncRNA expressions between groups, as based in previous studies (^30^-^32^). Therefore, at least nine patients in each group were required to achieve sufficient statistical power.

## RESULTS

### Sample description

**Table 1** describes the clinical and laboratory characteristics of patients with T1DM and individuals without DM. Mean age was higher in control and long-term diabetes groups than in patients with < 5 years of T1DM (P < 0.0001). HbA1c was lower in controls than in patients with T1DM (P < 0.0001). There was no difference between groups in gender and ethnicity frequencies (P > 0.050).

**Table 1:** Sample description

Characteristic	Individuals without DM (n = 13)	T1DM < 5 years (n = 14)	T1DM ≥ 5 years (n = 13)	P
Age (years)	41.2 ± 13.8a	21.0 ± 4.3b	34.6 ± 10.8a	<0.0001
Gender (% male)	38.5	57.1	30.8	0.358
Ethnicity (% black)	15.4	7.1	7.7	0.732
BMI (kg/m^2^)	27.1 ± 4.9	23.6 ± 2.3	26.1 ± 4.2	0.074
HbA1c (%)	5.2 ± 0.3a	9.6 ± 2.4b	8.9 ± 1.5b	<0.0001
Hypertension (%)	-	0.0	15.4	0.078
Age at T1DM diagnosis (years)	-	18.2 ± 4.1	15.3 ± 9.0	0.288
Duration of T1DM (years)	-	3.0 ± 1.2	19.3 ± 9.3	<0.0001

Variables are presented as mean ± SD, median (25^th^-75^th^ percentiles), or %. Significant differences between values are denoted by distinct superscript letters (P < 0.05), while statistically similar values are denoted by the same superscript letters. BMI: body mass index; HbA1c: glycated hemoglobin; T1DM: type 1 diabetes mellitus.

### Expression of lncRNAs in PBMCs from patients with T1DM and non-diabetic subjects

**Figure 1:**
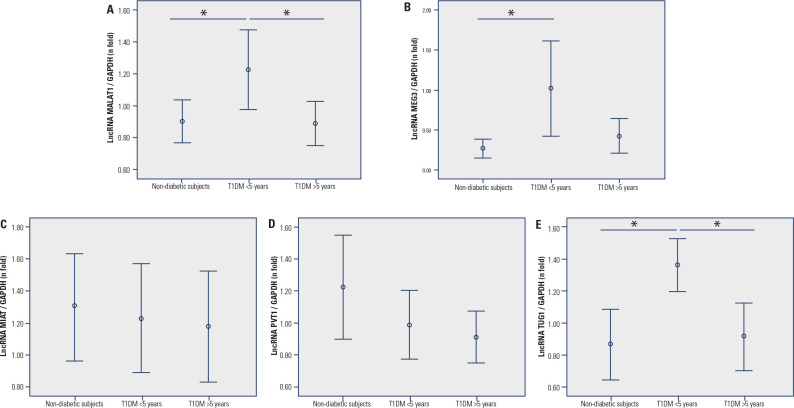
Expressions of lncRNAs *MALAT1* (**A**), *MEG3* (**B**), *MIAT* (**C**), *PVT1* (**D**), and *TUG1* (**E**) in PBMCs of patients with T1DM (with < 5 years or ≥ 5 years of diagnosis) and individuals without T1DM. Relative expressions were quantified using qPCR experiments. Data are shown as fold changes relative to the calibrator, determined using the ∆∆Cq method, and are presented as median (25-75th percentiles). P-values were calculated using one-way ANOVA with LSD post hoc tests. *P < 0.05.

Expressions of the five lncRNAs (*MEG3, MALAT1, MIAT, TUG1*, and *PVT1*) were evaluated in PBMC samples from individuals with T1DM, divided into patients with < 5 years of diagnosis and long-term diabetes group, as well as non-diabetic subjects. **Figure 1** (A and E) shows that *MALAT1* and *TUG1* levels were higher in patients with < 5 years of diagnosis of T1DM compared to the control and long-term diabetes groups [*MALAT1:* 1.23 (0.90-1.410) *vs.* 0.89 (0.81-1.06) *vs.* 0.92 (0.68-1.04), P = 0.008; *TUG1:* 1.42 (1.07-1.61) *vs.* 0.83 (0.56-1.11) *vs.* 0.84 (0.72-1.06), P = 0.001, respectively). *MEG3* expression was higher in < 5 years of diagnosis group compared to the control group [0.62 (0.17-1.54) *vs.* 0.23 (0.170-0.360), P = 0.048] but not with long-term diabetes group (P = 0.121) (**Figure 1B**). Moreover, the expression of this lncRNA did not differ between controls and patients with T1DM with ≥ 5 years of diagnosis (long-term diabetes group) (P = 0.677; **Figure 1B**). *MIAT* and *PVT1* expressions did not differ between the three groups (P = 0.862 and P = 0.281, **Figure 1C and 1D)**.

Then, we evaluated correlations among the expressions of the lncRNAs and HbA1c levels and duration of T1DM. Expressions of *TUG1* and *MALAT1* were negatively correlated with the duration of T1DM [(r = -0.460, P = 0.042) and (r = -0.695, P = 0.0001); respectively]. Moreover, *TUG1* was positively correlated with HbA1c levels (r = 0.471, P = 0.005), and *MEG3* showed a trend towards a positive correlation with HbA1c levels (r = 0.324 and P = 0.081).

### Bioinformatics analysis

**Figure 2:**
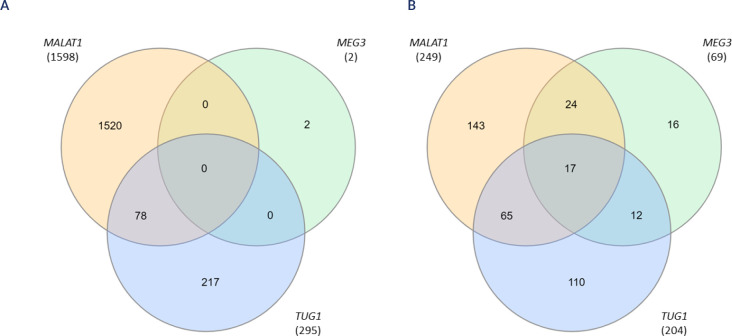
Venn diagram showing the shared target genes (**A**) and miRNAs (**B**) of the three lncRNAs dysregulated in diabetes.

Bioinformatics analyses were conducted to identify potential targets and pathways that may be affected by the three significantly dysregulated lncRNAs in PBMCs of patients with < 5 years of T1DM diagnosis. *MALAT1, MEG3,* and *TUG1* together regulate the expression of 1,817 target genes (**Table S1**). Specifically, *MALAT1* targets 1,598 genes, while *MEG3* targets two genes, and *TUG1* targets 295 genes (**Table S1A and Figure 2A**).

**Figure 3:**
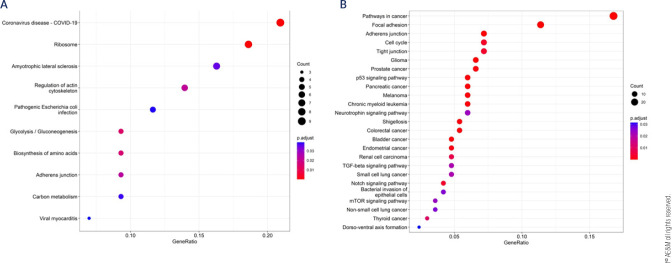
Significant KEGG pathways regulated by the dysregulated lncRNAs in diabetes (**A**), and by the target miRNAs of the three lncRNAs consistently dysregulated in diabetes (**B**).

To gain a better understanding of the biological pathways altered by the dysregulation of these three lncRNAs, we performed a functional enrichment analysis of their protein-coding target genes identified by two or more studies using pathway maps from the KEGG and GO repositories. Since neither of the two targets of *MEG3* was identified by two or more studies, the functional enrichment analysis was performed using only *MALAT1* and *TUG1* protein-coding targets (**Table S1**). Ten unique KEGG pathways were enriched for the lncRNA targets, including glycolysis/gluconeogenesis-, ribosome-, adherens junction-, biosynthesis of amino acids, and regulation of actin cytoskeletonsignaling pathway (**Table S2 and Figure 3A**). Moreover, the protein-coding target genes were involved in 138 GO biological processes (**Table S2**), including those altered in DM pathogenesis, such as canonical glycolysis, glucose catabolic process to pyruvate, glycolic process via glucose-6-phosphate, glucose catabolic process, pyruvate metabolic process, and glycolytic process.

**Figure 4:**
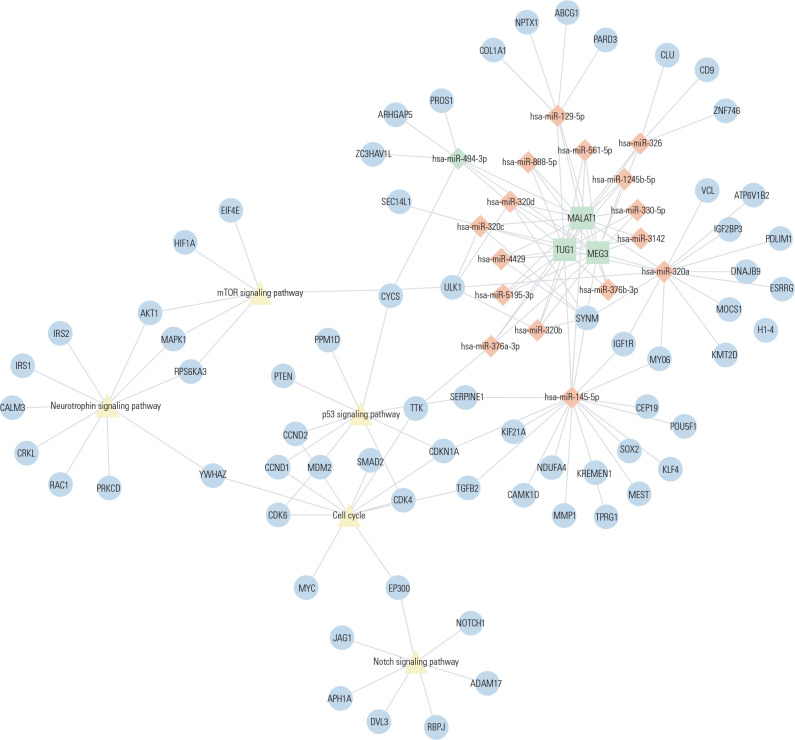
Interactions between lncRNAs, miRNAs, and mRNA-pathways. The lncRNAs are presented as squares, the target miRNAs as diamonds, the target genes of the miRNAs as circles, and the pathways as triangles.

Besides targeting protein-coding genes, lncRNAs also interact and regulate miRNAs. We found that *MALAT1, MEG3*, and *TUG1* regulate 387 unique miRNAs (**Table S3**). *MALAT1* targets 249 miRNAs, *MEG3* targets 69 miRNAs, and *TUG1* targets 204 miRNAs (**Figure 2B**). Of the 387 unique miRNAs, 17 were regulated by the three lncRNAs dysregulated in patients with T1DM and recent diagnosis of this disease (**Figure 4**). These 17 miRNAs have 409 target genes validated in at least two databases (**Table S4**), which are involved in pathways related to DM pathogenesis, including mTOR-, neurotrophin-, Notch-, p53-, and cell cycle-signaling pathways (**Table S5 and Figure 3B**). Moreover, their target genes participated in 997 GO biological process, including regulation of insulin secretion, regulation of apoptotic signaling pathway, response to insulin, response to glucose, and regulation of Notch signaling pathway.

## DISCUSSION

T1DM is an autoimmune disorder triggered by the interaction between genetic, environmental, and epigenetics factors (^3,33^). In the context of epigenetics, some studies have shown the key contribution of lncRNAs in the maintenance of beta-cell mass and function [reviewed in ^34^]. Thus, to better understand the role of lncRNAs in T1DM, we investigated the expression of five candidate lncRNAs in PBMCs from patients with T1DM and individuals without DM, and we found that *MALAT1, MEG3*, and *TUG1* were upregulated in patients with < 5 years of T1DM diagnosis.

*MALAT1* is one of the most extensively studied lncRNAs in human diseases and is widely described to be involved in cancer development (^35^). This lncRNA plays a role in transcriptional and post-transcriptional regulation, and alternative splicing, and is involved in many physiological and pathological processes (^35^). In the context of diabetes, *MALAT1* has been linked to beta-cell dysfunction via inhibition of *pancreatic and duodenal homeobox 1* (*PDX-1)* expression, which leads to reduced H3 histone acetylation (^36^). Moreover, higher levels of *MALAT1* have been observed in human umbilical vein endothelial cells incubated with high glucose concentrations (^37^). This increase in *MALAT1* levels was associated with upregulation of serum amyloid antigen 3, an inflammatory ligand and target of *MALAT1,* as well as with an increase in other inflammatory mediators, including tumor necrosis factor and interleukin-6 cytokines, both of which playing key roles in beta-cell dysfunction (^37^).

We have demonstrated the upregulation of *MALAT1* in PBMCs of patients with < 5 years of diagnosis compared to individuals without DM and long-term diabetes group. In agreement with our results, Santos and cols. (^38^) described higher levels of *MALAT1* in patients with T1DM diagnosed within ≤ 6 months compared to healthy individuals. Sathishkumar and cols. (^39^) also demonstrated the upregulation of *MALAT1* in PBMCs from patients with type 2 DM (T2DM) compared to individuals without DM. Moreover, a systematic review described the upregulation of *MALAT1* in different samples of patients with T2DM compared to controls (^18^).

Our bioinformatics analysis revealed that *MALAT1* has 969 target genes, and is involved in 10 pathways, including glycolysis/gluconeogenesis-, regulation of actin cytoskeleton-, and biosynthesis of amino acidssignaling pathway. Additionally, this lncRNA interacts with 246 miRNAs, including miR-146a-5p and miR-155-5p, which were previously described as dysregulated in patients with recent diagnosis of T1DM (^40^), as well as miR-21-5p, miR150-5p, and miR181c-5p, which were reported as being consistently associated with T1DM in a systematic review published by our group (^41^).

*MEG3* is another important lncRNA that has been extensively studied and reported to be associated with many human diseases, including metabolic, immune system, cardiovascular, and cerebrovascular diseases (^42^). We found an upregulation of *MEG3* in < 5 years of T1DM diagnosis group, which is consistent with the study conducted by Sathishkumar and cols. (^39^) on PBMCs from patients with T2DM. However, other studies have described a downregulation of this lncRNA, mainly in patients with T2DM [reviewed in 18]. Moreover, *MEG3* appears be involved in beta-cell function (^43^). In Balb/c mouse islets, *Meg3* expression was abundant compared to exocrine glands. However, *Meg3* expression was decreased in islets from T1DM (non-obese female mice) and T2DM (db/db mice) models (^43^). The authors also reported that Meg3 is a new regulator of the synthesis and secretion of insulin since *Meg3* suppression influenced insulin production by decreasing the expression of key transcription factors, including *Pdx-1* and *MafA* (^43^).

In addition, this lncRNA binds to different miRNAs to regulate different mechanisms, such as apoptosis, inflammation, oxidative stress, and endoplasmic reticulum stress (^42^). Our bioinformatics analyses showed that *MEG3* interacts with 69 miRNAs, included miR-181c-5p, which was previously reported as dysregulated in patients with T1DM [reviewed in 41].

*TUG1* has been associated with several biological and physiological processes, such as regulation of cell cycle, cell proliferation, migration, death, and gene expression regulation (^44^). In the context of DM, *TUG1* expression has been related to beta-cell apoptosis and insulin secretion in both *in vivo* and *in vitro* experimental models (^15^). Some studies have also revealed the association of this lncRNA with the development of diabetic chronic complications (^45^-^47^). We found an upregulation of *TUG1* in PBMCs from patients with < 5 years of diagnosis compared to long-term diabetes group and controls. Accordingly, Su and cols. (^48^) reported higher levels of *TUG1* in the peripheral blood of patients with T2DM compared to healthy controls. Moreover, the lncRNA *TUG1* had a high accuracy for discriminating patients with T2DM from healthy individuals together with hsa_circ_0071106 and hsa-miR-607 (^48^). Our bioinformatics analysis showed that *TUG1* has 295 direct target genes, which are involved in 10 KEGG pathways and several GO biological processes. Furthermore, *TUG1* interacts with 204 miRNAs, including miR-21-5p, miR-148a-3p, miR-144-3p, and miR-26b-5p, which were previously reported as being dysregulated in patients with T1DM (^41^), including those with a recent diagnosis (^40^).

Moreover, the interaction between lncRNAs and miRNAs changes the expression of target miRNAs, consequently affecting the genes and pathways in which these miRNAs are involved. Thus, our bioinformatics analysis revealed that *MALAT1, MEG3,* and *TUG1* collectively regulate 17 miRNAs. These miRNAs have 409 target genes, which are involved in several pathways associated with DM pathogenesis, including mTOR-, neurotrophin-, Notch-, p53-, and cell cycle-signaling pathways. Notch has been associated with the regulation of beta-cell function and proliferation, playing a key role in beta-cell mass determination and DM (^49,50^). Physiological activation of mTORC1 is also important for the regulation of beta-cell homeostasis, adaptation, development, and insulin secretion, while chronic dysregulation of mTORC1 can lead to beta-cell failure [reviewed in ^51^].

Although our results are important to better understand the involvement of lncRNAs in pathogenesis of DM, we should consider a few limitations when interpreting our findings. Firstly, the small sample size may limit our ability to detect small differences in lncRNA expressions between groups, especially for *MIAT* and *PVT1*. Thus, type II errors may have occurred during comparisons of lncRNA expressions between the study groups. However, it is worth noting that our sample size was calculated to have sufficient statistical power to detect 2-fold differences in lncRNA expressions between the groups, reducing the bias likelihood. Secondly, it is important to acknowledge that a number of variables can influence lncRNA expressions, so we implemented a comprehensive list of exclusion criteria for our patients to minimize the impact of these variables. Thirdly, our study establishes associations rather than causal relationships. Therefore, further experimental studies are necessary to confirm the mechanisms underlying the associations of *MALAT1, MEG3*, and *TUG1* with the initial phases of T1DM. Despite these limitations, this study holds significance as one of the initial reports on lncRNAs expression in individuals with T1DM, and the first in a Brazilian population.

In conclusion, our study revealed the upregulation of the lncRNAs *MALAT1, MEG3*, and *TUG1* in patients within the first five years of diagnosis of T1DM compared to controls and long-term diabetes group. The findings suggest that these lncRNAs may be potential biomarkers for the onset of T1DM. Furthermore, our results indicate that these three lncRNAs target multiple genes and miRNAs involved in pathways associated with DM pathogenesis. However, further studies are necessary to elucidate the precise mechanisms via which *MALAT1, MEG3*, and *TUG1* directly impact T1DM pathogenesis.

## Data Availability

datasets related to this article will be available upon request to the corresponding author.

## LIST OF SUPPLEMENTARY MATERIAL

**Table S1:** Target genes of the lncRNAs *MALAT1, MEG3*, and *TUG1*

LncRNA	Target	Gene Type	Number of experiments
MALAT1	PLXND1	protein_coding	1
MALAT1	AK2	protein_coding	1
MALAT1	GDE1	protein_coding	1
MALAT1	TMEM132A	protein_coding	1
MALAT1	MGST1	protein_coding	1
MALAT1	ZNF207	protein_coding	1
MALAT1	AKAP8L	protein_coding	1
MALAT1	PTBP1	protein_coding	1
MALAT1	SYT7	protein_coding	1
MALAT1	ANLN	protein_coding	1
MALAT1	WIZ	protein_coding	1
MALAT1	ERCC1	protein_coding	1
MALAT1	TACC3	protein_coding	2
MALAT1	CAPN1	protein_coding	1
MALAT1	MDH1	protein_coding	1
MALAT1	STRAP	protein_coding	1
MALAT1	NCAPH2	protein_coding	1
MALAT1	ADSS	protein_coding	1
MALAT1	RIPOR1	protein_coding	1
MALAT1	PSMA4	protein_coding	1
MALAT1	CTNNA1	protein_coding	1
MALAT1	GUCA1A	protein_coding	1
MALAT1	LIMA1	protein_coding	1
MALAT1	TTC17	protein_coding	1
MALAT1	AKR7A2	protein_coding	1
MALAT1	AP5M1	protein_coding	1
MALAT1	PPP1R12A	protein_coding	1
MALAT1	CCAR1	protein_coding	1
MALAT1	APPBP2	protein_coding	1
MALAT1	HIPK2	protein_coding	1
MALAT1	BORCS8-MEF2B	protein_coding	1
MALAT1	ADAT1	protein_coding	1
MALAT1	TMEM206	protein_coding	1
MALAT1	ISOC1	protein_coding	1
MALAT1	SP100	protein_coding	2
MALAT1	PKM	protein_coding	3
MALAT1	RHOA	protein_coding	1
MALAT1	DGCR2	protein_coding	3
MALAT1	CSNK2A2	protein_coding	1
MALAT1	SNX13	protein_coding	1
MALAT1	TRIP13	protein_coding	1
MALAT1	PDCD2	protein_coding	1
MALAT1	HMMR	protein_coding	1
MALAT1	CLNS1A	protein_coding	1
MALAT1	ZZEF1	protein_coding	1
MALAT1	ENO1	protein_coding	5
MALAT1	TTC38	protein_coding	1
MALAT1	MARK3	protein_coding	1
MALAT1	ACTB	protein_coding	2
MALAT1	BCAP29	protein_coding	1
MALAT1	FBLN1	protein_coding	1
MALAT1	GNB1	protein_coding	1
MALAT1	SENP1	protein_coding	1
MALAT1	DNM2	protein_coding	1
MALAT1	EPB41L2	protein_coding	1
MALAT1	SMARCA2	protein_coding	1
MALAT1	NFE2L1	protein_coding	1
MALAT1	NOA1	protein_coding	1
MALAT1	MAP3K4	protein_coding	1
MALAT1	IGSF9	protein_coding	1
MALAT1	PGS1	protein_coding	1
MALAT1	GNAS	protein_coding	1
MALAT1	CNOT3	protein_coding	1
MALAT1	KHSRP	protein_coding	1
MALAT1	ANKRD10	protein_coding	1
MALAT1	C20orf194	protein_coding	1
MALAT1	FUS	protein_coding	2
MALAT1	GANAB	protein_coding	2
MALAT1	BIRC5	protein_coding	2
MALAT1	MLF2	protein_coding	1
MALAT1	NECAP1	protein_coding	1
MALAT1	PCBP4	protein_coding	1
MALAT1	TFAP4	protein_coding	1
MALAT1	HNRNPC	protein_coding	1
MALAT1	EZR	protein_coding	1
MALAT1	UPRT	protein_coding	1
MALAT1	ARCN1	protein_coding	1
MALAT1	WAC	protein_coding	1
MALAT1	FKBP5	protein_coding	1
MALAT1	DSP	protein_coding	1
MALAT1	CDC7	protein_coding	1
MALAT1	SCD	protein_coding	1
MALAT1	ERMP1	protein_coding	1
MALAT1	CRKL	protein_coding	1
MALAT1	DDX17	protein_coding	1
MALAT1	GTPBP1	protein_coding	1
MALAT1	RPL3	protein_coding	2
MALAT1	COCH	protein_coding	1
MALAT1	PSMC6	protein_coding	1
MALAT1	VTI1B	protein_coding	1
MALAT1	TCL1A	protein_coding	1
MALAT1	ADNP	protein_coding	1
MALAT1	HM13	protein_coding	2
MALAT1	ACOT8	protein_coding	1
MALAT1	PGK1	protein_coding	2
MALAT1	ARMCX3	protein_coding	1
MALAT1	CCL22	protein_coding	1
MALAT1	COG4	protein_coding	1
MALAT1	NAGPA	protein_coding	1
MALAT1	COTL1	protein_coding	2
MALAT1	ZNF500	protein_coding	1
MALAT1	NUBP1	protein_coding	1
MALAT1	GSPT1	protein_coding	1
MALAT1	DNAJA3	protein_coding	1
MALAT1	SFRP1	protein_coding	1
MALAT1	GPI	protein_coding	1
MALAT1	HNRNPUL1	protein_coding	1
MALAT1	CD33	protein_coding	1
MALAT1	GRWD1	protein_coding	1
MALAT1	ZNF175	protein_coding	1
MALAT1	PPP2R1A	protein_coding	1
MALAT1	RPL18A	protein_coding	1
MALAT1	ISYNA1	protein_coding	1
MALAT1	ZKSCAN1	protein_coding	1
MALAT1	COA1	protein_coding	1
MALAT1	UBE2R2	protein_coding	1
MALAT1	PLEKHA1	protein_coding	1
MALAT1	UNC5B	protein_coding	1
MALAT1	ACTA2	protein_coding	1
MALAT1	ARHGAP21	protein_coding	1
MALAT1	ZMIZ1	protein_coding	1
MALAT1	CASC3	protein_coding	1
MALAT1	CBX1	protein_coding	1
MALAT1	PFN1	protein_coding	1
MALAT1	RNF167	protein_coding	1
MALAT1	DDX5	protein_coding	1
MALAT1	DHX15	protein_coding	1
MALAT1	MTCH2	protein_coding	1
MALAT1	HSPA8	protein_coding	3
MALAT1	LPXN	protein_coding	1
MALAT1	PRPF19	protein_coding	1
MALAT1	GALNT18	protein_coding	1
MALAT1	UBE4A	protein_coding	1
MALAT1	MDK	protein_coding	1
MALAT1	RPS13	protein_coding	3
MALAT1	CORO1C	protein_coding	1
MALAT1	OAS2	protein_coding	3
MALAT1	SLC38A1	protein_coding	1
MALAT1	CPSF6	protein_coding	1
MALAT1	CDCA3	protein_coding	1
MALAT1	TPI1	protein_coding	2
MALAT1	COX6A1	protein_coding	2
MALAT1	SRSF9	protein_coding	1
MALAT1	MAPK14	protein_coding	1
MALAT1	FIG4	protein_coding	1
MALAT1	MRPL2	protein_coding	1
MALAT1	VEGFA	protein_coding	1
MALAT1	SLC29A1	protein_coding	2
MALAT1	PAPD7	protein_coding	1
MALAT1	HMGCR	protein_coding	1
MALAT1	CCNG1	protein_coding	1
MALAT1	SLC12A7	protein_coding	1
MALAT1	PPP2CA	protein_coding	1
MALAT1	DBN1	protein_coding	2
MALAT1	HEMK1	protein_coding	1
MALAT1	NEK4	protein_coding	1
MALAT1	DGUOK	protein_coding	1
MALAT1	MOB1A	protein_coding	1
MALAT1	TTL	protein_coding	1
MALAT1	ZNF142	protein_coding	1
MALAT1	CNPPD1	protein_coding	2
MALAT1	NFE2L2	protein_coding	1
MALAT1	RALGPS2	protein_coding	1
MALAT1	STXBP3	protein_coding	1
MALAT1	ASH1L	protein_coding	1
MALAT1	ARHGEF2	protein_coding	1
MALAT1	WLS	protein_coding	1
MALAT1	BMP8B	protein_coding	1
MALAT1	SDHB	protein_coding	1
MALAT1	KDM5B	protein_coding	2
MALAT1	AKR1A1	protein_coding	1
MALAT1	IRF6	protein_coding	1
MALAT1	STMN1	protein_coding	1
MALAT1	PPP1R8	protein_coding	1
MALAT1	CTSD	protein_coding	1
MALAT1	SPCS2	protein_coding	1
MALAT1	PLAGL1	protein_coding	1
MALAT1	FBXO30	protein_coding	1
MALAT1	TNFAIP3	protein_coding	1
MALAT1	CCND2	protein_coding	2
MALAT1	TJP2	protein_coding	1
MALAT1	HEATR1	protein_coding	1
MALAT1	ALG2	protein_coding	1
MALAT1	FCF1	protein_coding	1
MALAT1	NEK9	protein_coding	1
MALAT1	TCP1	protein_coding	1
MALAT1	ETF1	protein_coding	1
MALAT1	TMPO	protein_coding	1
MALAT1	FAM117A	protein_coding	1
MALAT1	LRIF1	protein_coding	1
MALAT1	UBL3	protein_coding	1
MALAT1	ANXA11	protein_coding	1
MALAT1	BBS9	protein_coding	1
MALAT1	HNRNPA2B1	protein_coding	2
MALAT1	POLM	protein_coding	1
MALAT1	SERPINB6	protein_coding	1
MALAT1	CDKN1A	protein_coding	1
MALAT1	NUP153	protein_coding	1
MALAT1	SEPT6	protein_coding	1
MALAT1	PPP1R12C	protein_coding	1
MALAT1	CAPNS1	protein_coding	1
MALAT1	COX6B1	protein_coding	1
MALAT1	IFI6	protein_coding	1
MALAT1	PLEKHG3	protein_coding	1
MALAT1	EMC1	protein_coding	1
MALAT1	EPS15L1	protein_coding	1
MALAT1	CHTF18	protein_coding	1
MALAT1	TUBBP1	transcribed_processed_pseudogene	1
MALAT1	TUBA4A	protein_coding	1
MALAT1	CASD1	protein_coding	1
MALAT1	PRKRIP1	protein_coding	1
MALAT1	SNRPN	protein_coding	1
MALAT1	VPS13C	protein_coding	1
MALAT1	COPB1	protein_coding	1
MALAT1	CCNT1	protein_coding	1
MALAT1	ILF3	protein_coding	1
MALAT1	SLC44A2	protein_coding	1
MALAT1	NEDD8	protein_coding	1
MALAT1	ASH2L	protein_coding	1
MALAT1	TOMM40	protein_coding	1
MALAT1	PXDN	protein_coding	1
MALAT1	UBE2M	protein_coding	1
MALAT1	GGT7	protein_coding	1
MALAT1	PPT1	protein_coding	1
MALAT1	VPS25	protein_coding	1
MALAT1	TNS4	protein_coding	1
MALAT1	EFR3A	protein_coding	1
MALAT1	UBE2G1	protein_coding	1
MALAT1	ANKRD17	protein_coding	1
MALAT1	EIF5A	protein_coding	1
MALAT1	FLOT2	protein_coding	1
MALAT1	KHDC4	protein_coding	1
MALAT1	DCTN4	protein_coding	1
MALAT1	SLC41A1	protein_coding	1
MALAT1	TPT1	protein_coding	6
MALAT1	WDR74	protein_coding	8
MALAT1	MBD2	protein_coding	1
MALAT1	BHLHE40	protein_coding	1
MALAT1	LDHA	protein_coding	1
MALAT1	ECHDC3	protein_coding	1
MALAT1	CCNH	protein_coding	1
MALAT1	GNL2	protein_coding	1
MALAT1	SLC43A3	protein_coding	1
MALAT1	TAOK3	protein_coding	1
MALAT1	MDFIC	protein_coding	1
MALAT1	ESPL1	protein_coding	1
MALAT1	KRT7	protein_coding	2
MALAT1	SMYD5	protein_coding	1
MALAT1	KCNK1	protein_coding	1
MALAT1	URB2	protein_coding	1
MALAT1	GLUL	protein_coding	1
MALAT1	MRPL44	protein_coding	1
MALAT1	LRCH1	protein_coding	1
MALAT1	LCP1	protein_coding	1
MALAT1	KAT7	protein_coding	1
MALAT1	SAP130	protein_coding	1
MALAT1	DNAJB5	protein_coding	1
MALAT1	DCTN3	protein_coding	1
MALAT1	TJAP1	protein_coding	1
MALAT1	HMGA1	protein_coding	2
MALAT1	FXYD6	protein_coding	1
MALAT1	RTF1	protein_coding	1
MALAT1	PPIG	protein_coding	1
MALAT1	SEPT11	protein_coding	1
MALAT1	FRAS1	protein_coding	1
MALAT1	GABARAPL1	protein_coding	1
MALAT1	WARS	protein_coding	1
MALAT1	SLC12A6	protein_coding	1
MALAT1	SERF2	protein_coding	1
MALAT1	ETFA	protein_coding	1
MALAT1	ARHGAP17	protein_coding	1
MALAT1	UTP4	protein_coding	1
MALAT1	PCTP	protein_coding	1
MALAT1	G6PC3	protein_coding	1
MALAT1	RMC1	protein_coding	1
MALAT1	HSPG2	protein_coding	1
MALAT1	PTPRF	protein_coding	1
MALAT1	POU2F1	protein_coding	1
MALAT1	SDHC	protein_coding	1
MALAT1	MCL1	protein_coding	1
MALAT1	ANP32E	protein_coding	1
MALAT1	SEMA6C	protein_coding	1
MALAT1	C1orf43	protein_coding	1
MALAT1	RPL32	protein_coding	1
MALAT1	ARL6IP5	protein_coding	1
MALAT1	LIMD1	protein_coding	1
MALAT1	ATG3	protein_coding	1
MALAT1	MUC4	protein_coding	2
MALAT1	KLHL8	protein_coding	1
MALAT1	PHIP	protein_coding	1
MALAT1	DYNLT1	protein_coding	1
MALAT1	NAPRT	protein_coding	1
MALAT1	GTF3C5	protein_coding	1
MALAT1	EIF4EBP2	protein_coding	2
MALAT1	CNNM2	protein_coding	1
MALAT1	PAK1	protein_coding	1
MALAT1	RPS3	protein_coding	3
MALAT1	NCAM1	protein_coding	1
MALAT1	B3GAT3	protein_coding	1
MALAT1	ITGB1	protein_coding	1
MALAT1	PDCD4	protein_coding	1
MALAT1	UBC	protein_coding	1
MALAT1	EXT2	protein_coding	1
MALAT1	NEK7	protein_coding	1
MALAT1	GFRA1	protein_coding	1
MALAT1	BAG3	protein_coding	1
MALAT1	HOMER1	protein_coding	1
MALAT1	IMPACT	protein_coding	1
MALAT1	CC2D1B	protein_coding	1
MALAT1	PPP4R1	protein_coding	1
MALAT1	VOPP1	protein_coding	2
MALAT1	PPARGC1B	protein_coding	1
MALAT1	C8orf37	protein_coding	1
MALAT1	BACH1	protein_coding	1
MALAT1	RPL30	protein_coding	1
MALAT1	TYSND1	protein_coding	1
MALAT1	PRR14	protein_coding	1
MALAT1	RPUSD3	protein_coding	1
MALAT1	RNF111	protein_coding	1
MALAT1	MYO1E	protein_coding	1
MALAT1	TSC22D3	protein_coding	1
MALAT1	TAB3	protein_coding	1
MALAT1	FMNL2	protein_coding	1
MALAT1	BABAM2	protein_coding	1
MALAT1	CNNM4	protein_coding	2
MALAT1	WASF2	protein_coding	1
MALAT1	TAGLN2	protein_coding	1
MALAT1	ELK4	protein_coding	1
MALAT1	DUSP23	protein_coding	1
MALAT1	F11R	protein_coding	1
MALAT1	MPZ	protein_coding	1
MALAT1	EPB41	protein_coding	1
MALAT1	IGF2BP1	protein_coding	2
MALAT1	CHAF1B	protein_coding	1
MALAT1	ADIPOR1	protein_coding	1
MALAT1	BTG2	protein_coding	1
MALAT1	ADGRG5	protein_coding	1
MALAT1	ABR	protein_coding	1
MALAT1	PRMT2	protein_coding	1
MALAT1	SHC1	protein_coding	1
MALAT1	SLC25A44	protein_coding	1
MALAT1	BDH1	protein_coding	1
MALAT1	SRSF2	protein_coding	1
MALAT1	EMC10	protein_coding	1
MALAT1	SNRNP25	protein_coding	1
MALAT1	LAPTM5	protein_coding	1
MALAT1	IGSF8	protein_coding	1
MALAT1	WDR26	protein_coding	1
MALAT1	SGCB	protein_coding	1
MALAT1	LRRC58	protein_coding	1
MALAT1	RPL22L1	protein_coding	1
MALAT1	RPL9	protein_coding	1
MALAT1	U2SURP	protein_coding	1
MALAT1	TKT	protein_coding	1
MALAT1	TMEM184C	protein_coding	1
MALAT1	MIOS	protein_coding	1
MALAT1	YWHAZ	protein_coding	1
MALAT1	INTS8	protein_coding	1
MALAT1	HNRNPK	protein_coding	1
MALAT1	ARHGAP12	protein_coding	1
MALAT1	REEP3	protein_coding	1
MALAT1	ATP5F1C	protein_coding	1
MALAT1	PRPF18	protein_coding	1
MALAT1	PDZD8	protein_coding	1
MALAT1	TAF1D	protein_coding	1
MALAT1	NDUFB8	protein_coding	2
MALAT1	PCBD1	protein_coding	1
MALAT1	ZNF202	protein_coding	1
MALAT1	ANAPC16	protein_coding	1
MALAT1	PRTG	protein_coding	1
MALAT1	B2M	protein_coding	3
MALAT1	PCLAF	protein_coding	1
MALAT1	VPS39	protein_coding	1
MALAT1	MARS	protein_coding	1
MALAT1	FBXO22	protein_coding	1
MALAT1	CDK12	protein_coding	1
MALAT1	RPL13	protein_coding	1
MALAT1	TUBA1C	protein_coding	1
MALAT1	LENG8	protein_coding	1
MALAT1	EEF2	protein_coding	2
MALAT1	CYB5D2	protein_coding	1
MALAT1	C19orf48	protein_coding	1
MALAT1	NDUFV1	protein_coding	1
MALAT1	ZNF232	protein_coding	1
MALAT1	SDHAF2	protein_coding	1
MALAT1	RPSA	protein_coding	3
MALAT1	SEPT2	protein_coding	1
MALAT1	PKIG	protein_coding	1
MALAT1	MAT2A	protein_coding	1
MALAT1	INPP5D	protein_coding	1
MALAT1	SLC25A6	protein_coding	1
MALAT1	MMGT1	protein_coding	1
MALAT1	HINT1	protein_coding	2
MALAT1	HIC2	protein_coding	1
MALAT1	LINGO1	protein_coding	1
MALAT1	ALCAM	protein_coding	1
MALAT1	PWWP2A	protein_coding	1
MALAT1	ZNF282	protein_coding	1
MALAT1	LGALS9B	protein_coding	1
MALAT1	UBB	protein_coding	1
MALAT1	HSPA4	protein_coding	1
MALAT1	GSTA4	protein_coding	1
MALAT1	BPTF	protein_coding	1
MALAT1	ZIK1	protein_coding	1
MALAT1	ANO5	protein_coding	1
MALAT1	BSG	protein_coding	2
MALAT1	HCFC1	protein_coding	1
MALAT1	CORO1B	protein_coding	1
MALAT1	CFL1	protein_coding	4
MALAT1	NOC3L	protein_coding	1
MALAT1	RAPH1	protein_coding	1
MALAT1	GPR137	protein_coding	1
MALAT1	RNF26	protein_coding	1
MALAT1	UQCRH	protein_coding	1
MALAT1	RNF213	protein_coding	1
MALAT1	DPY19L1	protein_coding	1
MALAT1	PRPF8	protein_coding	2
MALAT1	RALGAPA1	protein_coding	1
MALAT1	IGDCC3	protein_coding	1
MALAT1	PTDSS2	protein_coding	1
MALAT1	SMAD2	protein_coding	1
MALAT1	RPL7AP66	processed_pseudogene	1
MALAT1	PLEKHF2	protein_coding	1
MALAT1	LRRN1	protein_coding	1
MALAT1	NFATC2IP	protein_coding	1
MALAT1	TALDO1	protein_coding	1
MALAT1	MAN1B1	protein_coding	1
MALAT1	RPL13AP3	transcribed_processed_pseudogene	1
MALAT1	ZFAS1	antisense	1
MALAT1	RPLP2	protein_coding	2
MALAT1	HNRNPA0	protein_coding	1
MALAT1	ZNF354B	protein_coding	3
MALAT1	EPM2AIP1	protein_coding	1
MALAT1	TMEM107	protein_coding	1
MALAT1	CALR	protein_coding	1
MALAT1	PTPN11	protein_coding	1
MALAT1	MROH1	protein_coding	2
MALAT1	R3HDM2	protein_coding	1
MALAT1	NRXN1	protein_coding	1
MALAT1	ARHGAP45	protein_coding	1
MALAT1	EIF2S3B	protein_coding	1
MALAT1	FRAT2	protein_coding	1
MALAT1	ZNF678	protein_coding	1
MALAT1	C5orf24	protein_coding	1
MALAT1	ADO	protein_coding	1
MALAT1	PNMA8A	protein_coding	1
MALAT1	TMEM259	protein_coding	1
MALAT1	NGRN	protein_coding	1
MALAT1	PYCR1	protein_coding	1
MALAT1	SF3A3	protein_coding	1
MALAT1	TMEM50A	protein_coding	1
MALAT1	TOB2	protein_coding	1
MALAT1	ACTG1	protein_coding	2
MALAT1	BRD7P2	processed_pseudogene	1
MALAT1	NIPSNAP1	protein_coding	2
MALAT1	HDDC3	protein_coding	1
MALAT1	DGAT1	protein_coding	1
MALAT1	RAB11B	protein_coding	1
MALAT1	MRPL40	protein_coding	1
MALAT1	ANKRD46	protein_coding	2
MALAT1	RPS23	protein_coding	2
MALAT1	SELL	protein_coding	1
MALAT1	ACTBP11	processed_pseudogene	1
MALAT1	NDOR1	protein_coding	1
MALAT1	CLN3	protein_coding	1
MALAT1	SUMO2	protein_coding	1
MALAT1	LITAF	protein_coding	3
MALAT1	JPT1	protein_coding	1
MALAT1	RNU1-75P	snRNA	1
MALAT1	EEF1A1P5	processed_pseudogene	2
MALAT1	NIF3L1	protein_coding	1
MALAT1	IARS	protein_coding	1
MALAT1	LONP1	protein_coding	1
MALAT1	TRRAP	protein_coding	2
MALAT1	PTPN1	protein_coding	2
MALAT1	EPHB4	protein_coding	1
MALAT1	XRCC6	protein_coding	1
MALAT1	NACA	protein_coding	1
MALAT1	SULF2	protein_coding	1
MALAT1	TOMM7	protein_coding	1
MALAT1	MVB12B	protein_coding	1
MALAT1	ARHGEF12	protein_coding	2
MALAT1	FLNA	protein_coding	1
MALAT1	NOP9	protein_coding	1
MALAT1	METTL9	protein_coding	1
MALAT1	DDI2	protein_coding	1
MALAT1	SIPA1L1	protein_coding	1
MALAT1	RPS26	protein_coding	1
MALAT1	OCLN	protein_coding	1
MALAT1	PLCG2	protein_coding	1
MALAT1	RPL12	protein_coding	1
MALAT1	MPZL1	protein_coding	1
MALAT1	SLC29A3	protein_coding	1
MALAT1	MGEA5	protein_coding	1
MALAT1	CTNND1	protein_coding	2
MALAT1	MTCO3P12	unprocessed_pseudogene	5
MALAT1	MT-CO1	protein_coding	8
MALAT1	UBE2J1	protein_coding	1
MALAT1	MTND4LP30	processed_pseudogene	5
MALAT1	MT-ND4	protein_coding	3
MALAT1	MT-ND1	protein_coding	1
MALAT1	PRC1	protein_coding	1
MALAT1	ATG9A	protein_coding	1
MALAT1	APRT	protein_coding	1
MALAT1	MT-CO3	protein_coding	2
MALAT1	RNA5S12	rRNA	1
MALAT1	RNA5S11	rRNA	1
MALAT1	RNU5E-1	snRNA	1
MALAT1	RNA5S1	rRNA	1
MALAT1	RNU1-150P	snRNA	2
MALAT1	RNA5SP370	rRNA	1
MALAT1	RNU1-94P	snRNA	3
MALAT1	RF00019	misc_RNA	2
MALAT1	RNU5A-1	snRNA	1
MALAT1	RNU1-14P	snRNA	5
MALAT1	RNU1-35P	snRNA	2
MALAT1	RNU1-72P	snRNA	1
MALAT1	RF00019	misc_RNA	1
MALAT1	RN7SKP131	misc_RNA	1
MALAT1	RNA5S10	rRNA	2
MALAT1	RNU1-18P	snRNA	3
MALAT1	VTRNA1-1	misc_RNA	1
MALAT1	RNU5B-1	snRNA	1
MALAT1	RNA5SP350	rRNA	1
MALAT1	RNU1-21P	snRNA	1
MALAT1	SNORD118	snoRNA	1
MALAT1	RNU1-87P	snRNA	2
MALAT1	RNA5S6	rRNA	1
MALAT1	RN7SKP160	misc_RNA	1
MALAT1	RNU1-124P	snRNA	1
MALAT1	RN7SKP180	misc_RNA	1
MALAT1	RNU4-1	snRNA	3
MALAT1	RNU1-32P	snRNA	2
MALAT1	RNA5SP48	rRNA	1
MALAT1	RNU1-42P	snRNA	3
MALAT1	RN7SKP87	misc_RNA	2
MALAT1	RNU5A-8P	snRNA	1
MALAT1	RNU1-7P	snRNA	1
MALAT1	Y_RNA	misc_RNA	1
MALAT1	RNU6-776P	snRNA	1
MALAT1	RF00019	misc_RNA	1
MALAT1	RNA5S14	rRNA	1
MALAT1	RNA5SP141	rRNA	1
MALAT1	RNU1-141P	snRNA	1
MALAT1	RF00019	misc_RNA	2
MALAT1	RNVU1-6	snRNA	1
MALAT1	RF00019	misc_RNA	1
MALAT1	RNU1-133P	snRNA	1
MALAT1	RF00019	misc_RNA	1
MALAT1	RNA5SP442	rRNA	1
MALAT1	RF00019	misc_RNA	1
MALAT1	RF00019	misc_RNA	1
MALAT1	RF00019	misc_RNA	1
MALAT1	RNA5SP19	rRNA	1
MALAT1	RN7SKP80	misc_RNA	1
MALAT1	RNVU1-11	snRNA	3
MALAT1	RNU6-1175P	snRNA	2
MALAT1	Y_RNA	misc_RNA	1
MALAT1	RNA5SP329	rRNA	1
MALAT1	Y_RNA	misc_RNA	1
MALAT1	RNU1-51P	snRNA	3
MALAT1	SNORD14C	snoRNA	2
MALAT1	RNA5SP77	rRNA	1
MALAT1	RNU1-84P	snRNA	3
MALAT1	RNY3	misc_RNA	1
MALAT1	RNA5SP283	rRNA	1
MALAT1	RNA5S7	rRNA	5
MALAT1	RNU4-2	snRNA	4
MALAT1	HIST2H3A	protein_coding	1
MALAT1	HLA-DMA	protein_coding	1
MALAT1	HLA-DRA	protein_coding	1
MALAT1	BAG6	protein_coding	1
MALAT1	HLA-E	protein_coding	1
MALAT1	HLA-J	transcribed_unprocessed_pseudogene	1
MALAT1	RACK1	protein_coding	1
MALAT1	TMSB4X	protein_coding	3
MALAT1	ITPRIPL2	protein_coding	1
MALAT1	HCP5	sense_overlapping	1
MALAT1	RAB12	protein_coding	1
MALAT1	HLA-A	protein_coding	1
MALAT1	RNVU1-7	snRNA	6
MALAT1	RNU1-28P	snRNA	3
MALAT1	RNU1-39P	snRNA	3
MALAT1	RF00019	misc_RNA	1
MALAT1	RNU1-1	snRNA	5
MALAT1	RNU1-11P	snRNA	2
MALAT1	RF00019	misc_RNA	1
MALAT1	RNU1-138P	snRNA	1
MALAT1	RNU1-74P	snRNA	1
MALAT1	RNU6-48P	snRNA	1
MALAT1	RNU1-136P	snRNA	4
MALAT1	RF00409	snoRNA	1
MALAT1	RNU6-5P	snRNA	1
MALAT1	RNU1-2	snRNA	3
MALAT1	RNU1-106P	snRNA	2
MALAT1	RNU6-16P	snRNA	1
MALAT1	SNORD14D	snoRNA	1
MALAT1	RNU1-67P	snRNA	3
MALAT1	RNU1-44P	snRNA	2
MALAT1	RNU1-148P	snRNA	1
MALAT1	RNU6-393P	snRNA	1
MALAT1	RNVU1-15	snRNA	2
MALAT1	RNU1-89P	snRNA	3
MALAT1	RNU1-69P	snRNA	2
MALAT1	RNVU1-1	snRNA	2
MALAT1	RNVU1-17	snRNA	1
MALAT1	RNU1-4	snRNA	3
MALAT1	RNVU1-7	snRNA	3
MALAT1	RNU6ATAC4P	snRNA	1
MALAT1	SNORA40	snoRNA	1
MALAT1	IGKC	IG_C_gene	2
MALAT1	IGKJ5	IG_J_gene	1
MALAT1	IGHG4	IG_C_gene	2
MALAT1	IGHG1	IG_C_gene	1
MALAT1	RNU1-82P	snRNA	2
MALAT1	RNU1-77P	snRNA	1
MALAT1	RNA5SP86	rRNA	1
MALAT1	RNU1-56P	snRNA	2
MALAT1	NUP62	protein_coding	1
MALAT1	MLLT11	protein_coding	1
MALAT1	NPM1P50	transcribed_processed_pseudogene	1
MALAT1	MXD3	protein_coding	1
MALAT1	RPS3P6	processed_pseudogene	1
MALAT1	PTPRCAP	protein_coding	1
MALAT1	RPS23P2	processed_pseudogene	2
MALAT1	AC026826.1	processed_pseudogene	1
MALAT1	LDHAP5	processed_pseudogene	1
MALAT1	GAPDHP25	processed_pseudogene	1
MALAT1	RPSAP54	processed_pseudogene	3
MALAT1	SLC25A5P3	processed_pseudogene	1
MALAT1	DNASE1	protein_coding	1
MALAT1	EEF1A1P12	processed_pseudogene	1
MALAT1	AC124312.1	protein_coding	1
MALAT1	ALDOAP2	processed_pseudogene	1
MALAT1	TUBAP2	processed_pseudogene	1
MALAT1	ZBED1	protein_coding	1
MALAT1	LINC01588	lincRNA	1
MALAT1	FDPSP5	processed_pseudogene	1
MALAT1	EEF1A1P29	processed_pseudogene	1
MALAT1	RSC1A1	protein_coding	1
MALAT1	GAPDHP72	transcribed_processed_pseudogene	1
MALAT1	AL627402.1	processed_pseudogene	1
MALAT1	AC016739.1	processed_pseudogene	1
MALAT1	RPS18P9	processed_pseudogene	1
MALAT1	RNU6ATAC	snRNA	1
MALAT1	PLXNA4	protein_coding	1
MALAT1	OR2A25	protein_coding	1
MALAT1	SLC12A8	protein_coding	1
MALAT1	UBA52	protein_coding	1
MALAT1	POTEJ	protein_coding	1
MALAT1	RN7SKP111	misc_RNA	1
MALAT1	RNU2-2P	snRNA	8
MALAT1	RNU2-5P	snRNA	1
MALAT1	RNU2-23P	snRNA	1
MALAT1	RNU2-48P	snRNA	1
MALAT1	RNU2-42P	snRNA	1
MALAT1	RNU2-38P	snRNA	1
MALAT1	RNU2-32P	snRNA	1
MALAT1	NUTM2A-AS1	antisense	1
MALAT1	EEF1A1P8	processed_pseudogene	1
MALAT1	EEF1A1P24	processed_pseudogene	1
MALAT1	AL513328.1	processed_pseudogene	1
MALAT1	AC098614.2	processed_pseudogene	1
MALAT1	AL590762.3	processed_pseudogene	1
MALAT1	HSP90AA2P	processed_pseudogene	1
MALAT1	AC017035.1	processed_pseudogene	1
MALAT1	RPL36P16	processed_pseudogene	1
MALAT1	GAPDHP46	processed_pseudogene	1
MALAT1	PLEKHM1	protein_coding	1
MALAT1	RPS12P26	transcribed_processed_pseudogene	1
MALAT1	AC246787.1	processed_pseudogene	1
MALAT1	NUTM2B-AS1	antisense	2
MALAT1	MTND2P28	unprocessed_pseudogene	5
MALAT1	BX890604.2	processed_pseudogene	1
MALAT1	MTND1P23	unprocessed_pseudogene	1
MALAT1	AL031727.1	processed_pseudogene	2
MALAT1	AC108161.1	processed_pseudogene	1
MALAT1	NOTCH2P1	processed_pseudogene	1
MALAT1	SCDP1	processed_pseudogene	1
MALAT1	FTLP3	processed_pseudogene	2
MALAT1	DANCR	processed_transcript	1
MALAT1	AC005912.1	processed_pseudogene	1
MALAT1	TP73-AS1	transcribed_unitary_pseudogene	1
MALAT1	AC107890.1	processed_pseudogene	1
MALAT1	RPS3AP37	processed_pseudogene	1
MALAT1	AL390728.4	transcribed_unprocessed_pseudogene	1
MALAT1	DHX9P1	processed_pseudogene	1
MALAT1	ATF4P3	processed_pseudogene	1
MALAT1	BX284668.2	lincRNA	2
MALAT1	AL359918.1	processed_pseudogene	1
MALAT1	MTCO1P3	processed_pseudogene	1
MALAT1	MTCO2P12	unprocessed_pseudogene	8
MALAT1	MYL8P	processed_pseudogene	1
MALAT1	MTATP8P2	processed_pseudogene	2
MALAT1	RPL4P4	processed_pseudogene	1
MALAT1	AC245047.2	processed_pseudogene	1
MALAT1	XIST	lincRNA	2
MALAT1	ACBD6	protein_coding	1
MALAT1	AC009245.1	processed_pseudogene	1
MALAT1	DYNLL1P7	processed_pseudogene	1
MALAT1	RPS23P8	processed_pseudogene	1
MALAT1	AC241584.1	processed_pseudogene	1
MALAT1	RPL9P8	pseudogene	1
MALAT1	MIR205HG	processed_transcript	2
MALAT1	RPS18	protein_coding	2
MALAT1	MTND4LP1	processed_pseudogene	2
MALAT1	RPL32P28	processed_pseudogene	1
MALAT1	MTND1P32	processed_pseudogene	1
MALAT1	AL390728.5	lincRNA	1
MALAT1	RPL3P4	processed_pseudogene	2
MALAT1	RPS11P5	processed_pseudogene	1
MALAT1	EEF1A1P14	processed_pseudogene	1
MALAT1	AC009362.1	processed_pseudogene	1
MALAT1	HOTAIRM1	antisense	1
MALAT1	EEF1A1P6	processed_pseudogene	2
MALAT1	ADH5P4	processed_pseudogene	1
MALAT1	AP000936.3	processed_pseudogene	3
MALAT1	RPL13AP20	processed_pseudogene	1
MALAT1	JRK	protein_coding	1
MALAT1	YWHAEP5	processed_pseudogene	1
MALAT1	GAS5	processed_transcript	3
MALAT1	HLA-B	protein_coding	2
MALAT1	EEF1GP5	processed_pseudogene	1
MALAT1	RPS3AP6	processed_pseudogene	1
MALAT1	AC026784.1	transcribed_processed_pseudogene	1
MALAT1	BTG3P1	processed_pseudogene	1
MALAT1	RPS2P7	processed_pseudogene	1
MALAT1	LINC01553	lincRNA	1
MALAT1	LDHAP3	processed_pseudogene	1
MALAT1	RPL5P9	processed_pseudogene	1
MALAT1	RPL13AP5	processed_pseudogene	1
MALAT1	AC046176.1	processed_pseudogene	1
MALAT1	RPL30P4	processed_pseudogene	1
MALAT1	AC078991.1	processed_pseudogene	1
MALAT1	MTCO1P12	unprocessed_pseudogene	9
MALAT1	ACTG1P19	processed_pseudogene	1
MALAT1	LINC00426	lincRNA	1
MALAT1	RF00019	misc_RNA	1
MALAT1	RNU1-88P	snRNA	1
MALAT1	SCARNA7	snoRNA	1
MALAT1	RNU1-131P	snRNA	2
MALAT1	RNU1-80P	snRNA	3
MALAT1	SNORD13	snoRNA	1
MALAT1	TXNDC5	protein_coding	1
MALAT1	AC011979.1	processed_pseudogene	1
MALAT1	RPL32P34	processed_pseudogene	1
MALAT1	RN7SL573P	misc_RNA	1
MALAT1	AC073610.1	processed_pseudogene	1
MALAT1	IGKV3-20	IG_V_gene	1
MALAT1	AC104563.1	processed_pseudogene	1
MALAT1	RPSAP12	processed_pseudogene	1
MALAT1	AL132838.1	processed_pseudogene	2
MALAT1	AC132825.1	unprocessed_pseudogene	1
MALAT1	PPIL3	protein_coding	1
MALAT1	RPS12P28	processed_pseudogene	1
MALAT1	AL645465.1	antisense	1
MALAT1	RN7SL617P	misc_RNA	1
MALAT1	RN7SL70P	misc_RNA	1
MALAT1	AL136126.1	processed_pseudogene	1
MALAT1	CRCP	protein_coding	1
MALAT1	IGKV3-11	IG_V_gene	2
MALAT1	ATP5PO	protein_coding	1
MALAT1	RPL32P26	processed_pseudogene	1
MALAT1	PWP2	protein_coding	1
MALAT1	RN7SL566P	misc_RNA	1
MALAT1	RPL11P5	processed_pseudogene	1
MALAT1	AC108725.1	processed_pseudogene	1
MALAT1	AC100771.1	processed_pseudogene	1
MALAT1	RN7SL575P	misc_RNA	1
MALAT1	MRPL33	protein_coding	1
MALAT1	RPL30P14	processed_pseudogene	1
MALAT1	AC090543.2	processed_pseudogene	1
MALAT1	RPL5P30	processed_pseudogene	1
MALAT1	RPLP0P2	transcribed_processed_pseudogene	2
MALAT1	AC090589.1	processed_pseudogene	1
MALAT1	RPL5P14	processed_pseudogene	1
MALAT1	MRPS6	protein_coding	1
MALAT1	RPS6P22	processed_pseudogene	1
MALAT1	AC116533.1	processed_pseudogene	1
MALAT1	RPS26P43	processed_pseudogene	1
MALAT1	EEF1A1P4	processed_pseudogene	1
MALAT1	MTND4P12	processed_pseudogene	6
MALAT1	MTATP6P1	unprocessed_pseudogene	8
MALAT1	C5orf17	lincRNA	1
MALAT1	BCLAF1P1	processed_pseudogene	1
MALAT1	EEF1A1P9	processed_pseudogene	3
MALAT1	GAPDHP70	processed_pseudogene	1
MALAT1	AC093809.1	processed_pseudogene	2
MALAT1	CHCHD2P7	processed_pseudogene	1
MALAT1	AC068580.4	protein_coding	1
MALAT1	MTCYBP35	processed_pseudogene	2
MALAT1	SEMA3F	protein_coding	1
MALAT1	RBM5	protein_coding	1
MALAT1	SOX8	protein_coding	1
MALAT1	ITGA3	protein_coding	1
MALAT1	YBX2	protein_coding	1
MALAT1	PHTF2	protein_coding	1
MALAT1	ADIPOR2	protein_coding	1
MALAT1	PAFAH1B1	protein_coding	1
MALAT1	TEAD3	protein_coding	1
MALAT1	RPS20	protein_coding	1
MALAT1	UBE3C	protein_coding	1
MALAT1	MATR3	protein_coding	1
MALAT1	RUFY3	protein_coding	1
MALAT1	IKZF2	protein_coding	2
MALAT1	GAB2	protein_coding	1
MALAT1	MRI1	protein_coding	1
MALAT1	JADE2	protein_coding	1
MALAT1	TPR	protein_coding	1
MALAT1	VAMP3	protein_coding	1
MALAT1	SZRD1	protein_coding	1
MALAT1	GINM1	protein_coding	2
MALAT1	MRPL43	protein_coding	1
MALAT1	SEC61A1	protein_coding	1
MALAT1	POLR3E	protein_coding	1
MALAT1	BCAT1	protein_coding	1
MALAT1	CS	protein_coding	1
MALAT1	EIF4B	protein_coding	1
MALAT1	SPEN	protein_coding	2
MALAT1	SLK	protein_coding	1
MALAT1	NUCKS1	protein_coding	2
MALAT1	PABPC1	protein_coding	2
MALAT1	TCOF1	protein_coding	1
MALAT1	CPSF1	protein_coding	1
MALAT1	HSD17B10	protein_coding	1
MALAT1	FBXW11	protein_coding	1
MALAT1	SMARCE1	protein_coding	1
MALAT1	IGF2BP2	protein_coding	1
MALAT1	VDAC1P1	processed_pseudogene	1
MALAT1	CLASP1	protein_coding	1
MALAT1	MGLL	protein_coding	1
MALAT1	MYDGF	protein_coding	2
MALAT1	SLC25A3	protein_coding	1
MALAT1	RAB7A	protein_coding	1
MALAT1	TOP2B	protein_coding	1
MALAT1	TOLLIP	protein_coding	1
MALAT1	CIC	protein_coding	1
MALAT1	PGM1	protein_coding	1
MALAT1	KAT6A	protein_coding	1
MALAT1	APLP2	protein_coding	1
MALAT1	WBP11	protein_coding	1
MALAT1	KIF3C	protein_coding	1
MALAT1	SCAMP1	protein_coding	1
MALAT1	B4GALT1	protein_coding	1
MALAT1	FTL	protein_coding	2
MALAT1	SRRT	protein_coding	1
MALAT1	PSMC5	protein_coding	1
MALAT1	DOCK3	protein_coding	1
MALAT1	ATRN	protein_coding	1
MALAT1	RPLP0	protein_coding	1
MALAT1	PXN	protein_coding	1
MALAT1	AARS	protein_coding	1
MALAT1	GLG1	protein_coding	1
MALAT1	DTX2	protein_coding	1
MALAT1	JPH4	protein_coding	1
MALAT1	PSMD5	protein_coding	1
MALAT1	HSP90AB1	protein_coding	4
MALAT1	WASHC2A	protein_coding	1
MALAT1	ATP5F1D	protein_coding	1
MALAT1	PCDH11Y	protein_coding	1
MALAT1	POLRMT	protein_coding	1
MALAT1	MKNK2	protein_coding	1
MALAT1	PPIL2	protein_coding	1
MALAT1	EIF3L	protein_coding	2
MALAT1	SNU13	protein_coding	1
MALAT1	AP1B1	protein_coding	1
MALAT1	MYH9	protein_coding	3
MALAT1	NIN	protein_coding	1
MALAT1	HIF1A	protein_coding	2
MALAT1	PSMB5	protein_coding	1
MALAT1	ARHGAP5	protein_coding	1
MALAT1	CHD8	protein_coding	1
MALAT1	TM9SF1	protein_coding	1
MALAT1	SEC23A	protein_coding	1
MALAT1	SLCO4A1	protein_coding	1
MALAT1	POFUT1	protein_coding	1
MALAT1	VAPA	protein_coding	1
MALAT1	METTL4	protein_coding	1
MALAT1	MCTS2P	processed_pseudogene	1
MALAT1	SMARCA1	protein_coding	1
MALAT1	MAGT1	protein_coding	1
MALAT1	SLC25A15	protein_coding	1
MALAT1	DGKH	protein_coding	1
MALAT1	PSMD7	protein_coding	1
MALAT1	MON1B	protein_coding	1
MALAT1	CD276	protein_coding	1
MALAT1	EIF3E	protein_coding	1
MALAT1	TSTA3	protein_coding	1
MALAT1	MAN2B1	protein_coding	1
MALAT1	HNRNPL	protein_coding	1
MALAT1	SARS2	protein_coding	1
MALAT1	RPS16	protein_coding	2
MALAT1	PLIN3	protein_coding	1
MALAT1	RABAC1	protein_coding	1
MALAT1	KDELR1	protein_coding	1
MALAT1	TNPO2	protein_coding	1
MALAT1	GCDH	protein_coding	1
MALAT1	PMPCB	protein_coding	1
MALAT1	HBP1	protein_coding	1
MALAT1	MTPN	protein_coding	2
MALAT1	TAX1BP1	protein_coding	1
MALAT1	TMEM248	protein_coding	1
MALAT1	TMEM245	protein_coding	1
MALAT1	RAPGEF1	protein_coding	1
MALAT1	NPDC1	protein_coding	1
MALAT1	GATA3	protein_coding	1
MALAT1	CCDC6	protein_coding	1
MALAT1	RPL28	protein_coding	2
MALAT1	PPIF	protein_coding	1
MALAT1	NUFIP2	protein_coding	1
MALAT1	PSMD3	protein_coding	2
MALAT1	SLC25A11	protein_coding	1
MALAT1	SMARCD2	protein_coding	1
MALAT1	UTP6	protein_coding	1
MALAT1	YWHAE	protein_coding	3
MALAT1	EIF4G2	protein_coding	1
MALAT1	MLEC	protein_coding	1
MALAT1	RAD51AP1	protein_coding	1
MALAT1	OAS3	protein_coding	1
MALAT1	GAPDH	protein_coding	2
MALAT1	AL021546.1	protein_coding	1
MALAT1	MCM3	protein_coding	1
MALAT1	C6orf62	protein_coding	1
MALAT1	PPP2R5D	protein_coding	1
MALAT1	HMGCS1	protein_coding	1
MALAT1	HSPA9	protein_coding	1
MALAT1	TARS	protein_coding	1
MALAT1	LNPEP	protein_coding	1
MALAT1	RPL24	protein_coding	1
MALAT1	PLXNA1	protein_coding	1
MALAT1	EIF4G1	protein_coding	1
MALAT1	ACTR3	protein_coding	2
MALAT1	NRBP1	protein_coding	1
MALAT1	STAT1	protein_coding	1
MALAT1	RPL22	protein_coding	2
MALAT1	QSOX1	protein_coding	1
MALAT1	TROVE2	protein_coding	1
MALAT1	RCAN3	protein_coding	1
MALAT1	KMT2A	protein_coding	2
MALAT1	ABCD4	protein_coding	1
MALAT1	RBM25	protein_coding	1
MALAT1	NRDE2	protein_coding	1
MALAT1	RNF170	protein_coding	1
MALAT1	SCPEP1	protein_coding	1
MALAT1	SRGN	protein_coding	1
MALAT1	RBM19	protein_coding	1
MALAT1	CDK2	protein_coding	1
MALAT1	TUBA1B	protein_coding	2
MALAT1	OBSL1	protein_coding	1
MALAT1	SRSF6	protein_coding	1
MALAT1	TMEM189-UBE2V1	protein_coding	1
MALAT1	RRP36	protein_coding	2
MALAT1	SLC17A3	protein_coding	1
MALAT1	SOX4	protein_coding	1
MALAT1	BBS2	protein_coding	1
MALAT1	ABCC4	protein_coding	1
MALAT1	NT5C	protein_coding	1
MALAT1	RPL23	protein_coding	1
MALAT1	TRIP10	protein_coding	1
MALAT1	SYMPK	protein_coding	1
MALAT1	PSMF1	protein_coding	1
MALAT1	ROMO1	protein_coding	1
MALAT1	LRRC61	protein_coding	1
MALAT1	TICAM1	protein_coding	1
MALAT1	EMC6	protein_coding	1
MALAT1	MGAT3	protein_coding	1
MALAT1	RAC2	protein_coding	1
MALAT1	CALU	protein_coding	1
MALAT1	ARPP19	protein_coding	1
MALAT1	KIF1C	protein_coding	2
MALAT1	BCL2L2	protein_coding	1
MALAT1	DAD1	protein_coding	1
MALAT1	RHBDF2	protein_coding	1
MALAT1	AFDN	protein_coding	2
MALAT1	LSM4	protein_coding	1
MALAT1	JUND	protein_coding	1
MALAT1	PRRC2B	protein_coding	2
MALAT1	UBA1	protein_coding	1
MALAT1	EMC8	protein_coding	1
MALAT1	MRPS25	protein_coding	1
MALAT1	DIAPH1	protein_coding	1
MALAT1	NDFIP1	protein_coding	1
MALAT1	THOC6	protein_coding	1
MALAT1	TIMM10B	protein_coding	1
MALAT1	IMMT	protein_coding	1
MALAT1	PNISR	protein_coding	2
MALAT1	MATN2	protein_coding	1
MALAT1	RTN3	protein_coding	1
MALAT1	MKRN1	protein_coding	1
MALAT1	SBF2	protein_coding	2
MALAT1	ERG28	protein_coding	1
MALAT1	NOTCH2	protein_coding	1
MALAT1	ARF3	protein_coding	2
MALAT1	YWHAQ	protein_coding	1
MALAT1	NARS	protein_coding	1
MALAT1	DSC2	protein_coding	1
MALAT1	DSC3	protein_coding	1
MALAT1	NREP	protein_coding	1
MALAT1	PSAT1	protein_coding	1
MALAT1	DMTF1	protein_coding	1
MALAT1	ATP5MC2	protein_coding	1
MALAT1	DNAJC14	protein_coding	1
MALAT1	CDK4	protein_coding	1
MALAT1	RPL13AP25	processed_pseudogene	1
MALAT1	COG3	protein_coding	1
MALAT1	KDELR2	protein_coding	1
MALAT1	DDX56	protein_coding	1
MALAT1	DCAF7	protein_coding	1
MALAT1	RPL35	protein_coding	1
MALAT1	MYC	protein_coding	1
MALAT1	TLN1	protein_coding	1
MALAT1	RPS6	protein_coding	1
MALAT1	IRF4	protein_coding	1
MALAT1	NUMA1	protein_coding	2
MALAT1	RPLP1	protein_coding	1
MALAT1	RABGGTB	protein_coding	1
MALAT1	DBT	protein_coding	1
MALAT1	LRPPRC	protein_coding	1
MALAT1	DUSP5	protein_coding	1
MALAT1	ATIC	protein_coding	1
MALAT1	UBL7	protein_coding	1
MALAT1	PPA2	protein_coding	1
MALAT1	HADH	protein_coding	1
MALAT1	LRIG3	protein_coding	1
MALAT1	TMBIM6	protein_coding	3
MALAT1	SORD	protein_coding	1
MALAT1	IGF1R	protein_coding	2
MALAT1	SEC11A	protein_coding	1
MALAT1	RPS2	protein_coding	1
MALAT1	DEF8	protein_coding	1
MALAT1	TOB1	protein_coding	1
MALAT1	CSNK1D	protein_coding	1
MALAT1	BRD4	protein_coding	1
MALAT1	RPS11	protein_coding	1
MALAT1	RPL13A	protein_coding	3
MALAT1	RPL11	protein_coding	1
MALAT1	WDTC1	protein_coding	1
MALAT1	PPOX	protein_coding	1
MALAT1	HDGF	protein_coding	1
MALAT1	SF3B4	protein_coding	1
MALAT1	PIP5K1A	protein_coding	1
MALAT1	UBAP2L	protein_coding	1
MALAT1	ILF2	protein_coding	1
MALAT1	ARF1	protein_coding	2
MALAT1	CNIH4	protein_coding	1
MALAT1	PDIA6	protein_coding	1
MALAT1	TEX261	protein_coding	1
MALAT1	AMMECR1L	protein_coding	1
MALAT1	FANCD2	protein_coding	1
MALAT1	CNOT9	protein_coding	1
MALAT1	TCTA	protein_coding	1
MALAT1	MARCH6	protein_coding	1
MALAT1	LHFPL2	protein_coding	1
MALAT1	CXCL14	protein_coding	1
MALAT1	PCYOX1L	protein_coding	1
MALAT1	FAM193B	protein_coding	1
MALAT1	ABT1	protein_coding	1
MALAT1	RPL7L1	protein_coding	1
MALAT1	SLC18B1	protein_coding	1
MALAT1	SH3KBP1	protein_coding	1
MALAT1	NDUFB11	protein_coding	1
MALAT1	OGT	protein_coding	1
MALAT1	SIGMAR1	protein_coding	1
MALAT1	CDHR1	protein_coding	1
MALAT1	ADD3	protein_coding	1
MALAT1	RGS10	protein_coding	1
MALAT1	BTBD10	protein_coding	1
MALAT1	ALDOA	protein_coding	3
MALAT1	FREM2	protein_coding	1
MALAT1	ANKRD50	protein_coding	1
MALAT1	DST	protein_coding	1
MALAT1	TMEM123	protein_coding	1
MALAT1	MBNL1	protein_coding	1
MALAT1	HNRNPDL	protein_coding	1
MALAT1	UTRN	protein_coding	1
MALAT1	CARHSP1	protein_coding	2
MALAT1	PTPRD	protein_coding	1
MALAT1	TOMM70	protein_coding	1
MALAT1	UCHL1	protein_coding	2
MALAT1	CCDC144CP	transcribed_processed_pseudogene	1
MALAT1	AGPAT5	protein_coding	1
MALAT1	ZFYVE27	protein_coding	1
MALAT1	SLC16A1	protein_coding	1
MALAT1	LARP1	protein_coding	1
MALAT1	EEF1A1	protein_coding	2
MALAT1	UBE2L6	protein_coding	1
MALAT1	UTP14A	protein_coding	1
MALAT1	EIF4A2	protein_coding	1
MALAT1	SMG1	protein_coding	1
MALAT1	CPT2	protein_coding	1
MALAT1	CCNB2	protein_coding	1
MALAT1	RER1	protein_coding	1
MALAT1	C21orf59	protein_coding	1
MALAT1	LAD1	protein_coding	1
MALAT1	SIM2	protein_coding	1
MALAT1	RSPRY1	protein_coding	1
MALAT1	CBS	protein_coding	1
MALAT1	CSTB	protein_coding	1
MALAT1	DIP2A	protein_coding	1
MALAT1	ZER1	protein_coding	1
MALAT1	CRTC2	protein_coding	1
MALAT1	ANO10	protein_coding	1
MALAT1	RPL8	protein_coding	1
MALAT1	LSM12	protein_coding	1
MALAT1	LBHD1	protein_coding	1
MALAT1	SLAMF6	protein_coding	1
MALAT1	SCNM1	protein_coding	1
MALAT1	ATP1A1	protein_coding	1
MALAT1	CCT3	protein_coding	1
MALAT1	STT3B	protein_coding	1
MALAT1	SUCLG1	protein_coding	1
MALAT1	SLMAP	protein_coding	1
MALAT1	PRKCD	protein_coding	1
MALAT1	PBRM1	protein_coding	1
MALAT1	CDC25A	protein_coding	1
MALAT1	DCAF13	protein_coding	1
MALAT1	WASHC5	protein_coding	1
MALAT1	METTL2B	protein_coding	1
MALAT1	HGSNAT	protein_coding	1
MALAT1	ARF6	protein_coding	1
MALAT1	HDGFL3	protein_coding	1
MALAT1	MCM7	protein_coding	1
MALAT1	CENPV	protein_coding	1
MALAT1	YWHAB	protein_coding	1
MALAT1	PDIA3	protein_coding	1
MALAT1	NUDT21	protein_coding	1
MALAT1	DOLPP1	protein_coding	1
MALAT1	IGF2	protein_coding	1
MALAT1	ZNF646	protein_coding	1
MALAT1	TUBA1A	protein_coding	1
MALAT1	RCOR2	protein_coding	1
MALAT1	SRP68	protein_coding	1
MALAT1	SETD5	protein_coding	1
MALAT1	DDIT4	protein_coding	3
MALAT1	FEN1	protein_coding	1
MALAT1	SERINC2	protein_coding	1
MALAT1	PCSK9	protein_coding	1
MALAT1	AL669983.1	processed_pseudogene	1
MALAT1	PTK2	protein_coding	1
MALAT1	RNASE6	protein_coding	1
MALAT1	CLIC4	protein_coding	1
MALAT1	CKAP2L	protein_coding	1
MALAT1	HEXDC	protein_coding	1
MALAT1	TRAPPC1	protein_coding	1
MALAT1	RNF150	protein_coding	1
MALAT1	HOXD4	protein_coding	1
MALAT1	PDCD6IP	protein_coding	2
MALAT1	NFXL1	protein_coding	1
MALAT1	PYM1	protein_coding	1
MALAT1	ELOVL6	protein_coding	1
MALAT1	RASA4B	protein_coding	1
MALAT1	LSM3	protein_coding	1
MALAT1	RPS9	protein_coding	3
MALAT1	ZNF160	protein_coding	1
MALAT1	ATP6V0E2	protein_coding	1
MALAT1	TPPP	protein_coding	1
MALAT1	KRT13	protein_coding	1
MALAT1	KRCC1	protein_coding	1
MALAT1	AHSA2P	transcribed_unitary_pseudogene	1
MALAT1	VEGFB	protein_coding	1
MALAT1	JUP	protein_coding	1
MALAT1	DDX23	protein_coding	1
MALAT1	RPL4	protein_coding	2
MALAT1	RPL15	protein_coding	2
MALAT1	SRP72	protein_coding	1
MALAT1	MARCKSL1	protein_coding	2
MALAT1	RAB6A	protein_coding	1
MALAT1	RPL37AP8	processed_pseudogene	2
MALAT1	SHMT1	protein_coding	1
MALAT1	DEAF1	protein_coding	1
MALAT1	WDR73	protein_coding	1
MALAT1	TGIF1	protein_coding	1
MALAT1	TBL1XR1	protein_coding	1
MALAT1	ZNF518A	protein_coding	1
MALAT1	ZBTB41	protein_coding	1
MALAT1	RIC8A	protein_coding	1
MALAT1	RPL10P16	processed_pseudogene	2
MALAT1	GTPBP6	protein_coding	1
MALAT1	PARP10	protein_coding	1
MALAT1	COX5A	protein_coding	1
MALAT1	C17orf62	protein_coding	1
MALAT1	TUFM	protein_coding	2
MALAT1	RCC2	protein_coding	2
MALAT1	TIGD5	protein_coding	1
MALAT1	C14orf119	protein_coding	1
MALAT1	FO393411.1	processed_pseudogene	2
MALAT1	SSR4	protein_coding	1
MALAT1	EHMT1	protein_coding	1
MALAT1	MUC16	protein_coding	1
MALAT1	SETD2	protein_coding	1
MALAT1	RNF41	protein_coding	1
MALAT1	MRPS16	protein_coding	1
MALAT1	CACNB4	protein_coding	1
MALAT1	KPNA2	protein_coding	1
MALAT1	TTC3	protein_coding	1
MALAT1	GLUD2	protein_coding	1
MALAT1	AP2A2	protein_coding	2
MALAT1	GPC6	protein_coding	1
MALAT1	SMDT1	protein_coding	1
MALAT1	HSP90AB3P	processed_pseudogene	1
MALAT1	DDX41	protein_coding	1
MALAT1	DAZAP2	protein_coding	1
MALAT1	AC026410.1	processed_pseudogene	1
MALAT1	TSSC4	protein_coding	1
MALAT1	WDR27	protein_coding	1
MALAT1	RBM33	protein_coding	1
MALAT1	HSF1	protein_coding	1
MALAT1	RGPD2	protein_coding	1
MALAT1	HS6ST3	protein_coding	1
MALAT1	NR2F2	protein_coding	1
MALAT1	P4HB	protein_coding	1
MALAT1	ZFP36L1	protein_coding	2
MALAT1	KRT5	protein_coding	1
MALAT1	EDARADD	protein_coding	1
MALAT1	SEPT10	protein_coding	1
MALAT1	SMYD4	protein_coding	1
MALAT1	UBE2H	protein_coding	2
MALAT1	ZFP91	protein_coding	1
MALAT1	PTMA	protein_coding	2
MALAT1	HIST1H1C	protein_coding	1
MALAT1	TUBB4B	protein_coding	1
MALAT1	C19orf54	protein_coding	1
MALAT1	RPL10AP2	processed_pseudogene	1
MALAT1	CLDN4	protein_coding	1
MALAT1	TUBB	protein_coding	3
MALAT1	PPIA	protein_coding	1
MALAT1	C20orf204	protein_coding	1
MALAT1	SLC6A9	protein_coding	1
MALAT1	PCBP2	protein_coding	2
MALAT1	AC107956.1	processed_pseudogene	3
MALAT1	FAR1	protein_coding	1
MALAT1	PHF2	protein_coding	1
MALAT1	PSAP	protein_coding	1
MALAT1	RPL37A	protein_coding	3
MALAT1	MRPL42	protein_coding	2
MALAT1	MIER1	protein_coding	2
MALAT1	QRICH1	protein_coding	1
MALAT1	UBL5	protein_coding	1
MALAT1	MT-CO2	protein_coding	5
MALAT1	TOGARAM1	protein_coding	1
MALAT1	LDB1	protein_coding	1
MALAT1	MT-ND2	protein_coding	1
MALAT1	MT-ND5	protein_coding	1
MALAT1	ZNF277	protein_coding	2
MALAT1	MT-ATP6	protein_coding	3
MALAT1	RPL39	protein_coding	2
MALAT1	PJA2	protein_coding	1
MALAT1	RNU6-208P	snRNA	1
MALAT1	RN7SKP104	misc_RNA	1
MALAT1	RF00019	misc_RNA	1
MALAT1	RN7SKP95	misc_RNA	1
MALAT1	RNA5SP426	rRNA	1
MALAT1	RNA5SP358	rRNA	3
MALAT1	RNU5B-2P	snRNA	1
MALAT1	SNORA73B	snoRNA	1
MALAT1	RNU6-984P	snRNA	1
MALAT1	RNU1-19P	snRNA	1
MALAT1	RF00019	misc_RNA	1
MALAT1	RNA5SP382	rRNA	1
MALAT1	RNA5SP352	rRNA	2
MALAT1	SNORA63	snoRNA	1
MALAT1	RNU1-49P	snRNA	1
MALAT1	RNA5S17	rRNA	1
MALAT1	RNA5S4	rRNA	2
MALAT1	RNA5SP74	rRNA	1
MALAT1	RNA5-8SP2	rRNA	1
MALAT1	RF00019	misc_RNA	1
MALAT1	RNA5SP429	rRNA	1
MALAT1	SNORA74A	snoRNA	1
MALAT1	RNA5SP336	rRNA	4
MALAT1	RNY1	misc_RNA	1
MALAT1	RF00019	misc_RNA	1
MALAT1	RNU4-76P	snRNA	1
MALAT1	RNA5S2	rRNA	2
MALAT1	RNU6-7	snRNA	1
MALAT1	RNA5SP267	rRNA	1
MALAT1	RNU1-43P	snRNA	1
MALAT1	RNU1-100P	snRNA	2
MALAT1	RF00019	misc_RNA	1
MALAT1	RNA5S16	rRNA	1
MALAT1	RF00019	misc_RNA	1
MALAT1	RNU6-61P	snRNA	2
MALAT1	RN7SKP187	misc_RNA	2
MALAT1	RNA5S13	rRNA	2
MALAT1	SKIV2L	protein_coding	1
MALAT1	C6orf48	protein_coding	1
MALAT1	LSM2	protein_coding	1
MALAT1	CSNK2B	protein_coding	1
MALAT1	HLA-C	protein_coding	2
MALAT1	GABBR1	protein_coding	1
MALAT1	DCTN1	protein_coding	1
MALAT1	CCL4L1	protein_coding	1
MALAT1	E2F4	protein_coding	1
MALAT1	IPO7	protein_coding	1
MALAT1	MUC19	protein_coding	1
MALAT1	RNU1-27P	snRNA	5
MALAT1	RNU6-25P	snRNA	1
MALAT1	RNU6-672P	snRNA	1
MALAT1	RNVU1-18	snRNA	11
MALAT1	RNU6-116P	snRNA	1
MALAT1	RNU6-42P	snRNA	1
MALAT1	RNU6-4P	snRNA	1
MALAT1	RNU6-31P	snRNA	1
MALAT1	RNU6-905P	snRNA	1
MALAT1	RNA5SP122	rRNA	1
MALAT1	RNU6-18P	snRNA	1
MALAT1	RNU6-140P	snRNA	1
MALAT1	RNVU1-14	snRNA	2
MALAT1	RNU1-3	snRNA	6
MALAT1	SNORA66	snoRNA	1
MALAT1	MT-RNR2	Mt_rRNA	7
MALAT1	STK38L	protein_coding	1
MALAT1	MT-RNR1	Mt_rRNA	6
MALAT1	RF00568	snoRNA	1
MALAT1	MT-ND4L	protein_coding	1
MALAT1	RPL12P38	transcribed_processed_pseudogene	1
MALAT1	EEF1A1P16	processed_pseudogene	1
MALAT1	RPL18AP3	processed_pseudogene	1
MALAT1	TPT1P13	processed_pseudogene	1
MALAT1	RPLP0P6	processed_pseudogene	1
MALAT1	PPP1CB	protein_coding	1
MALAT1	RPL13P2	processed_pseudogene	2
MALAT1	RPL13AP7	processed_pseudogene	1
MALAT1	UBD	protein_coding	1
MALAT1	TAX1BP3	protein_coding	1
MALAT1	PAXIP1-AS2	antisense	1
MALAT1	RPS15AP1	processed_pseudogene	1
MALAT1	AL354710.1	processed_pseudogene	1
MALAT1	SRSF9P1	processed_pseudogene	1
MALAT1	AC011825.1	processed_pseudogene	1
MALAT1	RPL13P12	processed_pseudogene	1
MALAT1	AP000354.1	processed_pseudogene	1
MALAT1	RPL17-C18orf32	protein_coding	1
MALAT1	IFI30	protein_coding	2
MALAT1	RPL18AP8	processed_pseudogene	1
MALAT1	AL391416.1	processed_pseudogene	1
MALAT1	RPL21P119	processed_pseudogene	1
MALAT1	RNU6-908P	snRNA	1
MALAT1	RN7SKP281	misc_RNA	2
MALAT1	RF00019	misc_RNA	1
MALAT1	RNU6-628P	snRNA	1
MALAT1	RNA5SP225	rRNA	1
MALAT1	RF00019	misc_RNA	1
MALAT1	AC023157.1	processed_pseudogene	1
MALAT1	RPS8P10	unprocessed_pseudogene	2
MALAT1	SNHG14	processed_transcript	1
MALAT1	AC211485.1	processed_pseudogene	1
MALAT1	RPL8P2	processed_pseudogene	1
MALAT1	AL138785.1	processed_pseudogene	1
MALAT1	AC005515.1	transcribed_unprocessed_pseudogene	1
MALAT1	UQCRFS1P1	processed_pseudogene	1
MALAT1	GAPDHP73	processed_pseudogene	1
MALAT1	RPS14P4	processed_pseudogene	1
MALAT1	RPS4XP2	processed_pseudogene	2
MALAT1	AC007285.1	antisense	1
MALAT1	AC092035.1	processed_pseudogene	1
MALAT1	FTH1P16	processed_pseudogene	2
MALAT1	RPL3P2	processed_pseudogene	1
MALAT1	TPT1P2	processed_pseudogene	1
MALAT1	PPIAP19	processed_pseudogene	1
MALAT1	AC131235.1	processed_pseudogene	2
MALAT1	TXNP5	processed_pseudogene	1
MALAT1	MT-ATP8	protein_coding	2
MALAT1	AL158201.1	processed_pseudogene	1
MALAT1	FTLP17	processed_pseudogene	1
MALAT1	OST4	protein_coding	1
MALAT1	SNRPD2P1	processed_pseudogene	1
MALAT1	GDI2P1	processed_pseudogene	1
MALAT1	PNPT1P1	processed_pseudogene	1
MALAT1	MTND2P20	processed_pseudogene	1
MALAT1	AL450405.1	processed_pseudogene	1
MALAT1	RPL4P5	processed_pseudogene	1
MALAT1	AC078817.1	processed_pseudogene	1
MALAT1	TCEA1P2	processed_pseudogene	1
MALAT1	AL512488.1	sense_intronic	1
MALAT1	MTCO1P53	processed_pseudogene	1
MALAT1	ACTG1P10	processed_pseudogene	1
MALAT1	Z97353.1	processed_pseudogene	1
MALAT1	DLEU2	antisense	1
MALAT1	MTND4P24	processed_pseudogene	2
MALAT1	RPL4P2	processed_pseudogene	1
MALAT1	Z74021.1	processed_pseudogene	1
MALAT1	FTLP2	processed_pseudogene	1
MALAT1	ACTBP12	processed_pseudogene	1
MALAT1	BX842559.2	processed_pseudogene	1
MALAT1	AC007969.1	processed_pseudogene	1
MALAT1	RPS15AP11	processed_pseudogene	1
MALAT1	HSPA8P1	processed_pseudogene	1
MALAT1	AL592293.2	processed_pseudogene	1
MALAT1	XRCC6P2	processed_pseudogene	1
MALAT1	AL035456.1	processed_pseudogene	2
MALAT1	RPL10AP5	processed_pseudogene	1
MALAT1	KIAA0040	protein_coding	1
MALAT1	PKMP1	processed_pseudogene	1
MALAT1	AC099654.3	unprocessed_pseudogene	1
MALAT1	AC091685.2	processed_pseudogene	1
MALAT1	AL080243.2	processed_pseudogene	1
MALAT1	SMG1P1	transcribed_unprocessed_pseudogene	1
MALAT1	KIFC1	protein_coding	1
MALAT1	AMD1P2	processed_pseudogene	1
MALAT1	MTCO1P18	unprocessed_pseudogene	1
MALAT1	RNU6-893P	snRNA	1
MALAT1	RNU6-671P	snRNA	1
MALAT1	RN7SL87P	misc_RNA	1
MALAT1	RPL37P2	processed_pseudogene	2
MALAT1	RPS4XP22	processed_pseudogene	1
MALAT1	C1orf226	protein_coding	1
MALAT1	RN7SL674P	misc_RNA	1
MALAT1	RPS4XP13	processed_pseudogene	1
MALAT1	AC010343.1	processed_pseudogene	2
MALAT1	MTATP8P1	unprocessed_pseudogene	2
MALAT1	AP001024.1	processed_pseudogene	3
MALAT1	AC132217.1	3prime_overlapping_ncRNA	1
MALAT1	RN7SL128P	misc_RNA	1
MALAT1	RNA5-8S5	rRNA	14
MALAT1	ARHGAP8	protein_coding	1
MALAT1	PSMC1P1	processed_pseudogene	1
MALAT1	MTCO1P55	processed_pseudogene	1
MALAT1	RPL29P23	processed_pseudogene	1
MALAT1	RPL7AP6	processed_pseudogene	1
MALAT1	AC092597.1	processed_pseudogene	1
MALAT1	AC073861.1	processed_pseudogene	2
MALAT1	RPL12P32	processed_pseudogene	1
MALAT1	RN7SL685P	misc_RNA	1
MALAT1	HIST1H2APS2	transcribed_processed_pseudogene	1
MALAT1	RNA5-8S5	rRNA	13
MALAT1	MICAL3	protein_coding	1
MALAT1	RN7SL861P	misc_RNA	1
MALAT1	AC115223.1	processed_pseudogene	1
MALAT1	STMP1	protein_coding	1
MALAT1	RPS4XP14	processed_pseudogene	1
MALAT1	EEF1A1P10	processed_pseudogene	1
MALAT1	AP000942.1	processed_pseudogene	1
MALAT1	RN7SL610P	misc_RNA	1
MALAT1	TMEM199	protein_coding	1
MALAT1	P2RY11	protein_coding	1
MALAT1	RN7SL151P	misc_RNA	2
MALAT1	AC024293.1	processed_pseudogene	1
MALAT1	ENO1P1	transcribed_processed_pseudogene	1
MALAT1	UBE2V1	protein_coding	1
MALAT1	MTCYBP18	processed_pseudogene	2
MALAT1	NEAT1	lincRNA	4
MALAT1	PRR5-ARHGAP8	protein_coding	1
MALAT1	AC008758.3	transcribed_unprocessed_pseudogene	1
MALAT1	GAPDHP40	processed_pseudogene	1
MALAT1	MTND5P11	processed_pseudogene	5
MALAT1	AC025458.1	processed_pseudogene	1
MALAT1	EEF1A1P19	processed_pseudogene	1
MALAT1	YJEFN3	protein_coding	1
MALAT1	AC104619.3	processed_pseudogene	1
MALAT1	EEF1A1P13	processed_pseudogene	2
MALAT1	GAPDHP62	processed_pseudogene	1
MALAT1	AC024451.1	unprocessed_pseudogene	1
MALAT1	MTND5P12	processed_pseudogene	2
MALAT1	RNA5-8SP6	rRNA	2
MALAT1	RNU6-585P	snRNA	1
MALAT1	RNU6-346P	snRNA	1
MALAT1	RNY3P15	misc_RNA	1
MALAT1	RNA5SP348	rRNA	1
MALAT1	RNU6-1332P	snRNA	1
MALAT1	TRNP1	protein_coding	1
MALAT1	IGHGP	IG_C_pseudogene	1
MALAT1	LINC01933	lincRNA	1
MALAT1	AL136295.1	protein_coding	1
MALAT1	EEF1G	protein_coding	3
MALAT1	AP001646.2	processed_pseudogene	1
MALAT1	ZFP91-CNTF	protein_coding	1
MALAT1	MTCO1P15	processed_pseudogene	2
MALAT1	AP002990.1	protein_coding	3
MALAT1	Z82188.1	pseudogene	1
MALAT1	MTRNR2L3	protein_coding	1
MALAT1	HSPA8P5	processed_pseudogene	1
MALAT1	AP001888.1	processed_pseudogene	1
MALAT1	AP001453.4	lincRNA	1
MALAT1	P2RX5-TAX1BP3	protein_coding	1
MALAT1	AC091078.2	processed_pseudogene	1
MALAT1	AL355075.4	antisense	2
MALAT1	MTCO1P2	unprocessed_pseudogene	1
MALAT1	MTND5P32	processed_pseudogene	1
MALAT1	AL136295.4	protein_coding	1
MALAT1	AC023813.2	processed_pseudogene	1
MALAT1	ATP5PBP7	processed_pseudogene	1
MALAT1	TUBAP4	transcribed_processed_pseudogene	1
MALAT1	AC108134.2	lincRNA	1
MALAT1	MTCO3P24	unprocessed_pseudogene	1
MALAT1	MTCO1P40	processed_pseudogene	2
MALAT1	AC138761.1	transcribed_unprocessed_pseudogene	1
MALAT1	SNORD3A	snoRNA	1
MALAT1	AC073508.2	protein_coding	1
MALAT1	RN7SL230P	misc_RNA	2
MALAT1	TIMM23	protein_coding	2
MALAT1	RN7SL166P	misc_RNA	1
MALAT1	RPL17	protein_coding	1
MALAT1	RN7SL444P	misc_RNA	1
MALAT1	RASSF5	protein_coding	1
MALAT1	RNA28S5	rRNA	18
MALAT1	AKR1B1P7	processed_pseudogene	1
MALAT1	MTCO2P2	processed_pseudogene	1
MALAT1	ZNF224	protein_coding	1
MALAT1	AC007192.1	protein_coding	1
MALAT1	AC008758.4	protein_coding	1
MALAT1	MTRNR2L12	protein_coding	6
MALAT1	MTRNR2L11	protein_coding	1
MALAT1	MTND4LP5	processed_pseudogene	2
MALAT1	RNA18S5	rRNA	18
MALAT1	POM121C	protein_coding	1
MALAT1	AC093668.2	protein_coding	1
MALAT1	CBSL	protein_coding	1
MALAT1	SYNRG	protein_coding	1
MALAT1	DUSP14	protein_coding	1
MALAT1	UHRF1	protein_coding	1
MALAT1	CCL3L1	protein_coding	1
MALAT1	AC245014.3	lincRNA	2
MALAT1	HIST1H2AE	protein_coding	1
MALAT1	GAPDHP41	processed_pseudogene	1
MALAT1	AC009336.2	protein_coding	1
MALAT1	C11orf98	protein_coding	1
MALAT1	AC020765.3	unprocessed_pseudogene	1
MALAT1	AC135068.6	processed_pseudogene	2
MALAT1	AD000090.1	antisense	4
MALAT1	IGF2	protein_coding	1
MALAT1	ALDOA	protein_coding	3
MALAT1	hsa-miR-7641	miRNA	1
MALAT1	Met_tRNA	tRNA	1
MALAT1	Asp_tRNA	tRNA	1
MALAT1	Pseudo_tRNA	tRNA	1
MALAT1	His_tRNA	tRNA	1
MALAT1	Arg_tRNA	tRNA	1
MALAT1	His_tRNA	tRNA	1
MALAT1	Leu_tRNA	tRNA	1
MALAT1	Glu_tRNA	tRNA	1
MALAT1	Ala_tRNA	tRNA	1
MALAT1	Lys_tRNA	tRNA	1
MALAT1	Pseudo_tRNA	tRNA	1
MALAT1	Gly_tRNA	tRNA	1
MALAT1	RF00019	misc_RNA	3
MALAT1	RNU1-61P	snRNA	1
MALAT1	RNU2-35P	snRNA	2
MALAT1	RNY5	misc_RNA	1
MALAT1	RF00019	misc_RNA	1
MALAT1	RNU1-135P	snRNA	1
MALAT1	RNU2-46P	snRNA	1
MALAT1	RNU1-117P	snRNA	1
MALAT1	PRKDC	protein_coding	1
MALAT1	AC022861.1	processed_pseudogene	1
MALAT1	TMED10P1	processed_pseudogene	1
MALAT1	MTCYBP41	processed_pseudogene	1
MALAT1	PRR13P2	processed_pseudogene	1
MALAT1	BORCS8	protein_coding	1
MALAT1	AP000781.2	protein_coding	1
MALAT1	DPP3	protein_coding	1
MALAT1	BRK1	protein_coding	2
MALAT1	AC018523.1	processed_pseudogene	1
MALAT1	PGAM1P8	transcribed_processed_pseudogene	2
MALAT1	NPM1P35	processed_pseudogene	1
MALAT1	AL133352.1	protein_coding	1
MALAT1	MTRNR2L8	protein_coding	13
MALAT1	AP000763.2	processed_pseudogene	1
MALAT1	ATF4P4	transcribed_processed_pseudogene	1
MALAT1	SUPT16HP1	processed_pseudogene	1
MALAT1	RPL41P5	processed_pseudogene	1
MALAT1	AC008731.1	pseudogene	1
MALAT1	AL139819.1	pseudogene	1
MALAT1	AP003108.2	protein_coding	1
MALAT1	MTRNR2L1	protein_coding	4
MALAT1	AC140481.3	pseudogene	1
MALAT1	AL021707.2	pseudogene	1
MALAT1	EIF3LP1	processed_pseudogene	1
MALAT1	PPIAP4	processed_pseudogene	1
MALAT1	AC104390.1	processed_pseudogene	1
MALAT1	BLOC1S5-TXNDC5	protein_coding	1
MALAT1	AC087632.1	protein_coding	1
MALAT1	EEF1A1P22	processed_pseudogene	1
MALAT1	AC091167.2	protein_coding	1
MALAT1	AC007906.1	sense_intronic	2
MALAT1	AC010542.4	lincRNA	1
MALAT1	AC138894.1	protein_coding	1
MALAT1	SNORD3B-2	snoRNA	1
MALAT1	AC010547.6	unprocessed_pseudogene	1
MALAT1	AC007952.4	lincRNA	3
MALAT1	GTF2I	protein_coding	2
MALAT1	MTCO3P13	unprocessed_pseudogene	1
MALAT1	SNORD3C	snoRNA	4
MALAT1	SNORD3B-1	snoRNA	3
MALAT1	AC005899.4	processed_transcript	1
MALAT1	MTCYBP13	unprocessed_pseudogene	1
MALAT1	AP000902.1	processed_pseudogene	1
MALAT1	ACTBP9	processed_pseudogene	1
MALAT1	S1PR2	protein_coding	1
MALAT1	AC011503.2	sense_intronic	1
MALAT1	SNHG8	lincRNA	1
MALAT1	AL356488.2	lincRNA	1
MALAT1	AL360012.1	lincRNA	1
MALAT1	AL591806.3	protein_coding	1
MALAT1	MTCYBP22	processed_pseudogene	1
MALAT1	MTCO3P22	processed_pseudogene	2
MALAT1	MTND4P35	processed_pseudogene	2
MALAT1	MTCO1P22	processed_pseudogene	2
MALAT1	MTND5P10	processed_pseudogene	3
MALAT1	SNORD14A	snoRNA	1
MALAT1	RN7SL828P	misc_RNA	1
MALAT1	RNA5SP506	rRNA	1
MALAT1	AL135925.1	lincRNA	1
MALAT1	AC073610.3	protein_coding	1
MALAT1	SNURF	protein_coding	1
MALAT1	AC019176.2	unprocessed_pseudogene	1
MALAT1	FP565260.1	protein_coding	1
MALAT1	HIST1H3A	protein_coding	1
MALAT1	RIMBP3	protein_coding	1
MALAT1	SRCIN1	protein_coding	1
MALAT1	MARCKS	protein_coding	2
MALAT1	AC090498.1	processed_pseudogene	1
MALAT1	AL022311.1	sense_overlapping	1
MALAT1	SLFNL1-AS1	antisense	1
MALAT1	BLACAT1	lincRNA	1
MALAT1	AL157392.5	protein_coding	1
MALAT1	AL139099.4	non_coding	2
MALAT1	AC008038.1	processed_pseudogene	1
MALAT1	AC006511.5	processed_transcript	1
MALAT1	AP003175.1	processed_transcript	1
MALAT1	AC068946.2	protein_coding	1
MALAT1	AC068831.7	protein_coding	1
MALAT1	AL358113.1	protein_coding	1
MALAT1	RNA18N5	rRNA	20
MALAT1	hsa-miR-4485	miRNA	1
MALAT1	Met_tRNA	tRNA	2
MALAT1	Thr_tRNA	tRNA	1
MALAT1	Leu_tRNA	tRNA	1
MALAT1	Cys_tRNA	tRNA	1
MALAT1	Glu_tRNA	tRNA	1
MALAT1	Asp_tRNA	tRNA	1
MALAT1	Met_tRNA	tRNA	2
MALAT1	Arg_tRNA	tRNA	1
MALAT1	Leu_tRNA	tRNA	1
MALAT1	Gly_tRNA	tRNA	1
MALAT1	Asp_tRNA	tRNA	1
MALAT1	Asp_tRNA	tRNA	1
MEG3	AKAP11	protein_coding	1
MEG3	IDH1	protein_coding	1
TUG1	SPAST	protein_coding	1
TUG1	ZNF839	protein_coding	1
TUG1	CDH1	protein_coding	1
TUG1	FOXN3	protein_coding	1
TUG1	SLC2A3	protein_coding	1
TUG1	VPS35	protein_coding	1
TUG1	RPL31	protein_coding	1
TUG1	TRIP13	protein_coding	1
TUG1	SLC1A3	protein_coding	1
TUG1	STK17B	protein_coding	1
TUG1	ITPKC	protein_coding	1
TUG1	AGO1	protein_coding	1
TUG1	MSH2	protein_coding	1
TUG1	ATP5F1D	protein_coding	2
TUG1	RANBP1	protein_coding	1
TUG1	SNRPD3	protein_coding	1
TUG1	GTPBP1	protein_coding	1
TUG1	ADNP	protein_coding	1
TUG1	HAS3	protein_coding	1
TUG1	NOMO3	protein_coding	1
TUG1	TTC23	protein_coding	1
TUG1	TUBB4A	protein_coding	1
TUG1	HSPA8	protein_coding	1
TUG1	PTGES3	protein_coding	1
TUG1	SUDS3	protein_coding	1
TUG1	SRSF3	protein_coding	1
TUG1	MDGA1	protein_coding	1
TUG1	SRF	protein_coding	1
TUG1	TMCO6	protein_coding	1
TUG1	APH1A	protein_coding	1
TUG1	PRRC2C	protein_coding	1
TUG1	NEK2	protein_coding	1
TUG1	YIPF4	protein_coding	1
TUG1	PROSER1	protein_coding	1
TUG1	NCOA5	protein_coding	1
TUG1	NCLN	protein_coding	1
TUG1	NUP214	protein_coding	1
TUG1	HP1BP3	protein_coding	1
TUG1	SPCS3	protein_coding	1
TUG1	DKC1	protein_coding	1
TUG1	DIAPH1	protein_coding	1
TUG1	TRAF7	protein_coding	1
TUG1	TPT1	protein_coding	1
TUG1	LDHA	protein_coding	1
TUG1	BTF3L4	protein_coding	1
TUG1	GDF11	protein_coding	1
TUG1	HNRNPA1	protein_coding	1
TUG1	RPS6	protein_coding	1
TUG1	HMGA1	protein_coding	2
TUG1	MTCH1	protein_coding	1
TUG1	BUD13	protein_coding	1
TUG1	SMAD6	protein_coding	1
TUG1	HNRNPA1L2	protein_coding	1
TUG1	ZFHX3	protein_coding	1
TUG1	MED9	protein_coding	1
TUG1	NPEPPS	protein_coding	1
TUG1	NARF	protein_coding	1
TUG1	WDR45B	protein_coding	1
TUG1	SH3GL1	protein_coding	1
TUG1	NECTIN4	protein_coding	1
TUG1	RPS27A	protein_coding	2
TUG1	MARCH6	protein_coding	1
TUG1	FADS1	protein_coding	1
TUG1	VPS26B	protein_coding	1
TUG1	PABPC3	protein_coding	1
TUG1	TIAL1	protein_coding	1
TUG1	UHMK1	protein_coding	1
TUG1	HNRNPU	protein_coding	1
TUG1	NPTN	protein_coding	1
TUG1	CUL4B	protein_coding	1
TUG1	PKNOX1	protein_coding	1
TUG1	RPL8	protein_coding	1
TUG1	SRSF2	protein_coding	1
TUG1	RNASEK-C17orf49	protein_coding	1
TUG1	TKT	protein_coding	1
TUG1	MELTF	protein_coding	1
TUG1	MAD2L1	protein_coding	1
TUG1	RHOBTB3	protein_coding	1
TUG1	AQP3	protein_coding	1
TUG1	SPRED1	protein_coding	1
TUG1	EEF2	protein_coding	1
TUG1	RPSA	protein_coding	1
TUG1	SPRY3	protein_coding	1
TUG1	CHRNA5	protein_coding	1
TUG1	HNRNPF	protein_coding	1
TUG1	RPS9	protein_coding	1
TUG1	BNC2	protein_coding	1
TUG1	TRMT112	protein_coding	1
TUG1	ATAD5	protein_coding	1
TUG1	EPS8L2	protein_coding	1
TUG1	MAF	protein_coding	1
TUG1	SKIDA1	protein_coding	1
TUG1	RAP2B	protein_coding	1
TUG1	EPHA10	protein_coding	1
TUG1	BTN3A2	protein_coding	1
TUG1	PTMA	protein_coding	1
TUG1	ZNF292	protein_coding	1
TUG1	ZNF470	protein_coding	1
TUG1	MCMBP	protein_coding	1
TUG1	MT-ND4	protein_coding	1
TUG1	RPL39	protein_coding	1
TUG1	RNU1-14P	snRNA	1
TUG1	AL021707.1	pseudogene	1
TUG1	CCNI2	protein_coding	1
TUG1	HACD2	protein_coding	1
TUG1	SNORA80E	snoRNA	1
TUG1	MT-RNR2	Mt_rRNA	1
TUG1	MT-RNR1	Mt_rRNA	1
TUG1	AC064799.1	processed_pseudogene	1
TUG1	TCTEX1D2	protein_coding	1
TUG1	KLHL23	protein_coding	1
TUG1	RPLP0P6	processed_pseudogene	1
TUG1	EMP2	protein_coding	1
TUG1	AC005005.1	processed_pseudogene	1
TUG1	PTMAP5	transcribed_processed_pseudogene	1
TUG1	AP000343.1	processed_pseudogene	1
TUG1	AL139100.1	processed_pseudogene	1
TUG1	RPL23P8	processed_pseudogene	1
TUG1	FTH1P8	processed_pseudogene	1
TUG1	AL161787.1	processed_pseudogene	1
TUG1	FTH1P10	transcribed_processed_pseudogene	1
TUG1	HNRNPA1P48	protein_coding	1
TUG1	FGD5-AS1	antisense	1
TUG1	TPI1P1	processed_pseudogene	1
TUG1	MTCO2P12	unprocessed_pseudogene	1
TUG1	TFAP2A-AS1	antisense	1
TUG1	RPS23P8	processed_pseudogene	1
TUG1	AC125238.2	processed_pseudogene	1
TUG1	SRRM1P3	processed_pseudogene	1
TUG1	MTCO1P12	unprocessed_pseudogene	1
TUG1	ACTG1P19	processed_pseudogene	1
TUG1	C1orf226	protein_coding	1
TUG1	RNA5-8S5	rRNA	2
TUG1	RBBP4P2	processed_pseudogene	1
TUG1	RNA5-8S5	rRNA	1
TUG1	EEF1A1P4	processed_pseudogene	1
TUG1	RBBP4P1	processed_pseudogene	1
TUG1	RNY5	misc_RNA	1
TUG1	RALA	protein_coding	1
TUG1	MATR3	protein_coding	1
TUG1	RRAGD	protein_coding	1
TUG1	RSF1	protein_coding	1
TUG1	IPO5	protein_coding	1
TUG1	NTN1	protein_coding	1
TUG1	ZFAT	protein_coding	1
TUG1	TFRC	protein_coding	1
TUG1	ITM2A	protein_coding	1
TUG1	TNPO1	protein_coding	1
TUG1	FTL	protein_coding	1
TUG1	GRAMD1A	protein_coding	1
TUG1	HNRNPC	protein_coding	1
TUG1	BAMBI	protein_coding	1
TUG1	HSP90AB1	protein_coding	2
TUG1	ACOT7	protein_coding	1
TUG1	SCD	protein_coding	1
TUG1	EP300	protein_coding	1
TUG1	SNW1	protein_coding	1
TUG1	VAPA	protein_coding	1
TUG1	CENPT	protein_coding	1
TUG1	TSC2	protein_coding	1
TUG1	OAZ1	protein_coding	2
TUG1	TMEM147	protein_coding	1
TUG1	GRB10	protein_coding	1
TUG1	CCDC6	protein_coding	1
TUG1	YWHAE	protein_coding	2
TUG1	LPXN	protein_coding	1
TUG1	ATP5F1B	protein_coding	1
TUG1	FXR1	protein_coding	1
TUG1	SCAP	protein_coding	1
TUG1	KIAA2013	protein_coding	1
TUG1	MFN2	protein_coding	1
TUG1	BCAS2	protein_coding	1
TUG1	MED28	protein_coding	1
TUG1	CCNI	protein_coding	1
TUG1	CCND2	protein_coding	1
TUG1	IRF2BPL	protein_coding	1
TUG1	TCP1	protein_coding	1
TUG1	KHDRBS1	protein_coding	1
TUG1	TUBA1B	protein_coding	1
TUG1	STK35	protein_coding	1
TUG1	UQCR11	protein_coding	1
TUG1	PODXL	protein_coding	1
TUG1	PSMA1	protein_coding	1
TUG1	APOE	protein_coding	1
TUG1	PPFIA1	protein_coding	1
TUG1	H3F3B	protein_coding	1
TUG1	SPART	protein_coding	1
TUG1	ERG28	protein_coding	1
TUG1	EPC2	protein_coding	1
TUG1	SCRN1	protein_coding	1
TUG1	DBNL	protein_coding	1
TUG1	RSAD1	protein_coding	1
TUG1	PPM1B	protein_coding	1
TUG1	RPS24	protein_coding	1
TUG1	SINHCAF	protein_coding	1
TUG1	MFAP1	protein_coding	1
TUG1	IGFBP4	protein_coding	1
TUG1	ILF2	protein_coding	1
TUG1	RPS3	protein_coding	1
TUG1	ALDOA	protein_coding	1
TUG1	PLEKHG4B	protein_coding	1
TUG1	MARVELD1	protein_coding	1
TUG1	SLC16A1	protein_coding	1
TUG1	EEF1A1	protein_coding	1
TUG1	RNF166	protein_coding	1
TUG1	UBR1	protein_coding	1
TUG1	NAE1	protein_coding	1
TUG1	CBS	protein_coding	1
TUG1	LAPTM5	protein_coding	1
TUG1	ICA1L	protein_coding	1
TUG1	CTSB	protein_coding	1
TUG1	CCT2	protein_coding	1
TUG1	TMEM41B	protein_coding	1
TUG1	NDUFS5	protein_coding	1
TUG1	HNRNPA3	protein_coding	1
TUG1	ELP5	protein_coding	1
TUG1	TMEM43	protein_coding	1
TUG1	RPL38	protein_coding	1
TUG1	VANGL1	protein_coding	1
TUG1	MZT2A	protein_coding	1
TUG1	IMP3	protein_coding	1
TUG1	RPS12P23	processed_pseudogene	1
TUG1	FO393411.1	processed_pseudogene	1
TUG1	ZDHHC20	protein_coding	1
TUG1	SHMT2	protein_coding	1
TUG1	RPL27AP5	processed_pseudogene	1
TUG1	MTA1	protein_coding	1
TUG1	RIMBP3C	protein_coding	1
TUG1	SUMO3	protein_coding	1
TUG1	BRWD1	protein_coding	1
TUG1	EDARADD	protein_coding	1
TUG1	RPS23	protein_coding	1
TUG1	BLOC1S2	protein_coding	1
TUG1	EEF1A1P5	processed_pseudogene	1
TUG1	TUBB	protein_coding	1
TUG1	C5orf42	protein_coding	1
TUG1	CDC42SE1	protein_coding	1
TUG1	RPL37A	protein_coding	2
TUG1	ADH5	protein_coding	1
TUG1	ZNF649	protein_coding	1
TUG1	ZNF251	protein_coding	1
TUG1	PPIAP22	processed_pseudogene	1
TUG1	RNU1-134P	snRNA	1
TUG1	ZDHHC18	protein_coding	1
TUG1	BAG6	protein_coding	1
TUG1	RNVU1-7	snRNA	1
TUG1	SNORD15A	snoRNA	1
TUG1	RNU6-30P	snRNA	1
TUG1	RNVU1-1	snRNA	1
TUG1	HNRNPA1P40	processed_pseudogene	1
TUG1	RPS29	protein_coding	1
TUG1	EEF1A1P12	processed_pseudogene	1
TUG1	HNRNPA1P7	processed_pseudogene	1
TUG1	RN7SKP221	misc_RNA	1
TUG1	TMSB4XP4	processed_pseudogene	1
TUG1	HLA-DPB1	protein_coding	1
TUG1	RPS8P10	unprocessed_pseudogene	1
TUG1	HSP90AA2P	processed_pseudogene	1
TUG1	ATXN1L	protein_coding	1
TUG1	NDUFAF8	protein_coding	1
TUG1	MTND2P28	unprocessed_pseudogene	1
TUG1	AC067942.1	transcribed_processed_pseudogene	1
TUG1	RPL9P8	pseudogene	1
TUG1	RPL36AP39	processed_pseudogene	1
TUG1	RPL3P12	processed_pseudogene	1
TUG1	EEF1A1P6	processed_pseudogene	2
TUG1	TUBAP	processed_pseudogene	1
TUG1	RPL9P7	processed_pseudogene	1
TUG1	TMEM250	protein_coding	1
TUG1	AL133163.1	processed_pseudogene	1
TUG1	AC098831.1	processed_pseudogene	1
TUG1	RN7SL306P	misc_RNA	1
TUG1	SLC16A1P1	processed_pseudogene	1
TUG1	RPS23P1	processed_pseudogene	1
TUG1	ENO1P1	transcribed_processed_pseudogene	1
TUG1	RPL18P13	processed_pseudogene	1
TUG1	MTND5P11	processed_pseudogene	1
TUG1	AC024451.3	processed_pseudogene	1
TUG1	MTRNR2L1	protein_coding	1
TUG1	HMGB1P6	processed_pseudogene	1
TUG1	AC024619.4	processed_pseudogene	1
TUG1	RNA28S5	rRNA	7
TUG1	AC090004.1	protein_coding	1
TUG1	MTCYBP22	processed_pseudogene	1
TUG1	RNA18S5	rRNA	4
TUG1	CBSL	protein_coding	1
TUG1	BACE1-AS	antisense	1
TUG1	MATR3	protein_coding	1
TUG1	AC135068.6	processed_pseudogene	1
TUG1	ALDOA	protein_coding	1
TUG1	C17orf49	protein_coding	1
TUG1	AL049873.2	processed_pseudogene	1
TUG1	AC011477.5	processed_pseudogene	1
TUG1	MTCO3P22	processed_pseudogene	1
TUG1	AC069257.3	protein_coding	1
TUG1	AC005258.1	protein_coding	1
TUG1	AD000090.1	antisense	1
TUG1	RNA18N5	rRNA	5

**Table S2:** Significant KEGG pathways and GO biological processes regulated by the protein coding gene targets of the dysregulated lncRNAs identified by two or more studies

KEGG pathway ID	Description	GeneRatio	BgRatio	pvalue	qvalue	geneID
hsa03010	Ribosome	8/43	167/8225	1,93E-06	0,000103	RPL3/RPS13/RPS3/RPSA/RPLP2/RPS23/RPS18/RPS27A
hsa05171	Coronavirus disease - COVID-19	9/43	232/8225	2,36E-06	0,000103	RPL3/RPS13/OAS2/RPS3/RPSA/RPLP2/RPS23/RPS18/RPS27A
hsa00010	Glycolysis / Gluconeogenesis	4/43	67/8225	0,000391	0,010507	PKM/ENO1/PGK1/TPI1
hsa04520	Adherens junction	4/43	71/8225	0,000488	0,010507	ACTB/ACTG1/PTPN1/CTNND1
hsa01230	Biosynthesis of amino acids	4/43	75/8225	0,000601	0,010507	PKM/ENO1/PGK1/TPI1
hsa04810	Regulation of actin cytoskeleton	6/43	229/8225	0,001122	0,016344	ACTB/CFL1/ACTG1/ARHGEF12/TMSB4X/BRK1
hsa05014	Amyotrophic lateral sclerosis	7/43	364/8225	0,002548	0,0318	ACTB/FUS/COX6A1/HNRNPA2B1/NDUFB8/ACTG1/ATP5F1D
hsa01200	Carbon metabolism	4/43	115/8225	0,00294	0,03211	PKM/ENO1/PGK1/TPI1
hsa05130	Pathogenic *Escherichia coli* infection	5/43	197/8225	0,003444	0,032343	ACTB/RPS3/ACTG1/ARHGEF12/BRK1
hsa05416	Viral myocarditis	3/43	60/8225	0,003702	0,032343	ACTB/ACTG1/HLA-B
GO biological process	Description	GeneRatio	BgRatio	pvalue	qvalue	geneID
GO:0006614	SRP-dependent cotranslational protein targeting to membrane	8/63	105/18862	2,12E-09	1,6E-06	RPL3/RPS13/RPS3/RPSA/RPLP2/RPS23/RPS18/RPS27A
GO:0006613	cotranslational protein targeting to membrane	8/63	110/18862	3,07E-09	1,6E-06	RPL3/RPS13/RPS3/RPSA/RPLP2/RPS23/RPS18/RPS27A
GO:0000184	nuclear-transcribed mRNA catabolic process, nonsense-mediated decay	8/63	120/18862	6,13E-09	1,6E-06	RPL3/RPS13/RPS3/RPSA/RPLP2/RPS23/RPS18/RPS27A
GO:0045047	protein targeting to ER	8/63	120/18862	6,13E-09	1,6E-06	RPL3/RPS13/RPS3/RPSA/RPLP2/RPS23/RPS18/RPS27A
GO:0072599	establishment of protein localization to endoplasmic reticulum	8/63	124/18862	7,95E-09	1,66E-06	RPL3/RPS13/RPS3/RPSA/RPLP2/RPS23/RPS18/RPS27A
GO:0006401	RNA catabolic process	12/63	414/18862	1,04E-08	1,81E-06	FUS/RPL3/HSPA8/RPS13/OAS2/RPS3/IGF2BP1/RPSA/RPLP2/RPS23/RPS18/RPS27A
GO:0006413	translational initiation	9/63	193/18862	1,5E-08	2,24E-06	RPL3/RPS13/EIF4EBP2/RPS3/RPSA/RPLP2/RPS23/RPS18/RPS27A
GO:0070972	protein localization to endoplasmic reticulum	8/63	152/18862	3,93E-08	4,7E-06	RPL3/RPS13/RPS3/RPSA/RPLP2/RPS23/RPS18/RPS27A
GO:0006402	mRNA catabolic process	11/63	375/18862	4,04E-08	4,7E-06	FUS/RPL3/HSPA8/RPS13/RPS3/IGF2BP1/RPSA/RPLP2/RPS23/RPS18/RPS27A
GO:0019083	viral transcription	8/63	180/18862	1,46E-07	1,52E-05	RPL3/RPS13/RPS3/RPSA/RPLP2/RPS23/RPS18/RPS27A
GO:0019080	viral gene expression	8/63	198/18862	3,02E-07	2,88E-05	RPL3/RPS13/RPS3/RPSA/RPLP2/RPS23/RPS18/RPS27A
GO:0000956	nuclear-transcribed mRNA catabolic process	8/63	208/18862	4,4E-07	3,84E-05	RPL3/RPS13/RPS3/RPSA/RPLP2/RPS23/RPS18/RPS27A
GO:0006612	protein targeting to membrane	8/63	211/18862	4,91E-07	3,95E-05	RPL3/RPS13/RPS3/RPSA/RPLP2/RPS23/RPS18/RPS27A
GO:0046034	ATP metabolic process	9/63	313/18862	9,17E-07	6,85E-05	PKM/ENO1/PGK1/HSPA8/TPI1/COX6A1/NDUFB8/TMSB4X/ATP5F1D
GO:0006735	NADH regeneration	4/63	27/18862	1,87E-06	0,000115	PKM/ENO1/PGK1/TPI1
GO:0061621	canonical glycolysis	4/63	27/18862	1,87E-06	0,000115	PKM/ENO1/PGK1/TPI1
GO:0061718	glucose catabolic process to pyruvate	4/63	27/18862	1,87E-06	0,000115	PKM/ENO1/PGK1/TPI1
GO:0061620	glycolytic process through glucose-6-phosphate	4/63	28/18862	2,18E-06	0,000127	PKM/ENO1/PGK1/TPI1
GO:0061615	glycolytic process through fructose-6-phosphate	4/63	29/18862	2,52E-06	0,000139	PKM/ENO1/PGK1/TPI1
GO:0098974	postsynaptic actin cytoskeleton organization	3/63	11/18862	5,75E-06	0,000301	ACTB/DBN1/ACTG1
GO:0006007	glucose catabolic process	4/63	36/18862	6,14E-06	0,000306	PKM/ENO1/PGK1/TPI1
GO:0099188	postsynaptic cytoskeleton organization	3/63	13/18862	9,92E-06	0,000472	ACTB/DBN1/ACTG1
GO:0006734	NADH metabolic process	4/63	43/18862	1,26E-05	0,000575	PKM/ENO1/PGK1/TPI1
GO:0006605	protein targeting	9/63	441/18862	1,49E-05	0,000649	RPL3/HSPA8/RPS13/RPS3/RPSA/RPLP2/RPS23/RPS18/RPS27A
GO:0090150	establishment of protein localization to membrane	8/63	354/18862	2,24E-05	0,000924	RPL3/RPS13/RPS3/RPSA/RPLP2/RPS23/RPS18/RPS27A
GO:0002181	cytoplasmic translation	5/63	102/18862	2,3E-05	0,000924	PKM/EEF2/RPSA/RPLP2/RPS23
GO:0019674	NAD metabolic process	4/63	51/18862	2,51E-05	0,000973	PKM/ENO1/PGK1/TPI1
GO:0006754	ATP biosynthetic process	4/63	56/18862	3,64E-05	0,001315	PKM/ENO1/TMSB4X/ATP5F1D
GO:0019320	hexose catabolic process	4/63	56/18862	3,64E-05	0,001315	PKM/ENO1/PGK1/TPI1
GO:0046365	monosaccharide catabolic process	4/63	62/18862	5,45E-05	0,001901	PKM/ENO1/PGK1/TPI1
GO:0009206	purine ribonucleoside triphosphate biosynthetic process	4/63	67/18862	7,4E-05	0,002497	PKM/ENO1/TMSB4X/ATP5F1D
GO:0009145	purine nucleoside triphosphate biosynthetic process	4/63	68/18862	7,84E-05	0,002563	PKM/ENO1/TMSB4X/ATP5F1D
GO:0009201	ribonucleoside triphosphate biosynthetic process	4/63	73/18862	0,000103	0,003281	PKM/ENO1/TMSB4X/ATP5F1D
GO:0006090	pyruvate metabolic process	5/63	150/18862	0,000144	0,004295	PKM/ENO1/PGK1/TPI1/BSG
GO:0001895	retina homeostasis	4/63	80/18862	0,000148	0,004295	ACTB/POTEF/B2M/ACTG1
GO:0009205	purine ribonucleoside triphosphate metabolic process	4/63	80/18862	0,000148	0,004295	PKM/ENO1/TMSB4X/ATP5F1D
GO:0009142	nucleoside triphosphate biosynthetic process	4/63	84/18862	0,000178	0,005047	PKM/ENO1/TMSB4X/ATP5F1D
GO:0009144	purine nucleoside triphosphate metabolic process	4/63	86/18862	0,000195	0,005346	PKM/ENO1/TMSB4X/ATP5F1D
GO:0099173	postsynapse organization	5/63	161/18862	0,000201	0,005346	ACTB/HSPA8/DBN1/CFL1/ACTG1
GO:0009199	ribonucleoside triphosphate metabolic process	4/63	87/18862	0,000204	0,005346	PKM/ENO1/TMSB4X/ATP5F1D
GO:1902903	regulation of supramolecular fiber organization	7/63	370/18862	0,000227	0,005799	COTL1/HSPA8/DBN1/RPS3/ACTG1/TMSB4X/BRK1
GO:0060333	interferon-gamma-mediated signaling pathway	4/63	91/18862	0,000243	0,006052	SP100/OAS2/B2M/HLA-B
GO:0060337	type I interferon signaling pathway	4/63	95/18862	0,000287	0,006972	SP100/OAS2/PTPN1/HLA-B
GO:0071357	cellular response to type I interferon	4/63	96/18862	0,000298	0,007092	SP100/OAS2/PTPN1/HLA-B
GO:0034340	response to type I interferon	4/63	101/18862	0,000362	0,008417	SP100/OAS2/PTPN1/HLA-B
GO:0009150	purine ribonucleotide metabolic process	7/63	408/18862	0,00041	0,009332	PKM/ENO1/PGK1/TPI1/HINT1/TMSB4X/ATP5F1D
GO:0009141	nucleoside triphosphate metabolic process	4/63	109/18862	0,000484	0,010764	PKM/ENO1/TMSB4X/ATP5F1D
GO:0009259	ribonucleotide metabolic process	7/63	425/18862	0,000523	0,01141	PKM/ENO1/PGK1/TPI1/HINT1/TMSB4X/ATP5F1D
GO:0006096	glycolytic process	4/63	114/18862	0,000573	0,01209	PKM/ENO1/PGK1/TPI1
GO:0006757	ATP generation from ADP	4/63	115/18862	0,000592	0,01209	PKM/ENO1/PGK1/TPI1
GO:0007015	actin filament organization	7/63	435/18862	0,000601	0,01209	COTL1/DBN1/CFL1/ACTG1/TMSB4X/BRK1/MARCKS
GO:0019693	ribose phosphate metabolic process	7/63	435/18862	0,000601	0,01209	PKM/ENO1/PGK1/TPI1/HINT1/TMSB4X/ATP5F1D
GO:0006163	purine nucleotide metabolic process	7/63	441/18862	0,000652	0,012862	PKM/ENO1/PGK1/TPI1/HINT1/TMSB4X/ATP5F1D
GO:2000767	positive regulation of cytoplasmic translation	2/63	12/18862	0,000709	0,013491	PKM/EEF2
GO:2001171	positive regulation of ATP biosynthetic process	2/63	12/18862	0,000709	0,013491	ENO1/TMSB4X
GO:0046031	ADP metabolic process	4/63	122/18862	0,000739	0,01381	PKM/ENO1/PGK1/TPI1
GO:0008154	actin polymerization or depolymerization	5/63	215/18862	0,000755	0,013852	COTL1/DBN1/CFL1/TMSB4X/BRK1
GO:0072521	purine-containing compound metabolic process	7/63	460/18862	0,000835	0,015059	PKM/ENO1/PGK1/TPI1/HINT1/TMSB4X/ATP5F1D
GO:0060249	anatomical structure homeostasis	7/63	466/18862	0,0009	0,015968	SP100/ACTB/POTEF/HNRNPA2B1/MUC4/B2M/ACTG1
GO:0032271	regulation of protein polymerization	5/63	225/18862	0,000925	0,016137	COTL1/DBN1/RPS3/TMSB4X/BRK1
GO:0044794	positive regulation by host of viral process	2/63	14/18862	0,000974	0,0167	HSPA8/CFL1
GO:0006165	nucleoside diphosphate phosphorylation	4/63	132/18862	0,000992	0,016738	PKM/ENO1/PGK1/TPI1
GO:0046939	nucleotide phosphorylation	4/63	133/18862	0,00102	0,01694	PKM/ENO1/PGK1/TPI1
GO:0009135	purine nucleoside diphosphate metabolic process	4/63	135/18862	0,001078	0,017355	PKM/ENO1/PGK1/TPI1
GO:0009179	purine ribonucleoside diphosphate metabolic process	4/63	135/18862	0,001078	0,017355	PKM/ENO1/PGK1/TPI1
GO:0009185	ribonucleoside diphosphate metabolic process	4/63	138/18862	0,00117	0,018542	PKM/ENO1/PGK1/TPI1
GO:0032507	maintenance of protein location in cell	3/63	64/18862	0,00128	0,019983	SP100/DBN1/TMSB4X
GO:0043484	regulation of RNA splicing	4/63	145/18862	0,001404	0,021604	FUS/HSPA8/RPS13/HNRNPA2B1
GO:0019885	antigen processing and presentation of endogenous peptide antigen via MHC class I	2/63	17/18862	0,001446	0,021923	B2M/HLA-B
GO:0001894	tissue homeostasis	5/63	260/18862	0,001755	0,026228	ACTB/POTEF/MUC4/B2M/ACTG1
GO:0002483	antigen processing and presentation of endogenous peptide antigen	2/63	19/18862	0,00181	0,026673	B2M/HLA-B
GO:0009132	nucleoside diphosphate metabolic process	4/63	156/18862	0,001836	0,026678	PKM/ENO1/PGK1/TPI1
GO:0051100	negative regulation of binding	4/63	159/18862	0,001968	0,028088	SP100/ACTB/B2M/TMSB4X
GO:2000774	positive regulation of cellular senescence	2/63	20/18862	0,002007	0,028088	HMGA1/B2M
GO:2001242	regulation of intrinsic apoptotic signaling pathway	4/63	160/18862	0,002013	0,028088	ENO1/TPT1/RPS3/PTPN1
GO:0110053	regulation of actin filament organization	5/63	273/18862	0,002171	0,028947	COTL1/DBN1/ACTG1/TMSB4X/BRK1
GO:0150105	protein localization to cell-cell junction	2/63	21/18862	0,002213	0,028947	ACTB/ACTG1
GO:1900118	negative regulation of execution phase of apoptosis	2/63	21/18862	0,002213	0,028947	MTRNR2L8/MTRNR2L1
GO:2000765	regulation of cytoplasmic translation	2/63	21/18862	0,002213	0,028947	PKM/EEF2
GO:2001169	regulation of ATP biosynthetic process	2/63	21/18862	0,002213	0,028947	ENO1/TMSB4X
GO:0030810	positive regulation of nucleotide biosynthetic process	2/63	22/18862	0,002429	0,030229	ENO1/TMSB4X
GO:0032069	regulation of nuclease activity	2/63	22/18862	0,002429	0,030229	OAS2/RPS3
GO:0051220	cytoplasmic sequestering of protein	2/63	22/18862	0,002429	0,030229	DBN1/TMSB4X
GO:1900373	positive regulation of purine nucleotide biosynthetic process	2/63	22/18862	0,002429	0,030229	ENO1/TMSB4X
GO:0030833	regulation of actin filament polymerization	4/63	169/18862	0,002456	0,030229	COTL1/DBN1/TMSB4X/BRK1
GO:0097193	intrinsic apoptotic signaling pathway	5/63	283/18862	0,002537	0,03086	ENO1/TPT1/RPS3/HINT1/PTPN1
GO:0031639	plasminogen activation	2/63	23/18862	0,002655	0,031213	ENO1/PGK1
GO:0042026	protein refolding	2/63	23/18862	0,002655	0,031213	HSPA8/B2M
GO:0090343	positive regulation of cell aging	2/63	23/18862	0,002655	0,031213	HMGA1/B2M
GO:0009152	purine ribonucleotide biosynthetic process	4/63	175/18862	0,002785	0,032381	PKM/ENO1/TMSB4X/ATP5F1D
GO:0071346	cellular response to interferon-gamma	4/63	177/18862	0,002902	0,033362	SP100/OAS2/B2M/HLA-B
GO:0051258	protein polymerization	5/63	294/18862	0,002989	0,033991	COTL1/DBN1/RPS3/TMSB4X/BRK1
GO:0099175	regulation of postsynapse organization	3/63	87/18862	0,003083	0,034683	HSPA8/DBN1/CFL1
GO:0033119	negative regulation of RNA splicing	2/63	25/18862	0,003135	0,034703	RPS13/HNRNPA2B1
GO:0006094	gluconeogenesis	3/63	88/18862	0,003184	0,034703	ENO1/PGK1/TPI1
GO:1903312	negative regulation of mRNA metabolic process	3/63	88/18862	0,003184	0,034703	FUS/HNRNPA2B1/IGF2BP1
GO:0019883	antigen processing and presentation of endogenous antigen	2/63	26/18862	0,003389	0,036299	B2M/HLA-B
GO:0008064	regulation of actin polymerization or depolymerization	4/63	185/18862	0,0034	0,036299	COTL1/DBN1/TMSB4X/BRK1
GO:0030832	regulation of actin filament length	4/63	186/18862	0,003466	0,036314	COTL1/DBN1/TMSB4X/BRK1
GO:0019319	hexose biosynthetic process	3/63	91/18862	0,0035	0,036314	ENO1/PGK1/TPI1
GO:0009260	ribonucleotide biosynthetic process	4/63	188/18862	0,003601	0,036314	PKM/ENO1/TMSB4X/ATP5F1D
GO:0030041	actin filament polymerization	4/63	188/18862	0,003601	0,036314	COTL1/DBN1/TMSB4X/BRK1
GO:0043488	regulation of mRNA stability	4/63	188/18862	0,003601	0,036314	FUS/HSPA8/IGF2BP1/RPS27A
GO:0045185	maintenance of protein location	3/63	92/18862	0,003609	0,036314	SP100/DBN1/TMSB4X
GO:0043254	regulation of protein-containing complex assembly	6/63	446/18862	0,003681	0,036684	COTL1/HSPA8/DBN1/RPS3/TMSB4X/BRK1
GO:2001243	negative regulation of intrinsic apoptotic signaling pathway	3/63	95/18862	0,00395	0,038992	ENO1/TPT1/PTPN1
GO:0046390	ribose phosphate biosynthetic process	4/63	195/18862	0,004101	0,039373	PKM/ENO1/TMSB4X/ATP5F1D
GO:0046364	monosaccharide biosynthetic process	3/63	97/18862	0,004188	0,039373	ENO1/PGK1/TPI1
GO:0001916	positive regulation of T cell mediated cytotoxicity	2/63	29/18862	0,004206	0,039373	B2M/HLA-B
GO:1900117	regulation of execution phase of apoptosis	2/63	29/18862	0,004206	0,039373	MTRNR2L8/MTRNR2L1
GO:0006164	purine nucleotide biosynthetic process	4/63	197/18862	0,004252	0,039373	PKM/ENO1/TMSB4X/ATP5F1D
GO:0016052	carbohydrate catabolic process	4/63	197/18862	0,004252	0,039373	PKM/ENO1/PGK1/TPI1
GO:0034341	response to interferon-gamma	4/63	197/18862	0,004252	0,039373	SP100/OAS2/B2M/HLA-B
GO:0043487	regulation of RNA stability	4/63	198/18862	0,004329	0,039734	FUS/HSPA8/IGF2BP1/RPS27A
GO:0070633	transepithelial transport	2/63	30/18862	0,004497	0,040914	ACTB/ACTG1
GO:0000079	regulation of cyclin-dependent protein serine/threonine kinase activity	3/63	101/18862	0,004689	0,042296	ACTB/CNPPD1/CCND2
GO:0044788	modulation by host of viral process	2/63	31/18862	0,004797	0,042896	HSPA8/CFL1
GO:0071456	cellular response to hypoxia	4/63	206/18862	0,004979	0,044149	ENO1/PGK1/SLC29A1/RPS27A
GO:1903311	regulation of mRNA metabolic process	5/63	334/18862	0,005131	0,044905	FUS/HSPA8/HNRNPA2B1/IGF2BP1/RPS27A
GO:0072522	purine-containing compound biosynthetic process	4/63	208/18862	0,005151	0,044905	PKM/ENO1/TMSB4X/ATP5F1D
GO:1904029	regulation of cyclin-dependent protein kinase activity	3/63	105/18862	0,005225	0,044905	ACTB/CNPPD1/CCND2
GO:0006338	chromatin remodeling	4/63	209/18862	0,005239	0,044905	ACTB/KDM5B/HMGA1/VPS72
GO:0006006	glucose metabolic process	4/63	210/18862	0,005327	0,044905	PKM/ENO1/PGK1/TPI1
GO:0061013	regulation of mRNA catabolic process	4/63	210/18862	0,005327	0,044905	FUS/HSPA8/IGF2BP1/RPS27A
GO:1901222	regulation of NIK/NF-kappaB signaling	3/63	106/18862	0,005365	0,044905	RPS3/LITAF/TMSB4X
GO:0035633	maintenance of blood-brain barrier	2/63	33/18862	0,005423	0,045035	ACTB/ACTG1
GO:0043312	neutrophil degranulation	6/63	485/18862	0,005521	0,045486	PKM/COTL1/HSPA8/B2M/EEF2/HLA-B
GO:0002283	neutrophil activation involved in immune response	6/63	488/18862	0,005686	0,046163	PKM/COTL1/HSPA8/B2M/EEF2/HLA-B
GO:0036294	cellular response to decreased oxygen levels	4/63	214/18862	0,005691	0,046163	ENO1/PGK1/SLC29A1/RPS27A
GO:1902414	protein localization to cell junction	3/63	109/18862	0,005797	0,046654	ACTB/DBN1/ACTG1
GO:0071900	regulation of protein serine/threonine kinase activity	6/63	492/18862	0,005912	0,04722	ACTB/CNPPD1/CCND2/RPS3/PTPN1/RPS27A
GO:0001666	response to hypoxia	5/63	348/18862	0,006088	0,047536	PKM/ENO1/PGK1/SLC29A1/RPS27A
GO:0019058	viral life cycle	5/63	348/18862	0,006088	0,047536	HSPA8/OAS2/RPSA/BSG/RPS27A
GO:2001233	regulation of apoptotic signaling pathway	5/63	348/18862	0,006088	0,047536	SP100/ENO1/TPT1/RPS3/PTPN1
GO:0002446	neutrophil mediated immunity	6/63	499/18862	0,006323	0,048751	PKM/COTL1/HSPA8/B2M/EEF2/HLA-B
GO:0032956	regulation of actin cytoskeleton organization	5/63	352/18862	0,006383	0,048751	COTL1/DBN1/ACTG1/TMSB4X/BRK1
GO:0042119	neutrophil activation	6/63	500/18862	0,006383	0,048751	PKM/COTL1/HSPA8/B2M/EEF2/HLA-B
GO:0032392	DNA geometric change	3/63	114/18862	0,006562	0,049751	HNRNPA2B1/HMGA1/RPS27A

**Table S3:** Target miRNAs of the lncRNAs *MALAT1, MEG3* and *TUG1*

LncRNAs	Number of targets	miRNAs
*MALAT1 - MEG3 - TUG1*	17	hsa-miR-888-5p, hsa-miR-561-5p, hsa-miR-3142, hsa-miR-376b-3p, hsa-miR-320c, hsa-miR-4429, hsa-miR-494-3p, hsa-miR-330-5p, hsa-miR-129-5p, hsa-miR-1245b-5p, hsa-miR-320a, hsa-miR-320b, hsa-miR-5195-3p, hsa-miR-376a-3p, hsa-miR-326, hsa-miR-145-5p, hsa-miR-320d
*MALAT1 - MEG3*	24	hsa-miR-665, hsa-miR-23c, hsa-miR-1252-5p, hsa-miR-23b-3p, hsa-miR-6884-5p, hsa-miR-23a-3p, hsa-miR-143-3p, hsa-miR-6763-5p, hsa-miR-670-5p, hsa-miR-181d-5p, hsa-miR-6088, hsa-miR-181b-5p, hsa-miR-181a-5p, hsa-miR-485-5p, hsa-miR-130a-5p, hsa-miR-3150a-3p, hsa-miR-4770, hsa-miR-4262, hsa-miR-556-3p, hsa-miR-942-5p, hsa-miR-181c-5p, hsa-miR-4640-3p, hsa-miR-216a-5p, hsa-miR-22-3p
*MALAT1 - TUG*	65	hsa-miR-140-5p, hsa-miR-3622a-5p, hsa-miR-3690, hsa-miR-4306, hsa-miR-185-5p, hsa-miR-498, hsa-miR-515-5p, hsa-miR-1323, hsa-miR-548o-3p, hsa-miR-216b-5p, hsa-miR-802, hsa-miR-650, hsa-miR-3163, hsa-miR-545-3p, hsa-miR-3129-5p, hsa-miR-224-3p, hsa-miR-3121-3p, hsa-miR-4766-5p, hsa-miR-4465, hsa-miR-4644, hsa-miR-194-5p, hsa-miR-9-3p, hsa-miR-7853-5p, hsa-miR-655-3p, hsa-miR-3171, hsa-miR-3194-3p, hsa-miR-539-3p, hsa-miR-760, hsa-miR-664b-3p, hsa-miR-105-5p, hsa-miR-199a-3p, hsa-miR-370-3p, hsa-miR-6509-5p, hsa-miR-3612, hsa-miR-6893-3p, hsa-miR-144-3p, hsa-miR-26a-5p, hsa-miR-6805-3p, hsa-miR-141-3p, hsa-miR-506-5p, hsa-miR-576-5p, hsa-miR-582-5p, hsa-miR-5691, hsa-miR-6509-3p, hsa-miR-511-3p, hsa-miR-374c-5p, hsa-miR-199b-3p, hsa-miR-579-3p, hsa-miR-590-5p, hsa-miR-6835-3p, hsa-miR-485-3p, hsa-miR-425-5p, hsa-miR-200a-3p, hsa-miR-26b-5p, hsa-miR-670-3p, hsa-miR-21-5p, hsa-miR-197-3p, hsa-miR-519e-5p, hsa-miR-2355-3p, hsa-miR-552-5p, hsa-miR-142-3p, hsa-miR-1297, hsa-miR-4428, hsa-miR-328-3p, hsa-miR-3179
*MEG3 - TUG1*	12	hsa-miR-361-5p, hsa-miR-542-3p, hsa-miR-195-5p, hsa-miR-497-5p, hsa-miR-424-5p, hsa-miR-15b-5p, hsa-miR-6766-3p, hsa-miR-6838-5p, hsa-miR-4782-3p, hsa-miR-219a-5p, hsa-miR-15a-5p, hsa-miR-16-5p
*MALAT1*	143	hsa-miR-2682-5p, hsa-miR-487a-3p, hsa-miR-30d-5p, hsa-miR-3064-5p, hsa-miR-149-5p, hsa-miR-34b-5p, hsa-miR-378i, hsa-miR-3139, hsa-miR-6738-5p, hsa-miR-374b-3p, hsa-miR-17-5p, hsa-miR-873-5p, hsa-miR-2114-5p, hsa-miR-92b-3p, hsa-miR-374b-5p, hsa-miR-1287-5p, hsa-miR-6807-3p, hsa-miR-384, hsa-miR-589-5p, hsa-miR-154-5p, hsa-miR-2681-5p, hsa-miR-429, hsa-miR-875-5p, hsa-miR-508-3p, hsa-miR-1306-5p, hsa-miR-154-3p, hsa-miR-1270, hsa-miR-449c-5p, hsa-miR-135b-5p, hsa-miR-378c, hsa-miR-892c-3p, hsa-miR-378e, hsa-miR-483-3p, hsa-miR-374a-5p, hsa-miR-146a-5p, hsa-miR-4524a-5p, hsa-miR-3140-3p, hsa-miR-3611, hsa-miR-1179, hsa-miR-452-5p, hsa-miR-532-3p, hsa-miR-514b-5p, hsa-miR-126-5p, hsa-miR-3146, hsa-miR-3605-3p, hsa-miR-676-3p, hsa-miR-1247-5p, hsa-miR-526b-3p, hsa-miR-378d, hsa-miR-101-3p, hsa-miR-651-5p, hsa-miR-422a, hsa-miR-363-3p, hsa-miR-30b-5p, hsa-miR-362-3p, hsa-miR-205-5p, hsa-miR-5194, hsa-miR-6504-5p, hsa-miR-378h, hsa-miR-146b-5p, hsa-miR-378a-3p, hsa-miR-493-5p, hsa-miR-620, hsa-miR-4524b-5p, hsa-miR-2116-3p, hsa-miR-3167, hsa-miR-330-3p, hsa-miR-28-5p, hsa-miR-625-3p, hsa-miR-4756-5p, hsa-miR-150-5p, hsa-miR-1-3p, hsa-miR-361-3p, hsa-miR-217, hsa-miR-3942-5p, hsa-miR-4703-5p, hsa-miR-769-5p, hsa-miR-32-5p, hsa-miR-556-5p, hsa-miR-1321, hsa-miR-329-3p, hsa-miR-409-5p, hsa-miR-224-5p, hsa-miR-30c-5p, hsa-miR-708-5p, hsa-miR-455-5p, hsa-miR-30a-5p, hsa-miR-499a-5p, hsa-miR-30e-5p, hsa-miR-1271-5p, hsa-miR-378b, hsa-miR-4739, hsa-miR-374c-3p, hsa-miR-876-5p, hsa-miR-135a-5p, hsa-miR-93-5p, hsa-miR-519d-3p, hsa-miR-6866-3p, hsa-miR-200c-3p, hsa-miR-96-5p, hsa-miR-200b-3p, hsa-miR-92a-3p, hsa-miR-491-5p, hsa-miR-1278, hsa-miR-6746-3p, hsa-miR-3200-3p, hsa-miR-7153-5p, hsa-miR-206, hsa-miR-136-5p, hsa-miR-188-5p, hsa-miR-20b-5p, hsa-miR-25-3p, hsa-miR-367-3p, hsa-miR-204-5p, hsa-miR-324-3p, hsa-miR-212-5p, hsa-miR-3145-3p, hsa-miR-506-3p, hsa-miR-338-3p, hsa-miR-211-5p, hsa-miR-323b-3p, hsa-miR-944, hsa-miR-2115-3p, hsa-miR-378f, hsa-miR-106b-5p, hsa-miR-1914-3p, hsa-miR-3126-5p, hsa-miR-625-5p, hsa-miR-369-3p, hsa-miR-346, hsa-miR-124-3p, hsa-miR-155-5p, hsa-miR-1913, hsa-miR-513c-5p, hsa-miR-20a-5p, hsa-miR-526b-5p, hsa-miR-106a-5p, hsa-miR-345-3p, hsa-miR-613, hsa-miR-383-5p, hsa-miR-503-5p, hsa-miR-6875-5p, hsa-miR-4676-3p
*MEG3*	16	hsa-miR-10b-5p, hsa-miR-10a-5p, hsa-miR-642a-5p, hsa-miR-376c-3p, hsa-miR-496, hsa-miR-6512-3p, hsa-miR-885-5p, hsa-miR-668-3p, hsa-miR-488-3p, hsa-miR-339-5p, hsa-miR-543, hsa-miR-7-5p, hsa-miR-4424, hsa-miR-605-3p, hsa-miR-1286, hsa-miR-6720-5p
*TUG1*	110	hsa-miR-520f-3p, hsa-miR-1298-5p, hsa-miR-34c-5p, hsa-miR-522-3p, hsa-miR-381-3p, hsa-miR-221-3p, hsa-miR-500b-5p, hsa-miR-449b-5p, hsa-miR-509-3p, hsa-miR-3200-5p, hsa-miR-212-3p, hsa-miR-1224-5p, hsa-miR-654-3p, hsa-miR-3614-5p, hsa-miR-545-5p, hsa-miR-1276, hsa-miR-3681-3p, hsa-miR-1294, hsa-miR-588, hsa-miR-516b-5p, hsa-miR-1251-5p, hsa-miR-9-5p, hsa-miR-216a-3p, hsa-miR-512-3p, hsa-miR-153-3p, hsa-miR-3617-5p, hsa-miR-4640-5p, hsa-miR-31-5p, hsa-miR-2278, hsa-miR-493-3p, hsa-miR-196a-5p, hsa-miR-299-3p, hsa-miR-4677-3p, hsa-miR-580-3p, hsa-miR-3150b-3p, hsa-miR-510-5p, hsa-miR-4726-5p, hsa-miR-371a-5p, hsa-miR-873-3p, hsa-miR-2681-3p, hsa-miR-627-5p, hsa-miR-1277-5p, hsa-miR-5590-3p, hsa-miR-142-5p, hsa-miR-449a, hsa-miR-4761-5p, hsa-miR-128-3p, hsa-miR-138-5p, hsa-miR-380-3p, hsa-miR-7151-5p, hsa-miR-199b-5p, hsa-miR-5688, hsa-miR-514a-3p, hsa-miR-196b-5p, hsa-miR-29c-3p, hsa-miR-34a-5p, hsa-miR-199a-5p, hsa-miR-335-5p, hsa-miR-624-5p, hsa-miR-186-5p, hsa-miR-190a-5p, hsa-miR-524-5p, hsa-miR-5581-3p, hsa-miR-29b-3p, hsa-miR-5579-3p, hsa-miR-377-3p, hsa-miR-6783-3p, hsa-miR-137, hsa-miR-331-3p, hsa-miR-520a-5p, hsa-miR-421, hsa-miR-378g, hsa-miR-641, hsa-miR-151a-3p, hsa-miR-382-5p, hsa-miR-525-5p, hsa-miR-3194-5p, hsa-miR-340-5p, hsa-miR-300, hsa-miR-148a-3p, hsa-miR-495-3p, hsa-miR-409-3p, hsa-miR-499b-5p, hsa-miR-4784, hsa-miR-29a-3p, hsa-miR-1343-3p, hsa-miR-362-5p, hsa-miR-140-3p, hsa-miR-505-3p, hsa-miR-382-3p, hsa-miR-3622b-5p, hsa-miR-4701-5p, hsa-miR-410-3p, hsa-miR-132-3p, hsa-miR-365b-3p, hsa-miR-514a-5p, hsa-miR-27b-3p, hsa-miR-532-5p, hsa-miR-1911-5p, hsa-miR-222-3p, hsa-miR-148b-3p, hsa-miR-520d-5p, hsa-miR-219a-2-3p, hsa-miR-214-5p, hsa-miR-433-3p, hsa-miR-514b-3p, hsa-miR-365a-3p, hsa-miR-27a-3p, hsa-miR-190b, hsa-miR-152-3p

**Table S4:** Target genes (validated in at least two databases) of the miRNAs regulated by the three lncRNAs dysregulated in T1DM patients with recent diagnosis

MicroRNAs	Number of target or common targets	Validated target gene
hsa-miR-129-5p, hsa-miR-320a, hsa-miR-320b, hsa-miR-320c, hsa-miR-320d	1	*KLHL15*
hsa-miR-320a, hsa-miR-320b, hsa-miR-320c, hsa-miR-320d, hsa-miR-4429	3	*PFN1, SYNM, ZNF275*
hsa-miR-320a, hsa-miR-320b, hsa-miR-320c, hsa-miR-320d, hsa-miR-494-3p	1	*SYNCRIP*
hsa-miR-320a, hsa-miR-320b, hsa-miR-320c, hsa-miR-320d	13	*CREBRF, MAX, SLC38A2, TASOR2, ZBTB33, ULK1, TMEM43, PNN, ABHD12, ZNF267, MCL1, GTPBP2, TNRC6C*
hsa-miR-129-5p, hsa-miR-320a, hsa-miR-494-3p	1	*CDK6*
hsa-miR-145-5p, hsa-miR-320a, hsa-miR-320b	1	*PTP4A2*
hsa-miR-145-5p, hsa-miR-320a, hsa-miR-494-3p	1	*MYC*
hsa-miR-320a, hsa-miR-320c, hsa-miR-320d	1	*GNAI1*
hsa-miR-129-5p, hsa-miR-320a	1	*TNRC6B*
hsa-miR-145-5p, hsa-miR-320a	3	*GOLM1, MYO6, IGF1R*
hsa-miR-145-5p, hsa-miR-326	2	*ACTB, SPTLC1*
hsa-miR-145-5p, hsa-miR-494-3p	1	*CFTR*
hsa-miR-320a, hsa-miR-320b	5	*G3BP1, GLUL, FAM234A, RNF103, ATF7IP*
hsa-miR-320a, hsa-miR-320c	4	*AGO1, MT-CO3, MT-ND1, XBP1*
hsa-miR-320a, hsa-miR-330-5p	1	*PKM*
hsa-miR-320a, hsa-miR-494-3p	3	*PTEN, MAPK1, BMI1*
hsa-miR-320b hsa-miR-320c	1	*MDK*
hsa-miR-326, hsa-miR-330-5p	1	*CCND1*
hsa-miR-376a-3p, hsa-miR-376b-3p	2	*HNRNPA0, ACVR1C*
hsa-miR-494-3p, hsa-miR-561-5p	1	*SMIM13*
hsa-miR-129-5p	51	*CLOCK, SLBP, EP300, ANGEL2, FOXP2, GINM1, AKAP10, TMEM51, SOX4, NIFK, RBM26, FMR1, FADS1, RGS16, BMPR2, NPTX1, ABCG1, COL1A1, SPIN4, RNF149, H4C3, UBE2F, OSTF1, MAP3K2, PCDH19, UBA6, TRMT10C, TP53INP1, PNRC1, ZNF703, CAMTA1, ARID3A, KBTBD6, GALNT1, ETV6, BRD3, PARD3, ABCC5, PRRC2B, LONP2, DDX3X, NOTCH1, PRKCD, CFAP20, ZBTB8A, ZNF410, NR2F2, ago/03, ABCB1, CBX6, KLF6*
hsa-miR-145-5p	96	*CD44, ERBB4, DTD1, SERPINE1, ILK, FAM3C, TGFB2, CCN2, NUFIP2, ADD3, ROBO2, TSPAN6, NDRG2, FZD7, EIF4E, HLTF, AP1G1, MIXL1, SMAD2, CBFB, ALPG, MEST, CDKN1A, TPRG1, ARF6, NEDD9, NANOG, RASA1, ADAM17, CTNND1, KIF21A, MAP1LC3B, SERINC5, AKR1B10, RTKN, MYRF, DENND10, TMEM9B, NDUFA4, POU5F1, PPP3CA, MMP14, MDM2, EGFR, KREMEN1, SP1, FSCN1, CLINT1, ESR1, F11R, SWAP70, TPM3, ALDH3A1, CDK4, CCDC43, COL5A1, FLI1, IRS2, APH1A, PODXL, MUC1, NR1D2, C11orf65, ARL6IP5, LYPLA2, NUP43, ABRACL, KLF5, MTMR14, SPTBN1, NIPSNAP1, CPEB4, IRS1, SRGAP1, CDH2, YES1, ABHD17C, JAG1, CEP19, MMP1, STAT1, C11orf58, GMFB, PPM1D, SOX2, CAMK1D, TNFSF13, VGLL4, PIGF, PARP8, KRT7, ANGPT2, KLF4, ZFAND3, MAP2K6, JADE1*
hsa-miR-320ª	150	*CALM3, TMPO, TPD52, SDHC, VCL, SYNGR2, H2BC12, RTN4, SALL1, PRR14L, RAC1, DNAJC14, SRCAP, PRDX3, WARS1, EPB41, IGF2BP3, CLUH, PDSS1, COTL1, USF2, PAPOLA, GXYLT1, KMT2D, MMS22L, ARL9, NT5C3A, SCAF1, ARPP19, DVL3, NIT2, RAB11A, FAM83G, ATP6V1B2, ATXN1, VIM, MT-CO2, SRSF7, HOXA10, ARHGAP17, CANX, CCND2, CDCA3, COX6B1, PRMT5, DCAF11, FOXP1, VKORC1, PDLIM1, ZIC2, MAPK8IP3, ZNF710, C5orf51, TNRC6A, YOD1, ARF3, MT-ND4, CAPNS1, SNRPB, SPEN, CTNNB1, ZNRD2, RBPJ, EAF1, SLC49A4, UBQLN4, EZH2, ZNF451, OAZ1, PRRC2C, TSC22D2, HNRNPUL1, NEO1, CBX5, CALU, MRPS5, ZNF436, VDAC1, TJAP1, PPIA, EIF3F, CSDE1, DIAPH1, GART, CTPS1, WNK1, USP42, USP16, CDS2, SEC24A, RAB14, ASB6, PPIF, TFRC, FBXO28, RANBP6, BCOR, ILF3, CMAS, ARHGEF2, SAMD4B, CRKL, ANP32B, MTDH, HSP90AB1, DNAJB9, ARF1, ARID5B, DCP2, UBAP2L, EPM2AIP1, SRRM2, MRPS18B, CHD1L, FKBP1A, PANK3, EPHA4, OCRL, MAPK1IP1L, SF3B3, NUDT21, SYF2, ITPRIPL2, PBX3, ERC1, VPS45, MT-ND5, RAN, EIF3L, H1-4, SLC2A1, DR1, ESRRG, RPS6KA3, IGF2BP1, LITAF, CYLD, METTL7A, TUBA1B, SF1, RUNX2, MT-CO1, TDP1, POLR2A, ATP1A1, MOCS1, YWHAZ, ANKRD52, RPS27, HUWE1*
hsa-miR-320b	3	*ELOA, BCL9L, ZNF507*
hsa-miR-320c	3	*RARG, SEC14L1, PCDHA2*
hsa-miR-320d	2	*ZNF148, RBFOX2*
hsa-miR-326	10	*ASXL2, THRAP3, ZNF746, RBM20, SMO, POFUT1, CD9, CLU, ABCF2, MAZ*
hsa-miR-330-5p	5	*ITGA5, ANXA6, ZDHHC9, ARL5B, NDEL1*
hsa-miR-376a-3p	6	*ERO1B, IAPP, SRSF11, TTK, KIF5C, SLC16A1*
hsa-miR-494-3p	31	*PPP1CC, CXCR4, CYCS, GLO1, ZEB1, PLEKHA3, FOXJ3, HNRNPA3, BCL2L11, BASP1, AKT1, NPTN, ACACA, PDIA3, KIF2C, ZC3HAV1L, DCBLD2, RAD23B, ITPRID2, SDC1, PTPN14, RHOB, SLC26A3, PROS1, GTF3C4, DSG2, TRIM36, HIF1A, ARHGAP5, ETF1, UHMK1*
hsa-miR-561-5p	5	*HOXA1, RAB18, ALDH9A1, ARID1A, NUP210*

**Table S5:** Significant KEGG and GO pathways in which the target genes of the 17 target miRNAs regulated by lncRNAs *MALAT1, MEG3*, and *TUG1* participate

KEGG pathway ID	Description	p-value	q-value
hsa05200	Pathways in cancer	9,79252E-08	1,15449E-05
hsa04520	Adherens junction	7,39557E-07	4,35949E-05
hsa05214	Glioma	1,6163E-06	6,35179E-05
hsa04510	Focal adhesion	2,99015E-06	8,81308E-05
hsa05219	Bladder cancer	1,8227E-05	0,000389684
hsa04115	p53 signaling pathway	2,04485E-05	0,000389684
hsa05212	Pancreatic cancer	2,32805E-05	0,000389684
hsa05218	Melanoma	2,64429E-05	0,000389684
hsa05220	Chronic myeloid leukemia	3,38851E-05	0,000431434
hsa05215	Prostate cancer	3,65948E-05	0,000431434
hsa05131	Shigellosis	5,29757E-05	0,000520463
hsa05210	Colorectal cancer	5,29757E-05	0,000520463
hsa05213	Endometrial cancer	9,1296E-05	0,000827948
hsa04110	Cell cycle	0,000246889	0,002079062
hsa04330	Notch signaling pathway	0,000312602	0,00245694
hsa04530	Tight junction	0,00035307	0,002601566
hsa05211	Renal cell carcinoma	0,000742822	0,005151459
hsa05216	Thyroid cancer	0,001176281	0,007704297
hsa04350	TGF-beta signaling pathway	0,002643902	0,015585109
hsa05222	Small cell lung cancer	0,002643902	0,015585109
hsa04722	Neurotrophin signaling pathway	0,003063835	0,017200478
hsa04150	mTOR signaling pathway	0,003270942	0,017528495
hsa05100	Bacterial invasion of epithelial cells	0,003742191	0,01918194
hsa05223	Non-small cell lung cancer	0,003961006	0,019457575
hsa04320	Dorso-ventral axis formation	0,004938422	0,02328856
hsa04670	Leukocyte transendothelial migration	0,005708391	0,025884204
hsa04310	Wnt signaling pathway	0,010271941	0,04434721
hsa04930	Type II diabetes mellitus	0,01101112	0,04434721
hsa04360	Axon guidance	0,011141968	0,04434721
hsa04012	ErbB signaling pathway	0,011284781	0,04434721
hsa04144	Endocytosis	0,012202141	0,046405425
hsa04720	Long-term potentiation	0,013885275	0,051156278
hsa04910	Insulin signaling pathway	0,016017596	0,057223949
hsa04010	MAPK signaling pathway	0,019256075	0,066770292
GO biological process	Description	p-value	q-value
GO:0098727	maintenance of cell number	1,18074E-11	2,86585E-08
GO:0031099	regeneration	1,80661E-11	2,86585E-08
GO:0042063	gliogenesis	5,6255E-11	5,35656E-08
GO:0019827	stem cell population maintenance	7,16618E-11	5,35656E-08
GO:0048545	response to steroid hormone	8,44182E-11	5,35656E-08
GO:0048732	gland development	1,60944E-09	7,27887E-07
GO:0006352	DNA-templated transcription, initiation	1,80215E-09	7,27887E-07
GO:0006367	transcription initiation from RNA polymerase II promoter	1,83541E-09	7,27887E-07
GO:0060485	mesenchyme development	3,08643E-09	1,08801E-06
GO:0010001	glial cell differentiation	4,37721E-09	1,38873E-06
GO:0021537	telencephalon development	1,07599E-08	3,10338E-06
GO:0021543	pallium development	1,55432E-08	4,10941E-06
GO:0030900	forebrain development	2,22731E-08	5,43573E-06
GO:0060070	canonical Wnt signaling pathway	2,73224E-08	6,19171E-06
GO:2000045	regulation of G1/S transition of mitotic cell cycle	4,4556E-08	9,09381E-06
GO:0035690	cellular response to drug	4,58613E-08	9,09381E-06
GO:0010718	positive regulation of epithelial to mesenchymal transition	5,52864E-08	1,03179E-05
GO:2000027	regulation of animal organ morphogenesis	5,99345E-08	1,05639E-05
GO:0000082	G1/S transition of mitotic cell cycle	7,21576E-08	1,16484E-05
GO:0030522	intracellular receptor signaling pathway	7,6866E-08	1,16484E-05
GO:0048608	reproductive structure development	7,71021E-08	1,16484E-05
GO:0001655	urogenital system development	8,46856E-08	1,17792E-05
GO:0110110	positive regulation of animal organ morphogenesis	8,74256E-08	1,17792E-05
GO:0061458	reproductive system development	8,91059E-08	1,17792E-05
GO:0048762	mesenchymal cell differentiation	1,28767E-07	1,63412E-05
GO:1902806	regulation of cell cycle G1/S phase transition	1,8518E-07	2,25965E-05
GO:0035019	somatic stem cell population maintenance	2,19396E-07	2,57801E-05
GO:0044843	cell cycle G1/S phase transition	2,28165E-07	2,58529E-05
GO:0050679	positive regulation of epithelial cell proliferation	2,48543E-07	2,71909E-05
GO:0050673	epithelial cell proliferation	3,14393E-07	3,32484E-05
GO:0002065	columnar/cuboidal epithelial cell differentiation	4,75953E-07	4,74957E-05
GO:0007219	Notch signaling pathway	4,79055E-07	4,74957E-05
GO:0042246	tissue regeneration	5,06149E-07	4,86614E-05
GO:0001706	endoderm formation	6,683E-07	6,23609E-05
GO:0021782	glial cell development	7,1618E-07	6,31159E-05
GO:0021987	cerebral cortex development	7,1618E-07	6,31159E-05
GO:0061013	regulation of mRNA catabolic process	7,37716E-07	6,32568E-05
GO:0034248	regulation of cellular amide metabolic process	7,84088E-07	6,54637E-05
GO:0060562	epithelial tube morphogenesis	8,52316E-07	6,93355E-05
GO:0007178	transmembrane receptor protein serine/threonine kinase signaling pathway	8,96969E-07	7,11438E-05
GO:1903829	positive regulation of cellular protein localization	9,4538E-07	7,26509E-05
GO:2000826	regulation of heart morphogenesis	9,88885E-07	7,26509E-05
GO:0071383	cellular response to steroid hormone stimulus	9,91883E-07	7,26509E-05
GO:0050678	regulation of epithelial cell proliferation	1,00757E-06	7,26509E-05
GO:0001837	epithelial to mesenchymal transition	1,08708E-06	7,4976E-05
GO:0072073	kidney epithelium development	1,08708E-06	7,4976E-05
GO:0001704	formation of primary germ layer	1,16514E-06	7,86501E-05
GO:0001822	kidney development	1,21697E-06	8,04372E-05
GO:0002064	epithelial cell development	1,27828E-06	8,27657E-05
GO:0043254	regulation of protein complex assembly	1,30512E-06	8,28131E-05
GO:0030879	mammary gland development	1,40443E-06	8,73677E-05
GO:0060231	mesenchymal to epithelial transition	1,50469E-06	9,18043E-05
GO:2000134	negative regulation of G1/S transition of mitotic cell cycle	1,68932E-06	0,000101124
GO:0030111	regulation of Wnt signaling pathway	1,75837E-06	0,000103308
GO:0060828	regulation of canonical Wnt signaling pathway	1,88723E-06	0,000108863
GO:0007050	cell cycle arrest	1,93182E-06	0,000109445
GO:0071496	cellular response to external stimulus	2,00024E-06	0,000110649
GO:0035270	endocrine system development	2,02282E-06	0,000110649
GO:0010717	regulation of epithelial to mesenchymal transition	2,17553E-06	0,000116986
GO:0007162	negative regulation of cell adhesion	2,21503E-06	0,000117125
GO:0034330	cell junction organization	2,3353E-06	0,00012146
GO:0006913	nucleocytoplasmic transport	2,42344E-06	0,000124011
GO:0001503	ossification	2,48597E-06	0,000125191
GO:0035850	epithelial cell differentiation involved in kidney development	2,60595E-06	0,000129183
GO:0072001	renal system development	2,73256E-06	0,000133376
GO:0051169	nuclear transport	2,79278E-06	0,000134249
GO:1902807	negative regulation of cell cycle G1/S phase transition	2,86986E-06	0,000135633
GO:0003177	pulmonary valve development	2,90707E-06	0,000135633
GO:0035987	endodermal cell differentiation	3,11348E-06	0,000143158
GO:0007492	endoderm development	3,20663E-06	0,000145335
GO:1903311	regulation of mRNA metabolic process	3,49043E-06	0,00015597
GO:0010464	regulation of mesenchymal cell proliferation	3,7703E-06	0,000166136
GO:1901522	positive regulation of transcription from RNA polymerase II promoter involved in cellular response to chemical stimulus	3,92871E-06	0,000170745
GO:0007409	axonogenesis	4,2521E-06	0,000182302
GO:0045637	regulation of myeloid cell differentiation	4,34796E-06	0,000183926
GO:0003151	outflow tract morphogenesis	4,58412E-06	0,000191365
GO:2001243	negative regulation of intrinsic apoptotic signaling pathway	5,06508E-06	0,000208697
GO:0050769	positive regulation of neurogenesis	5,3439E-06	0,000217362
GO:1903706	regulation of hemopoiesis	5,54899E-06	0,000222847
GO:1904031	positive regulation of cyclin-dependent protein kinase activity	5,72977E-06	0,000225263
GO:0051222	positive regulation of protein transport	5,75116E-06	0,000225263
GO:0043401	steroid hormone mediated signaling pathway	5,89309E-06	0,000228007
GO:0030857	negative regulation of epithelial cell differentiation	6,0665E-06	0,000231889
GO:0007369	gastrulation	6,29588E-06	0,000235546
GO:0006402	mRNA catabolic process	6,31067E-06	0,000235546
GO:0006417	regulation of translation	7,31131E-06	0,000269722
GO:0031669	cellular response to nutrient levels	7,99075E-06	0,000291399
GO:1904951	positive regulation of establishment of protein localization	8,19237E-06	0,000295357
GO:0002053	positive regulation of mesenchymal cell proliferation	8,85418E-06	0,000314716
GO:0033002	muscle cell proliferation	8,92774E-06	0,000314716
GO:0090100	positive regulation of transmembrane receptor protein serine/threonine kinase signaling pathway	9,04071E-06	0,000315196
GO:0010810	regulation of cell-substrate adhesion	9,29189E-06	0,000317996
GO:0010812	negative regulation of cell-substrate adhesion	9,51252E-06	0,000317996
GO:0033627	cell adhesion mediated by integrin	9,51252E-06	0,000317996
GO:0055123	digestive system development	9,52194E-06	0,000317996
GO:0001667	ameboidal-type cell migration	9,8759E-06	0,000322505
GO:0034329	cell junction assembly	9,96191E-06	0,000322505
GO:0090092	regulation of transmembrane receptor protein serine/threonine kinase signaling pathway	9,96191E-06	0,000322505
GO:0007223	Wnt signaling pathway, calcium modulating pathway	1,02015E-05	0,000326925
GO:1904029	regulation of cyclin-dependent protein kinase activity	1,08657E-05	0,00034473
GO:0009267	cellular response to starvation	1,1875E-05	0,000369895
GO:0045927	positive regulation of growth	1,18921E-05	0,000369895
GO:0043217	myelin maintenance	1,25106E-05	0,000385356
GO:0061005	cell differentiation involved in kidney development	1,28059E-05	0,000390659
GO:0010975	regulation of neuron projection development	1,32253E-05	0,000395839
GO:0031667	response to nutrient levels	1,32253E-05	0,000395839
GO:0097306	cellular response to alcohol	1,35045E-05	0,000399138
GO:0031589	cell-substrate adhesion	1,35871E-05	0,000399138
GO:0061180	mammary gland epithelium development	1,5292E-05	0,0004451
GO:0034250	positive regulation of cellular amide metabolic process	1,58003E-05	0,000455715
GO:0030099	myeloid cell differentiation	1,65214E-05	0,00047222
GO:0043488	regulation of mRNA stability	1,69467E-05	0,00048005
GO:0031100	animal organ regeneration	1,71325E-05	0,00048102
GO:0060644	mammary gland epithelial cell differentiation	1,74274E-05	0,000485005
GO:0060968	regulation of gene silencing	1,81594E-05	0,000500983
GO:1903034	regulation of response to wounding	1,9234E-05	0,000526056
GO:0048565	digestive tract development	1,97554E-05	0,000535697
GO:0060964	regulation of gene silencing by miRNA	2,00104E-05	0,000538013
GO:0070482	response to oxygen levels	2,16883E-05	0,000578225
GO:0060411	cardiac septum morphogenesis	2,38258E-05	0,000629921
GO:0006401	RNA catabolic process	2,43482E-05	0,000638412
GO:0043487	regulation of RNA stability	2,46384E-05	0,000640725
GO:0032535	regulation of cellular component size	2,62265E-05	0,000675467
GO:0030219	megakaryocyte differentiation	2,64001E-05	0,000675467
GO:0060147	regulation of posttranscriptional gene silencing	2,77715E-05	0,000691086
GO:0060966	regulation of gene silencing by RNA	2,77715E-05	0,000691086
GO:0003197	endocardial cushion development	2,78819E-05	0,000691086
GO:0010463	mesenchymal cell proliferation	2,78819E-05	0,000691086
GO:1902808	positive regulation of cell cycle G1/S phase transition	2,83091E-05	0,000696235
GO:0009612	response to mechanical stimulus	2,85477E-05	0,000696703
GO:0031960	response to corticosteroid	2,90263E-05	0,000702974
GO:0003159	morphogenesis of an endothelium	3,1664E-05	0,000755324
GO:0061154	endothelial tube morphogenesis	3,1664E-05	0,000755324
GO:0003170	heart valve development	3,20092E-05	0,000757862
GO:0010632	regulation of epithelial cell migration	3,23883E-05	0,000760938
GO:0045652	regulation of megakaryocyte differentiation	3,26188E-05	0,000760938
GO:0090287	regulation of cellular response to growth factor stimulus	3,38829E-05	0,000784656
GO:2001242	regulation of intrinsic apoptotic signaling pathway	3,52005E-05	0,000809263
GO:0043281	regulation of cysteine-type endopeptidase activity involved in apoptotic process	3,75124E-05	0,000852154
GO:0033628	regulation of cell adhesion mediated by integrin	3,76033E-05	0,000852154
GO:0048511	rhythmic process	3,87413E-05	0,000871716
GO:0031668	cellular response to extracellular stimulus	3,90211E-05	0,000871828
GO:0042594	response to starvation	3,958E-05	0,000878131
GO:0045165	cell fate commitment	4,28392E-05	0,000943841
GO:0051896	regulation of protein kinase B signaling	4,4159E-05	0,000964241
GO:1901654	response to ketone	4,4373E-05	0,000964241
GO:0000079	regulation of cyclin-dependent protein serine/threonine kinase activity	4,47403E-05	0,000965608
GO:0060135	maternal process involved in female pregnancy	4,56183E-05	0,000977905
GO:1901653	cellular response to peptide	4,6787E-05	0,000993763
GO:0032868	response to insulin	4,69845E-05	0,000993763
GO:0030513	positive regulation of BMP signaling pathway	4,82391E-05	0,001012212
GO:0048145	regulation of fibroblast proliferation	4,84948E-05	0,001012212
GO:0033629	negative regulation of cell adhesion mediated by integrin	5,0291E-05	0,001042843
GO:0003205	cardiac chamber development	5,10644E-05	0,001052003
GO:0032970	regulation of actin filament-based process	5,23138E-05	0,001069101
GO:0046822	regulation of nucleocytoplasmic transport	5,28812E-05	0,001069101
GO:0035148	tube formation	5,29829E-05	0,001069101
GO:0048144	fibroblast proliferation	5,33511E-05	0,001069101
GO:0003283	atrial septum development	5,35792E-05	0,001069101
GO:0071214	cellular response to abiotic stimulus	5,43982E-05	0,001071959
GO:0104004	cellular response to environmental stimulus	5,43982E-05	0,001071959
GO:0009749	response to glucose	5,55043E-05	0,001087005
GO:0071560	cellular response to transforming growth factor beta stimulus	5,62863E-05	0,001095557
GO:0007009	plasma membrane organization	5,74038E-05	0,001100415
GO:0008593	regulation of Notch signaling pathway	5,74038E-05	0,001100415
GO:0060795	cell fate commitment involved in formation of primary germ layer	5,75765E-05	0,001100415
GO:0060541	respiratory system development	5,86417E-05	0,001114063
GO:1901989	positive regulation of cell cycle phase transition	6,22515E-05	0,001175602
GO:1900087	positive regulation of G1/S transition of mitotic cell cycle	6,54273E-05	0,001228265
GO:0003230	cardiac atrium development	6,83034E-05	0,001260129
GO:0110111	negative regulation of animal organ morphogenesis	6,83034E-05	0,001260129
GO:0003206	cardiac chamber morphogenesis	6,84983E-05	0,001260129
GO:0051897	positive regulation of protein kinase B signaling	6,87134E-05	0,001260129
GO:0009746	response to hexose	7,27928E-05	0,001327269
GO:0003231	cardiac ventricle development	7,34864E-05	0,001330309
GO:0003337	mesenchymal to epithelial transition involved in metanephros morphogenesis	7,42232E-05	0,001330309
GO:0048146	positive regulation of fibroblast proliferation	7,45211E-05	0,001330309
GO:0071559	response to transforming growth factor beta	7,46368E-05	0,001330309
GO:0030278	regulation of ossification	7,67641E-05	0,001351982
GO:0034249	negative regulation of cellular amide metabolic process	7,70603E-05	0,001351982
GO:0050821	protein stabilization	7,71311E-05	0,001351982
GO:0072009	nephron epithelium development	7,89373E-05	0,001376038
GO:0031647	regulation of protein stability	8,01636E-05	0,001387976
GO:0035265	organ growth	8,09228E-05	0,001387976
GO:0009743	response to carbohydrate	8,09345E-05	0,001387976
GO:0048771	tissue remodeling	8,16652E-05	0,001392977
GO:0003158	endothelium development	8,44015E-05	0,001412996
GO:0042552	myelination	8,44015E-05	0,001412996
GO:0003179	heart valve morphogenesis	8,46204E-05	0,001412996
GO:0072132	mesenchyme morphogenesis	8,46204E-05	0,001412996
GO:0097150	neuronal stem cell population maintenance	8,56706E-05	0,001423043
GO:0003007	heart morphogenesis	8,96146E-05	0,001480803
GO:1903035	negative regulation of response to wounding	9,19399E-05	0,001511355
GO:1905314	semi-lunar valve development	9,45204E-05	0,001539104
GO:0034284	response to monosaccharide	9,45982E-05	0,001539104
GO:0007272	ensheathment of neurons	9,66741E-05	0,001549047
GO:0008366	axon ensheathment	9,66741E-05	0,001549047
GO:0032355	response to estradiol	9,66741E-05	0,001549047
GO:0051091	positive regulation of DNA-binding transcription factor activity	9,80463E-05	0,00156314
GO:0097193	intrinsic apoptotic signaling pathway	9,91916E-05	0,001566374
GO:0003279	cardiac septum development	9,92742E-05	0,001566374
GO:0050900	leukocyte migration	9,98379E-05	0,001566374
GO:1901992	positive regulation of mitotic cell cycle phase transition	0,000100224	0,001566374
GO:0009755	hormone-mediated signaling pathway	0,000102971	0,00160142
GO:0017148	negative regulation of translation	0,000104794	0,001621812
GO:0001783	B cell apoptotic process	0,000106414	0,001630975
GO:0003181	atrioventricular valve morphogenesis	0,000106414	0,001630975
GO:0046661	male sex differentiation	0,000112423	0,00171479
GO:0050708	regulation of protein secretion	0,000114711	0,001741316
GO:0002011	morphogenesis of an epithelial sheet	0,000121797	0,001840086
GO:2000116	regulation of cysteine-type endopeptidase activity	0,000124201	0,001867513
GO:0008584	male gonad development	0,000125842	0,001883259
GO:0050714	positive regulation of protein secretion	0,00013328	0,001980398
GO:0031333	negative regulation of protein complex assembly	0,000134206	0,001980398
GO:0046546	development of primary male sexual characteristics	0,000134206	0,001980398
GO:0043491	protein kinase B signaling	0,000139123	0,002043452
GO:0003281	ventricular septum development	0,000143111	0,002092348
GO:0002066	columnar/cuboidal epithelial cell development	0,000153237	0,002230114
GO:0008406	gonad development	0,00015572	0,002255596
GO:0048844	artery morphogenesis	0,000157145	0,002255596
GO:0045787	positive regulation of cell cycle	0,00015726	0,002255596
GO:0006979	response to oxidative stress	0,000158646	0,002255596
GO:0003171	atrioventricular valve development	0,000159254	0,002255596
GO:1903959	regulation of anion transmembrane transport	0,000159254	0,002255596
GO:0072006	nephron development	0,000162192	0,002276492
GO:0001657	ureteric bud development	0,000164185	0,002276492
GO:0048709	oligodendrocyte differentiation	0,000164185	0,002276492
GO:0061448	connective tissue development	0,000164789	0,002276492
GO:0007565	female pregnancy	0,00016526	0,002276492
GO:0016049	cell growth	0,000165752	0,002276492
GO:0022604	regulation of cell morphogenesis	0,000165752	0,002276492
GO:0010948	negative regulation of cell cycle process	0,000169637	0,002319815
GO:0043403	skeletal muscle tissue regeneration	0,000170673	0,002323573
GO:0009952	anterior/posterior pattern specification	0,000171377	0,002323573
GO:0044344	cellular response to fibroblast growth factor stimulus	0,000172558	0,002329156
GO:0050657	nucleic acid transport	0,000173991	0,002329156
GO:0050658	RNA transport	0,000173991	0,002329156
GO:0072163	mesonephric epithelium development	0,00017759	0,00235744
GO:0072164	mesonephric tubule development	0,00017759	0,00235744
GO:0051168	nuclear export	0,000183115	0,002420657
GO:1901991	negative regulation of mitotic cell cycle phase transition	0,000186357	0,002442535
GO:0071902	positive regulation of protein serine/threonine kinase activity	0,000187439	0,002442535
GO:0030307	positive regulation of cell growth	0,000188363	0,002442535
GO:0048660	regulation of smooth muscle cell proliferation	0,000188363	0,002442535
GO:0031016	pancreas development	0,00018862	0,002442535
GO:0031102	neuron projection regeneration	0,000190945	0,002462595
GO:0001711	endodermal cell fate commitment	0,00019497	0,002493891
GO:0072283	metanephric renal vesicle morphogenesis	0,00019497	0,002493891
GO:0031641	regulation of myelination	0,00019573	0,002493891
GO:0051236	establishment of RNA localization	0,000202602	0,002571129
GO:0071260	cellular response to mechanical stimulus	0,000206197	0,002597617
GO:0045137	development of primary sexual characteristics	0,000206809	0,002597617
GO:0060840	artery development	0,000207146	0,002597617
GO:0010634	positive regulation of epithelial cell migration	0,000210186	0,00261507
GO:0048659	smooth muscle cell proliferation	0,000210186	0,00261507
GO:0045930	negative regulation of mitotic cell cycle	0,000216903	0,002688094
GO:0071236	cellular response to antibiotic	0,000219835	0,002713829
GO:0030324	lung development	0,000221882	0,002728489
GO:0021762	substantia nigra development	0,000223601	0,002739004
GO:0033598	mammary gland epithelial cell proliferation	0,000230093	0,002793364
GO:0048679	regulation of axon regeneration	0,000230093	0,002793364
GO:0014013	regulation of gliogenesis	0,00023068	0,002793364
GO:0036293	response to decreased oxygen levels	0,00023204	0,002799159
GO:0014065	phosphatidylinositol 3-kinase signaling	0,000233231	0,002802868
GO:0007179	transforming growth factor beta receptor signaling pathway	0,000235158	0,002815353
GO:0001823	mesonephros development	0,000240679	0,002870626
GO:0016579	protein deubiquitination	0,000247732	0,002943674
GO:0060412	ventricular septum morphogenesis	0,000254503	0,002983562
GO:0021542	dentate gyrus development	0,00025579	0,002983562
GO:0033631	cell-cell adhesion mediated by integrin	0,00025579	0,002983562
GO:0060413	atrial septum morphogenesis	0,00025579	0,002983562
GO:1990000	amyloid fibril formation	0,00025579	0,002983562
GO:0032956	regulation of actin cytoskeleton organization	0,000259366	0,003002021
GO:0046824	positive regulation of nucleocytoplasmic transport	0,00026123	0,003002021
GO:0048857	neural nucleus development	0,00026123	0,003002021
GO:0035567	non-canonical Wnt signaling pathway	0,000262104	0,003002021
GO:0071774	response to fibroblast growth factor	0,000262104	0,003002021
GO:0043434	response to peptide hormone	0,000264387	0,003009533
GO:0002791	regulation of peptide secretion	0,000264657	0,003009533
GO:0048730	epidermis morphogenesis	0,000273411	0,003086853
GO:0030323	respiratory tube development	0,000274376	0,003086853
GO:0050796	regulation of insulin secretion	0,000274376	0,003086853
GO:2001233	regulation of apoptotic signaling pathway	0,000275759	0,003091457
GO:0006403	RNA localization	0,000284102	0,003162635
GO:2001234	negative regulation of apoptotic signaling pathway	0,000284102	0,003162635
GO:0031571	mitotic G1 DNA damage checkpoint	0,000288805	0,00317049
GO:0044819	mitotic G1/S transition checkpoint	0,000288805	0,00317049
GO:0046622	positive regulation of organ growth	0,000288805	0,00317049
GO:1904377	positive regulation of protein localization to cell periphery	0,000288805	0,00317049
GO:0051028	mRNA transport	0,000293937	0,003215707
GO:0002793	positive regulation of peptide secretion	0,000301335	0,003285307
GO:0007517	muscle organ development	0,000313011	0,003385113
GO:0000377	RNA splicing, via transesterification reactions with bulged adenosine as nucleophile	0,00031369	0,003385113
GO:0000398	mRNA splicing, via spliceosome	0,00031369	0,003385113
GO:0044783	G1 DNA damage checkpoint	0,00031867	0,003412475
GO:0048736	appendage development	0,000320354	0,003412475
GO:0060173	limb development	0,000320354	0,003412475
GO:0003209	cardiac atrium morphogenesis	0,000322647	0,003412475
GO:1904837	beta-catenin-TCF complex assembly	0,000322647	0,003412475
GO:0097305	response to alcohol	0,000324143	0,003412475
GO:0035196	production of miRNAs involved in gene silencing by miRNA	0,000326326	0,003412475
GO:0003184	pulmonary valve morphogenesis	0,000329133	0,003412475
GO:0035313	wound healing, spreading of epidermal cells	0,000329133	0,003412475
GO:0045725	positive regulation of glycogen biosynthetic process	0,000329133	0,003412475
GO:0061323	cell proliferation involved in heart morphogenesis	0,000329133	0,003412475
GO:2000136	regulation of cell proliferation involved in heart morphogenesis	0,000329133	0,003412475
GO:1901990	regulation of mitotic cell cycle phase transition	0,000336923	0,003481868
GO:0010631	epithelial cell migration	0,000342418	0,003527161
GO:0030330	DNA damage response, signal transduction by p53 class mediator	0,000344647	0,003527454
GO:0030073	insulin secretion	0,00034467	0,003527454
GO:0000375	RNA splicing, via transesterification reactions	0,000345984	0,003529514
GO:0060560	developmental growth involved in morphogenesis	0,000353444	0,003594061
GO:0016311	dephosphorylation	0,000355812	0,003601443
GO:0045765	regulation of angiogenesis	0,000357373	0,003601443
GO:0007389	pattern specification process	0,000357575	0,003601443
GO:0090276	regulation of peptide hormone secretion	0,000361009	0,003624521
GO:0030098	lymphocyte differentiation	0,000366477	0,003654485
GO:0030856	regulation of epithelial cell differentiation	0,000367449	0,003654485
GO:0045216	cell-cell junction organization	0,000367449	0,003654485
GO:0071156	regulation of cell cycle arrest	0,000369342	0,003661834
GO:0071229	cellular response to acid chemical	0,000378003	0,00370024
GO:0044319	wound healing, spreading of cells	0,000378311	0,00370024
GO:0045737	positive regulation of cyclin-dependent protein serine/threonine kinase activity	0,000378311	0,00370024
GO:0090505	epiboly involved in wound healing	0,000378311	0,00370024
GO:0090132	epithelium migration	0,000379048	0,00370024
GO:0030518	intracellular steroid hormone receptor signaling pathway	0,000382129	0,003718875
GO:0032872	regulation of stress-activated MAPK cascade	0,000384982	0,003731933
GO:1904888	cranial skeletal system development	0,000385823	0,003731933
GO:1901988	negative regulation of cell cycle phase transition	0,000410606	0,003920051
GO:0048639	positive regulation of developmental growth	0,000411482	0,003920051
GO:0008038	neuron recognition	0,000413124	0,003920051
GO:0007221	positive regulation of transcription of Notch receptor target	0,000416392	0,003920051
GO:0031998	regulation of fatty acid beta-oxidation	0,000416392	0,003920051
GO:0060749	mammary gland alveolus development	0,000416392	0,003920051
GO:0061377	mammary gland lobule development	0,000416392	0,003920051
GO:0070875	positive regulation of glycogen metabolic process	0,000416392	0,003920051
GO:0072077	renal vesicle morphogenesis	0,000416392	0,003920051
GO:0070302	regulation of stress-activated protein kinase signaling cascade	0,000418896	0,00393196
GO:0046677	response to antibiotic	0,000425404	0,003981266
GO:0090068	positive regulation of cell cycle process	0,000439212	0,004043112
GO:0003176	aortic valve development	0,000440933	0,004043112
GO:0003338	metanephros morphogenesis	0,000440933	0,004043112
GO:0060317	cardiac epithelial to mesenchymal transition	0,000440933	0,004043112
GO:0060674	placenta blood vessel development	0,000440933	0,004043112
GO:0070570	regulation of neuron projection regeneration	0,000440933	0,004043112
GO:0090504	epiboly	0,000440933	0,004043112
GO:0014706	striated muscle tissue development	0,000446646	0,0040837
GO:0070646	protein modification by small protein removal	0,000455593	0,004153529
GO:0001889	liver development	0,000456977	0,004154211
GO:0007548	sex differentiation	0,000461632	0,004171106
GO:0090130	tissue migration	0,000462616	0,004171106
GO:0008347	glial cell migration	0,00046278	0,004171106
GO:0001656	metanephros development	0,00050459	0,004535065
GO:0070227	lymphocyte apoptotic process	0,000507306	0,004539325
GO:0034599	cellular response to oxidative stress	0,000507925	0,004539325
GO:0046620	regulation of organ growth	0,000515738	0,00459562
GO:0032869	cellular response to insulin stimulus	0,000517122	0,00459562
GO:0072087	renal vesicle development	0,000518986	0,004599303
GO:0048568	embryonic organ development	0,000540469	0,004776347
GO:0061008	hepaticobiliary system development	0,000543616	0,004790813
GO:0008543	fibroblast growth factor receptor signaling pathway	0,000550073	0,004820938
GO:0045446	endothelial cell differentiation	0,000550073	0,004820938
GO:0031050	dsRNA processing	0,000575956	0,005006295
GO:0031103	axon regeneration	0,000575956	0,005006295
GO:0070918	production of small RNA involved in gene silencing by RNA	0,000575956	0,005006295
GO:1904375	regulation of protein localization to cell periphery	0,000586259	0,005081925
GO:0051090	regulation of DNA-binding transcription factor activity	0,000607108	0,005237252
GO:0007015	actin filament organization	0,00060748	0,005237252
GO:0072080	nephron tubule development	0,000628824	0,005391962
GO:1901655	cellular response to ketone	0,000628824	0,005391962
GO:0003215	cardiac right ventricle morphogenesis	0,000638353	0,00544403
GO:0032495	response to muramyl dipeptide	0,000638353	0,00544403
GO:1902373	negative regulation of mRNA catabolic process	0,000640044	0,00544403
GO:0030072	peptide hormone secretion	0,000654592	0,005552886
GO:0045666	positive regulation of neuron differentiation	0,000657705	0,005564412
GO:0044706	multi-multicellular organism process	0,000668754	0,005600282
GO:0042326	negative regulation of phosphorylation	0,000668881	0,005600282
GO:0042113	B cell activation	0,000673389	0,005600282
GO:0060993	kidney morphogenesis	0,000675326	0,005600282
GO:0001893	maternal placenta development	0,000676066	0,005600282
GO:0003203	endocardial cushion morphogenesis	0,000676066	0,005600282
GO:0006369	termination of RNA polymerase II transcription	0,000676066	0,005600282
GO:0035909	aorta morphogenesis	0,000676066	0,005600282
GO:0008380	RNA splicing	0,000687383	0,005679204
GO:0010976	positive regulation of neuron projection development	0,000697844	0,005750653
GO:0051098	regulation of binding	0,000699896	0,005752625
GO:0060563	neuroepithelial cell differentiation	0,000709521	0,005816661
GO:0003208	cardiac ventricle morphogenesis	0,000714658	0,005828654
GO:0043627	response to estrogen	0,000714658	0,005828654
GO:0010256	endomembrane system organization	0,000720383	0,00585204
GO:0060538	skeletal muscle organ development	0,000721214	0,00585204
GO:0061326	renal tubule development	0,000724562	0,005864202
GO:0001649	osteoblast differentiation	0,000757642	0,006100811
GO:0007160	cell-matrix adhesion	0,000757642	0,006100811
GO:0060537	muscle tissue development	0,000770039	0,00618494
GO:0051973	positive regulation of telomerase activity	0,000772114	0,006185945
GO:0045747	positive regulation of Notch signaling pathway	0,00078469	0,006255103
GO:0050994	regulation of lipid catabolic process	0,00078469	0,006255103
GO:0072089	stem cell proliferation	0,000797635	0,00631852
GO:0031098	stress-activated protein kinase signaling cascade	0,00079862	0,00631852
GO:0051961	negative regulation of nervous system development	0,00079862	0,00631852
GO:0048638	regulation of developmental growth	0,000827854	0,006533521
GO:0044773	mitotic DNA damage checkpoint	0,000831699	0,006547577
GO:0051403	stress-activated MAPK cascade	0,000835282	0,006559511
GO:0006353	DNA-templated transcription, termination	0,000840851	0,006572888
GO:0042542	response to hydrogen peroxide	0,000843201	0,006572888
GO:0051384	response to glucocorticoid	0,000843201	0,006572888
GO:0048863	stem cell differentiation	0,000856736	0,006662026
GO:2000142	regulation of DNA-templated transcription, initiation	0,000877988	0,006810595
GO:0003183	mitral valve morphogenesis	0,000909455	0,007003312
GO:0007440	foregut morphogenesis	0,000909455	0,007003312
GO:0098734	macromolecule depalmitoylation	0,000909455	0,007003312
GO:1901987	regulation of cell cycle phase transition	0,000922053	0,007083136
GO:1904018	positive regulation of vasculature development	0,000927834	0,007110324
GO:0002052	positive regulation of neuroblast proliferation	0,000933229	0,007134435
GO:0031346	positive regulation of cell projection organization	0,000947534	0,007226387
GO:0001708	cell fate specification	0,0009512	0,007235945
GO:0006977	DNA damage response, signal transduction by p53 class mediator resulting in cell cycle arrest	0,000953349	0,007235945
GO:0048678	response to axon injury	0,000984004	0,007446319
GO:0050773	regulation of dendrite development	0,00098576	0,007446319
GO:0000302	response to reactive oxygen species	0,001004346	0,007561612
GO:0007043	cell-cell junction assembly	0,001008173	0,007561612
GO:0014066	regulation of phosphatidylinositol 3-kinase signaling	0,001008173	0,007561612
GO:0045766	positive regulation of angiogenesis	0,001024828	0,007668401
GO:0072431	signal transduction involved in mitotic G1 DNA damage checkpoint	0,00104748	0,007801097
GO:1902400	intracellular signal transduction involved in G1 DNA damage checkpoint	0,00104748	0,007801097
GO:0071157	negative regulation of cell cycle arrest	0,001111662	0,008240405
GO:0072215	regulation of metanephros development	0,001111662	0,008240405
GO:0048713	regulation of oligodendrocyte differentiation	0,001121672	0,008275938
GO:2000648	positive regulation of stem cell proliferation	0,001121672	0,008275938
GO:0050768	negative regulation of neurogenesis	0,001140737	0,00836906
GO:1901342	regulation of vasculature development	0,001145214	0,00836906
GO:1903312	negative regulation of mRNA metabolic process	0,001145666	0,00836906
GO:0001890	placenta development	0,001147492	0,00836906
GO:0035904	aorta development	0,001148583	0,00836906
GO:0006611	protein export from nucleus	0,001155397	0,00836906
GO:0008361	regulation of cell size	0,001155397	0,00836906
GO:0051099	positive regulation of binding	0,001155397	0,00836906
GO:0045727	positive regulation of translation	0,001193932	0,008628487
GO:0001666	response to hypoxia	0,00119949	0,008648954
GO:1905330	regulation of morphogenesis of an epithelium	0,001208661	0,008694273
GO:0007623	circadian rhythm	0,001211256	0,008694273
GO:0002328	pro-B cell differentiation	0,001231641	0,008761307
GO:0003174	mitral valve development	0,001231641	0,008761307
GO:0033632	regulation of cell-cell adhesion mediated by integrin	0,001231641	0,008761307
GO:0061314	Notch signaling involved in heart development	0,001231641	0,008761307
GO:0072413	signal transduction involved in mitotic cell cycle checkpoint	0,001256995	0,008793825
GO:1902369	negative regulation of RNA catabolic process	0,001256995	0,008793825
GO:1902402	signal transduction involved in mitotic DNA damage checkpoint	0,001256995	0,008793825
GO:1902403	signal transduction involved in mitotic DNA integrity checkpoint	0,001256995	0,008793825
GO:0098760	response to interleukin-7	0,001260737	0,008793825
GO:0098761	cellular response to interleukin-7	0,001260737	0,008793825
GO:2000008	regulation of protein localization to cell surface	0,001260737	0,008793825
GO:0008154	actin polymerization or depolymerization	0,001262012	0,008793825
GO:0022408	negative regulation of cell-cell adhesion	0,00126393	0,008793825
GO:0048015	phosphatidylinositol-mediated signaling	0,00126393	0,008793825
GO:0010770	positive regulation of cell morphogenesis involved in differentiation	0,00126689	0,008795129
GO:0031331	positive regulation of cellular catabolic process	0,001273472	0,008821521
GO:0031334	positive regulation of protein complex assembly	0,001279632	0,008844885
GO:0071887	leukocyte apoptotic process	0,001310768	0,00901461
GO:0001654	eye development	0,00131189	0,00901461
GO:0003272	endocardial cushion formation	0,001312711	0,00901461
GO:0030041	actin filament polymerization	0,00132126	0,009037584
GO:0021766	hippocampus development	0,001327451	0,009037584
GO:0034109	homotypic cell-cell adhesion	0,001327451	0,009037584
GO:0048708	astrocyte differentiation	0,001327451	0,009037584
GO:0051047	positive regulation of secretion	0,001348483	0,009161112
GO:0021953	central nervous system neuron differentiation	0,00138071	0,009360007
GO:0051052	regulation of DNA metabolic process	0,001385184	0,009370319
GO:0010769	regulation of cell morphogenesis involved in differentiation	0,001392806	0,009401831
GO:0015931	nucleobase-containing compound transport	0,001417176	0,009546027
GO:0030279	negative regulation of ossification	0,001426412	0,009587878
GO:0048017	inositol lipid-mediated signaling	0,001442337	0,009674427
GO:0042110	T cell activation	0,001447356	0,009687609
GO:0150063	visual system development	0,001475535	0,009855432
GO:0044774	mitotic DNA integrity checkpoint	0,001481765	0,009867945
GO:0030183	B cell differentiation	0,001483629	0,009867945
GO:1903532	positive regulation of secretion by cell	0,001498696	0,009947299
GO:0006970	response to osmotic stress	0,001531036	0,010059772
GO:0002068	glandular epithelial cell development	0,001537837	0,010059772
GO:0030325	adrenal gland development	0,001537837	0,010059772
GO:0031069	hair follicle morphogenesis	0,001537837	0,010059772
GO:0048103	somatic stem cell division	0,001537837	0,010059772
GO:0072202	cell differentiation involved in metanephros development	0,001537837	0,010059772
GO:0072273	metanephric nephron morphogenesis	0,001537837	0,010059772
GO:0042476	odontogenesis	0,001564227	0,010190383
GO:0072175	epithelial tube formation	0,001564227	0,010190383
GO:0010907	positive regulation of glucose metabolic process	0,001576514	0,010249382
GO:0021889	olfactory bulb interneuron differentiation	0,001617447	0,010430006
GO:0060081	membrane hyperpolarization	0,001617447	0,010430006
GO:0060213	positive regulation of nuclear-transcribed mRNA poly(A) tail shortening	0,001617447	0,010430006
GO:0072697	protein localization to cell cortex	0,001617447	0,010430006
GO:0043154	negative regulation of cysteine-type endopeptidase activity involved in apoptotic process	0,001641544	0,010563924
GO:0042770	signal transduction in response to DNA damage	0,001648303	0,010585947
GO:2000278	regulation of DNA biosynthetic process	0,001669998	0,01067781
GO:0022407	regulation of cell-cell adhesion	0,001672703	0,01067781
GO:0045785	positive regulation of cell adhesion	0,001672703	0,01067781
GO:0007519	skeletal muscle tissue development	0,001687146	0,010748377
GO:0048872	homeostasis of number of cells	0,001701814	0,010812684
GO:0048880	sensory system development	0,001704056	0,010812684
GO:0050920	regulation of chemotaxis	0,001734969	0,010971265
GO:0018210	peptidyl-threonine modification	0,001735964	0,010971265
GO:0001953	negative regulation of cell-matrix adhesion	0,001754529	0,011022721
GO:0008631	intrinsic apoptotic signaling pathway in response to oxidative stress	0,001754529	0,011022721
GO:0032459	regulation of protein oligomerization	0,001754529	0,011022721
GO:0014031	mesenchymal cell development	0,001758158	0,011023689
GO:0034394	protein localization to cell surface	0,001770668	0,011080229
GO:0001701	in utero embryonic development	0,001803463	0,011263235
GO:0007599	hemostasis	0,001817255	0,011327077
GO:0050921	positive regulation of chemotaxis	0,001827321	0,01136748
GO:0016569	covalent chromatin modification	0,001859655	0,011545986
GO:0043583	ear development	0,001873576	0,0116097
GO:0048013	ephrin receptor signaling pathway	0,001881103	0,01163362
GO:0045931	positive regulation of mitotic cell cycle	0,001936064	0,011950227
GO:0042987	amyloid precursor protein catabolic process	0,00194684	0,011993409
GO:0002690	positive regulation of leukocyte chemotaxis	0,00201061	0,012362256
GO:0032530	regulation of microvillus organization	0,002071035	0,012587413
GO:0048681	negative regulation of axon regeneration	0,002071035	0,012587413
GO:0060576	intestinal epithelial cell development	0,002071035	0,012587413
GO:0061042	vascular wound healing	0,002071035	0,012587413
GO:0061478	response to platelet aggregation inhibitor	0,002071035	0,012587413
GO:1903960	negative regulation of anion transmembrane transport	0,002071035	0,012587413
GO:2000177	regulation of neural precursor cell proliferation	0,00214691	0,01299278
GO:0031018	endocrine pancreas development	0,002154112	0,01299278
GO:0048255	mRNA stabilization	0,002154112	0,01299278
GO:0070316	regulation of G0 to G1 transition	0,002154112	0,01299278
GO:0021700	developmental maturation	0,002196368	0,013222517
GO:0033157	regulation of intracellular protein transport	0,002344901	0,01406334
GO:0045665	negative regulation of neuron differentiation	0,002344901	0,01406334
GO:0042634	regulation of hair cycle	0,002372106	0,014148939
GO:0060260	regulation of transcription initiation from RNA polymerase II promoter	0,002372106	0,014148939
GO:0002685	regulation of leukocyte migration	0,002374396	0,014148939
GO:0002067	glandular epithelial cell differentiation	0,002377012	0,014148939
GO:0007611	learning or memory	0,002413815	0,014301341
GO:0034614	cellular response to reactive oxygen species	0,002416176	0,014301341
GO:1990138	neuron projection extension	0,002416176	0,014301341
GO:0003002	regionalization	0,002427923	0,014301341
GO:0048662	negative regulation of smooth muscle cell proliferation	0,002429662	0,014301341
GO:0060038	cardiac muscle cell proliferation	0,002429662	0,014301341
GO:0030901	midbrain development	0,002440836	0,014340506
GO:0009411	response to UV	0,002459373	0,014422708
GO:0034332	adherens junction organization	0,002579789	0,015067604
GO:1902904	negative regulation of supramolecular fiber organization	0,002579789	0,015067604
GO:0006470	protein dephosphorylation	0,002594707	0,015067604
GO:0071375	cellular response to peptide hormone stimulus	0,002594707	0,015067604
GO:0060211	regulation of nuclear-transcribed mRNA poly(A) tail shortening	0,002596232	0,015067604
GO:0051054	positive regulation of DNA metabolic process	0,002614518	0,015067604
GO:0045023	G0 to G1 transition	0,002616213	0,015067604
GO:0061383	trabecula morphogenesis	0,002616213	0,015067604
GO:0070849	response to epidermal growth factor	0,002616213	0,015067604
GO:0006661	phosphatidylinositol biosynthetic process	0,002619752	0,015067604
GO:0016570	histone modification	0,002621583	0,015067604
GO:0035966	response to topologically incorrect protein	0,002670901	0,015323297
GO:0005979	regulation of glycogen biosynthetic process	0,002707897	0,015462601
GO:0010962	regulation of glucan biosynthetic process	0,002707897	0,015462601
GO:0010594	regulation of endothelial cell migration	0,002709803	0,015462601
GO:1901222	regulation of NIK/NF-kappaB signaling	0,002763258	0,015719883
GO:2000117	negative regulation of cysteine-type endopeptidase activity	0,002764801	0,015719883
GO:0009896	positive regulation of catabolic process	0,002820538	0,015982713
GO:0042698	ovulation cycle	0,002821103	0,015982713
GO:0071322	cellular response to carbohydrate stimulus	0,002834625	0,016030698
GO:0007595	lactation	0,002872386	0,016129243
GO:0030850	prostate gland development	0,002872386	0,016129243
GO:0051972	regulation of telomerase activity	0,002872386	0,016129243
GO:0060324	face development	0,002872386	0,016129243
GO:0051146	striated muscle cell differentiation	0,002915611	0,016343036
GO:0032092	positive regulation of protein binding	0,002938659	0,016443182
GO:0052548	regulation of endopeptidase activity	0,002964861	0,016556196
GO:0000077	DNA damage checkpoint	0,002969294	0,016556196
GO:0009615	response to virus	0,003002044	0,016709438
GO:0072091	regulation of stem cell proliferation	0,003033648	0,016855774
GO:0001952	regulation of cell-matrix adhesion	0,003068633	0,0169659
GO:0046320	regulation of fatty acid oxidation	0,003074858	0,0169659
GO:0048384	retinoic acid receptor signaling pathway	0,003074858	0,0169659
GO:0097421	liver regeneration	0,003074858	0,0169659
GO:0016358	dendrite development	0,0031196	0,017182887
GO:0048546	digestive tract morphogenesis	0,003146205	0,017299392
GO:0050890	cognition	0,003194486	0,017365525
GO:0034111	negative regulation of homotypic cell-cell adhesion	0,003196547	0,017365525
GO:0042159	lipoprotein catabolic process	0,003196547	0,017365525
GO:0045898	regulation of RNA polymerase II transcriptional preinitiation complex assembly	0,003196547	0,017365525
GO:0070571	negative regulation of neuron projection regeneration	0,003196547	0,017365525
GO:0072075	metanephric mesenchyme development	0,003196547	0,017365525
GO:0072148	epithelial cell fate commitment	0,003196547	0,017365525
GO:0048588	developmental cell growth	0,003229521	0,017462157
GO:0006342	chromatin silencing	0,003230847	0,017462157
GO:0017015	regulation of transforming growth factor beta receptor signaling pathway	0,003230847	0,017462157
GO:0120032	regulation of plasma membrane bounded cell projection assembly	0,003244193	0,01750447
GO:0061351	neural precursor cell proliferation	0,003253764	0,017526306
GO:0032984	protein-containing complex disassembly	0,003270787	0,017565037
GO:0001933	negative regulation of protein phosphorylation	0,003272027	0,017565037
GO:0044070	regulation of anion transport	0,003311374	0,017716306
GO:1903076	regulation of protein localization to plasma membrane	0,003311374	0,017716306
GO:0046883	regulation of hormone secretion	0,003353912	0,017913681
GO:0030968	endoplasmic reticulum unfolded protein response	0,003399646	0,018028503
GO:0048675	axon extension	0,003399646	0,018028503
GO:0035107	appendage morphogenesis	0,003403822	0,018028503
GO:0035108	limb morphogenesis	0,003403822	0,018028503
GO:0061041	regulation of wound healing	0,003403822	0,018028503
GO:0018205	peptidyl-lysine modification	0,003428847	0,018130781
GO:0043489	RNA stabilization	0,003438343	0,01815074
GO:0032024	positive regulation of insulin secretion	0,003494335	0,018379979
GO:0061035	regulation of cartilage development	0,003494335	0,018379979
GO:0090277	positive regulation of peptide hormone secretion	0,003510735	0,018379979
GO:1990823	response to leukemia inhibitory factor	0,003510735	0,018379979
GO:1990830	cellular response to leukemia inhibitory factor	0,003510735	0,018379979
GO:0060491	regulation of cell projection assembly	0,003518138	0,018388397
GO:0032102	negative regulation of response to external stimulus	0,003561839	0,01858619
GO:1903844	regulation of cellular response to transforming growth factor beta stimulus	0,003575206	0,018625309
GO:0090090	negative regulation of canonical Wnt signaling pathway	0,003661832	0,019045319
GO:0043255	regulation of carbohydrate biosynthetic process	0,003719104	0,019247954
GO:0048754	branching morphogenesis of an epithelial tube	0,003720236	0,019247954
GO:0000281	mitotic cytokinesis	0,003743261	0,019247954
GO:0045685	regulation of glial cell differentiation	0,003743261	0,019247954
GO:0060350	endochondral bone morphogenesis	0,003743261	0,019247954
GO:0072401	signal transduction involved in DNA integrity checkpoint	0,003743261	0,019247954
GO:0072422	signal transduction involved in DNA damage checkpoint	0,003743261	0,019247954
GO:0030336	negative regulation of cell migration	0,003762492	0,019315536
GO:0008064	regulation of actin polymerization or depolymerization	0,003810142	0,019493186
GO:0048705	skeletal system morphogenesis	0,003827109	0,019493186
GO:0002070	epithelial cell maturation	0,003875183	0,019493186
GO:0003222	ventricular trabecula myocardium morphogenesis	0,003875183	0,019493186
GO:0030033	microvillus assembly	0,003875183	0,019493186
GO:0034975	protein folding in endoplasmic reticulum	0,003875183	0,019493186
GO:0048820	hair follicle maturation	0,003875183	0,019493186
GO:0060965	negative regulation of gene silencing by miRNA	0,003875183	0,019493186
GO:0097091	synaptic vesicle clustering	0,003875183	0,019493186
GO:0099170	postsynaptic modulation of chemical synaptic transmission	0,003875183	0,019493186
GO:1902001	fatty acid transmembrane transport	0,003875183	0,019493186
GO:1902004	positive regulation of amyloid-beta formation	0,003875183	0,019493186
GO:0051216	cartilage development	0,00388175	0,019493186
GO:0046685	response to arsenic-containing substance	0,003907692	0,019493186
GO:0051385	response to mineralocorticoid	0,003907692	0,019493186
GO:0061311	cell surface receptor signaling pathway involved in heart development	0,003907692	0,019493186
GO:0061384	heart trabecula morphogenesis	0,003907692	0,019493186
GO:0071353	cellular response to interleukin-4	0,003907692	0,019493186
GO:0001838	embryonic epithelial tube formation	0,003947331	0,019660013
GO:0030217	T cell differentiation	0,003956678	0,019675677
GO:0030832	regulation of actin filament length	0,00396317	0,019677119
GO:0007596	blood coagulation	0,003975407	0,019707032
GO:0014015	positive regulation of gliogenesis	0,004005102	0,019792388
GO:0072395	signal transduction involved in cell cycle checkpoint	0,004005102	0,019792388
GO:0032330	regulation of chondrocyte differentiation	0,004080262	0,020070028
GO:0060421	positive regulation of heart growth	0,004080262	0,020070028
GO:2000179	positive regulation of neural precursor cell proliferation	0,004080262	0,020070028
GO:0042593	glucose homeostasis	0,004089752	0,020085569
GO:0030326	embryonic limb morphogenesis	0,004144258	0,020290436
GO:0035113	embryonic appendage morphogenesis	0,004144258	0,020290436
GO:0043542	endothelial cell migration	0,004174403	0,020406539
GO:0033500	carbohydrate homeostasis	0,004226399	0,020628935
GO:0018107	peptidyl-threonine phosphorylation	0,00434867	0,021193131
GO:0036003	positive regulation of transcription from RNA polymerase II promoter in response to stress	0,004376193	0,021229432
GO:1901186	positive regulation of ERBB signaling pathway	0,004376193	0,021229432
GO:1902692	regulation of neuroblast proliferation	0,004376193	0,021229432
GO:0009895	negative regulation of catabolic process	0,004537015	0,021975996
GO:0061045	negative regulation of wound healing	0,004569115	0,022013938
GO:0072088	nephron epithelium morphogenesis	0,004569115	0,022013938
GO:0007411	axon guidance	0,004572381	0,022013938
GO:0007254	JNK cascade	0,004633211	0,022013938
GO:2001020	regulation of response to DNA damage stimulus	0,004633211	0,022013938
GO:0003198	epithelial to mesenchymal transition involved in endocardial cushion formation	0,004635051	0,022013938
GO:0032460	negative regulation of protein oligomerization	0,004635051	0,022013938
GO:0033599	regulation of mammary gland epithelial cell proliferation	0,004635051	0,022013938
GO:0035988	chondrocyte proliferation	0,004635051	0,022013938
GO:0061318	renal filtration cell differentiation	0,004635051	0,022013938
GO:0072112	glomerular visceral epithelial cell differentiation	0,004635051	0,022013938
GO:1905331	negative regulation of morphogenesis of an epithelium	0,004635051	0,022013938
GO:2000811	negative regulation of anoikis	0,004635051	0,022013938
GO:0050817	coagulation	0,004673977	0,02216563
GO:0097485	neuron projection guidance	0,004711634	0,022310866
GO:0019216	regulation of lipid metabolic process	0,004725819	0,022344686
GO:0002576	platelet degranulation	0,004780693	0,022503528
GO:0002687	positive regulation of leukocyte migration	0,004780693	0,022503528
GO:0034763	negative regulation of transmembrane transport	0,004780693	0,022503528
GO:0001101	response to acid chemical	0,004799564	0,022540439
GO:0046328	regulation of JNK cascade	0,004802744	0,022540439
GO:0034205	amyloid-beta formation	0,00488112	0,022773522
GO:0043276	anoikis	0,00488112	0,022773522
GO:0070670	response to interleukin-4	0,00488112	0,022773522
GO:0071402	cellular response to lipoprotein particle stimulus	0,00488112	0,022773522
GO:2000379	positive regulation of reactive oxygen species metabolic process	0,004905145	0,022852008
GO:0010721	negative regulation of cell development	0,004927881	0,022924267
GO:0001764	neuron migration	0,005012878	0,023251486
GO:0031570	DNA integrity checkpoint	0,005012878	0,023251486
GO:0046879	hormone secretion	0,005074699	0,023503868
GO:0009314	response to radiation	0,005115271	0,023657247
GO:0043543	protein acylation	0,005125152	0,02366844
GO:0043393	regulation of protein binding	0,005136766	0,023687596
GO:0034766	negative regulation of ion transmembrane transport	0,005173024	0,023724124
GO:1905475	regulation of protein localization to membrane	0,005175294	0,023724124
GO:0010827	regulation of glucose transmembrane transport	0,005189554	0,023724124
GO:0021954	central nervous system neuron development	0,005189554	0,023724124
GO:0033143	regulation of intracellular steroid hormone receptor signaling pathway	0,005189554	0,023724124
GO:0072028	nephron morphogenesis	0,005189554	0,023724124
GO:0003229	ventricular cardiac muscle tissue development	0,005197255	0,023725146
GO:0048871	multicellular organismal homeostasis	0,005328893	0,024291112
GO:0009416	response to light stimulus	0,005362205	0,024372925
GO:0043010	camera-type eye development	0,005362205	0,024372925
GO:0021955	central nervous system neuron axonogenesis	0,005423707	0,024546969
GO:0070873	regulation of glycogen metabolic process	0,005423707	0,024546969
GO:1902991	regulation of amyloid precursor protein catabolic process	0,005423707	0,024546969
GO:0001558	regulation of cell growth	0,005447365	0,024585801
GO:0008630	intrinsic apoptotic signaling pathway in response to DNA damage	0,005451744	0,024585801
GO:0002902	regulation of B cell apoptotic process	0,005478783	0,024585801
GO:0045683	negative regulation of epidermis development	0,005478783	0,024585801
GO:0072311	glomerular epithelial cell differentiation	0,005478783	0,024585801
GO:1900153	positive regulation of nuclear-transcribed mRNA catabolic process, deadenylation-dependent decay	0,005478783	0,024585801
GO:0007584	response to nutrient	0,005495854	0,02462757
GO:0052547	regulation of peptidase activity	0,005595574	0,024907708
GO:2000146	negative regulation of cell motility	0,005611919	0,024907708
GO:0022029	telencephalon cell migration	0,005613325	0,024907708
GO:0060688	regulation of morphogenesis of a branching structure	0,005613325	0,024907708
GO:0090183	regulation of kidney development	0,005613325	0,024907708
GO:0098586	cellular response to virus	0,005613325	0,024907708
GO:1903078	positive regulation of protein localization to plasma membrane	0,005613325	0,024907708
GO:0021915	neural tube development	0,005663306	0,02509439
GO:0001841	neural tube formation	0,005741569	0,025340328
GO:0043280	positive regulation of cysteine-type endopeptidase activity involved in apoptotic process	0,005742771	0,025340328
GO:0045995	regulation of embryonic development	0,005742771	0,025340328
GO:0051271	negative regulation of cellular component movement	0,005766105	0,025407953
GO:0035967	cellular response to topologically incorrect protein	0,005894005	0,025935515
GO:0042692	muscle cell differentiation	0,005907378	0,025958358
GO:0001935	endothelial cell proliferation	0,00598813	0,026276804
GO:0071333	cellular response to glucose stimulus	0,006004817	0,026278827
GO:0071392	cellular response to estradiol stimulus	0,006005157	0,026278827
GO:0060043	regulation of cardiac muscle cell proliferation	0,006052348	0,026436675
GO:1902903	regulation of supramolecular fiber organization	0,006057893	0,026436675
GO:0043903	regulation of symbiosis, encompassing mutualism through parasitism	0,006071327	0,026458907
GO:0030833	regulation of actin filament polymerization	0,006131888	0,026686173
GO:0014032	neural crest cell development	0,006232975	0,027014935
GO:0014855	striated muscle cell proliferation	0,006232975	0,027014935
GO:2001021	negative regulation of response to DNA damage stimulus	0,006232975	0,027014935
GO:0043062	extracellular structure organization	0,006256399	0,02701625
GO:0030260	entry into host cell	0,006275855	0,02701625
GO:0044409	entry into host	0,006275855	0,02701625
GO:0051806	entry into cell of other organism involved in symbiotic interaction	0,006275855	0,02701625
GO:0051828	entry into other organism involved in symbiotic interaction	0,006275855	0,02701625
GO:0048524	positive regulation of viral process	0,006355606	0,027146312
GO:0051170	import into nucleus	0,006377098	0,027146312
GO:0099173	postsynapse organization	0,006377098	0,027146312
GO:0032386	regulation of intracellular transport	0,006400265	0,027146312
GO:0060149	negative regulation of posttranscriptional gene silencing	0,006408745	0,027146312
GO:0060438	trachea development	0,006408745	0,027146312
GO:0060716	labyrinthine layer blood vessel development	0,006408745	0,027146312
GO:0060967	negative regulation of gene silencing by RNA	0,006408745	0,027146312
GO:0072074	kidney mesenchyme development	0,006408745	0,027146312
GO:0090201	negative regulation of release of cytochrome c from mitochondria	0,006408745	0,027146312
GO:1903798	regulation of production of miRNAs involved in gene silencing by miRNA	0,006408745	0,027146312
GO:2000010	positive regulation of protein localization to cell surface	0,006408745	0,027146312
GO:0048562	embryonic organ morphogenesis	0,00648053	0,027413777
GO:0030520	intracellular estrogen receptor signaling pathway	0,006514968	0,027486159
GO:0098930	axonal transport	0,006514968	0,027486159
GO:0006405	RNA export from nucleus	0,006556078	0,027586232
GO:0071331	cellular response to hexose stimulus	0,006556078	0,027586232
GO:0034644	cellular response to UV	0,00661244	0,027662974
GO:0071158	positive regulation of cell cycle arrest	0,00661244	0,027662974
GO:0000289	nuclear-transcribed mRNA poly(A) tail shortening	0,006626632	0,027662974
GO:0032885	regulation of polysaccharide biosynthetic process	0,006626632	0,027662974
GO:0042307	positive regulation of protein import into nucleus	0,006626632	0,027662974
GO:1900026	positive regulation of substrate adhesion-dependent cell spreading	0,006626632	0,027662974
GO:0009914	hormone transport	0,006646238	0,027708363
GO:0033673	negative regulation of kinase activity	0,006752384	0,028113946
GO:0045814	negative regulation of gene expression, epigenetic	0,006845677	0,028427765
GO:0071326	cellular response to monosaccharide stimulus	0,006845677	0,028427765
GO:0007093	mitotic cell cycle checkpoint	0,006890083	0,028567039
GO:2000377	regulation of reactive oxygen species metabolic process	0,006897224	0,028567039
GO:0001658	branching involved in ureteric bud morphogenesis	0,007001817	0,028875763
GO:0021885	forebrain cell migration	0,007001817	0,028875763
GO:0070527	platelet aggregation	0,007001817	0,028875763
GO:0045913	positive regulation of carbohydrate metabolic process	0,007008394	0,028875763
GO:0021761	limbic system development	0,00701727	0,028875763
GO:0001763	morphogenesis of a branching structure	0,007140387	0,029306362
GO:0071897	DNA biosynthetic process	0,007140387	0,029306362
GO:0002209	behavioral defense response	0,007289259	0,029725106
GO:0046326	positive regulation of glucose import	0,007289259	0,029725106
GO:0046825	regulation of protein export from nucleus	0,007289259	0,029725106
GO:0060045	positive regulation of cardiac muscle cell proliferation	0,007289259	0,029725106
GO:1902742	apoptotic process involved in development	0,007289259	0,029725106
GO:0008654	phospholipid biosynthetic process	0,007377155	0,029826953
GO:0060420	regulation of heart growth	0,007421229	0,029826953
GO:0002320	lymphoid progenitor cell differentiation	0,00742705	0,029826953
GO:0003323	type B pancreatic cell development	0,00742705	0,029826953
GO:0008356	asymmetric cell division	0,00742705	0,029826953
GO:0010002	cardioblast differentiation	0,00742705	0,029826953
GO:0032462	regulation of protein homooligomerization	0,00742705	0,029826953
GO:0033194	response to hydroperoxide	0,00742705	0,029826953
GO:0045655	regulation of monocyte differentiation	0,00742705	0,029826953
GO:0051797	regulation of hair follicle development	0,00742705	0,029826953
GO:1900151	regulation of nuclear-transcribed mRNA catabolic process, deadenylation-dependent decay	0,00742705	0,029826953
GO:1902993	positive regulation of amyloid precursor protein catabolic process	0,00742705	0,029826953
GO:0010639	negative regulation of organelle organization	0,007480343	0,030003
GO:0010506	regulation of autophagy	0,007566244	0,030309224
GO:0110053	regulation of actin filament organization	0,007595219	0,030386924
GO:0040013	negative regulation of locomotion	0,007654559	0,030585762
GO:0019058	viral life cycle	0,00776185	0,030975459
GO:0009798	axis specification	0,007851335	0,031214779
GO:0045682	regulation of epidermis development	0,007851335	0,031214779
GO:0048864	stem cell development	0,007851335	0,031214779
GO:0017038	protein import	0,007909846	0,031408044
GO:0045862	positive regulation of proteolysis	0,007943476	0,031502152
GO:0060969	negative regulation of gene silencing	0,007994126	0,03162396
GO:1904591	positive regulation of protein import	0,007994126	0,03162396
GO:0018394	peptidyl-lysine acetylation	0,008010401	0,03164888
GO:0007405	neuroblast proliferation	0,008050701	0,031689712
GO:0030521	androgen receptor signaling pathway	0,008050701	0,031689712
GO:0034113	heterotypic cell-cell adhesion	0,008050701	0,031689712
GO:0034620	cellular response to unfolded protein	0,008101715	0,031701868
GO:0014902	myotube differentiation	0,008103751	0,031701868
GO:0042303	molting cycle	0,008103751	0,031701868
GO:0042633	hair cycle	0,008103751	0,031701868
GO:0060419	heart growth	0,008103751	0,031701868
GO:0032496	response to lipopolysaccharide	0,008165108	0,031902563
GO:0031330	negative regulation of cellular catabolic process	0,008279909	0,032311316
GO:0001942	hair follicle development	0,008299103	0,032346431
GO:0008286	insulin receptor signaling pathway	0,008441104	0,032859526
GO:0043244	regulation of protein complex disassembly	0,008492041	0,032904264
GO:0006123	mitochondrial electron transport, cytochrome c to oxygen	0,008535567	0,032904264
GO:0019646	aerobic electron transport chain	0,008535567	0,032904264
GO:0032891	negative regulation of organic acid transport	0,008535567	0,032904264
GO:0051412	response to corticosterone	0,008535567	0,032904264
GO:0055093	response to hyperoxia	0,008535567	0,032904264
GO:0070920	regulation of production of small RNA involved in gene silencing by RNA	0,008535567	0,032904264
GO:0072010	glomerular epithelium development	0,008535567	0,032904264
GO:0010676	positive regulation of cellular carbohydrate metabolic process	0,008613961	0,033085865
GO:0032835	glomerulus development	0,008613961	0,033085865
GO:0045453	bone resorption	0,008613961	0,033085865
GO:0001709	cell fate determination	0,00874228	0,033457218
GO:0035315	hair cell differentiation	0,00874228	0,033457218
GO:0072210	metanephric nephron development	0,00874228	0,033457218
GO:0001892	embryonic placenta development	0,008764917	0,033463119
GO:0006112	energy reserve metabolic process	0,008764917	0,033463119
GO:0002688	regulation of leukocyte chemotaxis	0,00889384	0,033914518
GO:0072331	signal transduction by p53 class mediator	0,009011985	0,034323781
GO:0046777	protein autophosphorylation	0,009132698	0,03474183
GO:0006606	protein import into nucleus	0,009151697	0,034772412
GO:0042982	amyloid precursor protein metabolic process	0,0092039	0,034887197
GO:0045815	positive regulation of gene expression, epigenetic	0,0092039	0,034887197
GO:0022404	molting cycle process	0,009249161	0,034933549
GO:0022405	hair cycle process	0,009249161	0,034933549
GO:0098773	skin epidermis development	0,009249161	0,034933549
GO:0032963	collagen metabolic process	0,009309414	0,03511931
GO:0070317	negative regulation of G0 to G1 transition	0,009534728	0,035883962
GO:0097178	ruffle assembly	0,009534728	0,035883962
GO:0022010	central nervous system myelination	0,009735936	0,036425163
GO:0032291	axon ensheathment in central nervous system	0,009735936	0,036425163
GO:0051000	positive regulation of nitric-oxide synthase activity	0,009735936	0,036425163
GO:0060065	uterus development	0,009735936	0,036425163
GO:0060575	intestinal epithelial cell differentiation	0,009735936	0,036425163
GO:0043297	apical junction assembly	0,009821105	0,036657352
GO:0045669	positive regulation of osteoblast differentiation	0,009821105	0,036657352
GO:0006109	regulation of carbohydrate metabolic process	0,009953339	0,037107259
GO:0050808	synapse organization	0,010010249	0,037275626
GO:0010906	regulation of glucose metabolic process	0,010182937	0,037874218
GO:0014033	neural crest cell differentiation	0,010274457	0,037973372
GO:0034333	adherens junction assembly	0,010274457	0,037973372
GO:0045778	positive regulation of ossification	0,010274457	0,037973372
GO:0046849	bone remodeling	0,010274457	0,037973372
GO:0000956	nuclear-transcribed mRNA catabolic process	0,010275339	0,037973372
GO:0051402	neuron apoptotic process	0,01028141	0,037973372
GO:0006986	response to unfolded protein	0,010298279	0,037991447
GO:0010470	regulation of gastrulation	0,010372435	0,038131998
GO:0017145	stem cell division	0,010372435	0,038131998
GO:0051154	negative regulation of striated muscle cell differentiation	0,010372435	0,038131998
GO:0060675	ureteric bud morphogenesis	0,010466154	0,038432002
GO:0050792	regulation of viral process	0,010605211	0,038897606
GO:0030510	regulation of BMP signaling pathway	0,010816258	0,039625869
GO:0035303	regulation of dephosphorylation	0,010943074	0,040044223
GO:0006541	glutamine metabolic process	0,011029577	0,040146393
GO:0048714	positive regulation of oligodendrocyte differentiation	0,011029577	0,040146393
GO:0060055	angiogenesis involved in wound healing	0,011029577	0,040146393
GO:1902254	negative regulation of intrinsic apoptotic signaling pathway by p53 class mediator	0,011029577	0,040146393
GO:0010950	positive regulation of endopeptidase activity	0,011034264	0,040146393
GO:0045428	regulation of nitric oxide biosynthetic process	0,011139609	0,040424356
GO:0072171	mesonephric tubule morphogenesis	0,011139609	0,040424356
GO:0002237	response to molecule of bacterial origin	0,011207353	0,040424356
GO:0021795	cerebral cortex cell migration	0,011256323	0,040424356
GO:0032881	regulation of polysaccharide metabolic process	0,011256323	0,040424356
GO:0032924	activin receptor signaling pathway	0,011256323	0,040424356
GO:0045429	positive regulation of nitric oxide biosynthetic process	0,011256323	0,040424356
GO:0045687	positive regulation of glial cell differentiation	0,011256323	0,040424356
GO:0045746	negative regulation of Notch signaling pathway	0,011256323	0,040424356
GO:0045773	positive regulation of axon extension	0,011256323	0,040424356
GO:0071364	cellular response to epidermal growth factor stimulus	0,011256323	0,040424356
GO:0007163	establishment or maintenance of cell polarity	0,011289045	0,040424356
GO:0030178	negative regulation of Wnt signaling pathway	0,011289045	0,040424356
GO:0043523	regulation of neuron apoptotic process	0,011289045	0,040424356
GO:2001056	positive regulation of cysteine-type endopeptidase activity	0,011551958	0,041319173
GO:0022612	gland morphogenesis	0,011603092	0,041455335
GO:0022618	ribonucleoprotein complex assembly	0,011821923	0,042189658
GO:2000573	positive regulation of DNA biosynthetic process	0,011842024	0,04221391
GO:0007589	body fluid secretion	0,011960011	0,042491274
GO:0036473	cell death in response to oxidative stress	0,011960011	0,042491274
GO:0048704	embryonic skeletal system morphogenesis	0,011960011	0,042491274
GO:0016331	morphogenesis of embryonic epithelium	0,011993182	0,042561462
GO:0010811	positive regulation of cell-substrate adhesion	0,012106813	0,042819181
GO:0010828	positive regulation of glucose transmembrane transport	0,012187271	0,042819181
GO:0014002	astrocyte development	0,012187271	0,042819181
GO:0014003	oligodendrocyte development	0,012187271	0,042819181
GO:0035307	positive regulation of protein dephosphorylation	0,012187271	0,042819181
GO:0045601	regulation of endothelial cell differentiation	0,012187271	0,042819181
GO:0051489	regulation of filopodium assembly	0,012187271	0,042819181
GO:1903573	negative regulation of response to endoplasmic reticulum stress	0,012187271	0,042819181
GO:1904407	positive regulation of nitric oxide metabolic process	0,012187271	0,042819181
GO:0051147	regulation of muscle cell differentiation	0,012211288	0,042856104
GO:0032528	microvillus organization	0,012417701	0,043293176
GO:0050996	positive regulation of lipid catabolic process	0,012417701	0,043293176
GO:0090023	positive regulation of neutrophil chemotaxis	0,012417701	0,043293176
GO:0099563	modification of synaptic structure	0,012417701	0,043293176
GO:1905564	positive regulation of vascular endothelial cell proliferation	0,012417701	0,043293176
GO:2000209	regulation of anoikis	0,012417701	0,043293176
GO:0055088	lipid homeostasis	0,012446682	0,043346584
GO:1901214	regulation of neuron death	0,012467405	0,043371144
GO:1901216	positive regulation of neuron death	0,012562686	0,043627834
GO:0071230	cellular response to amino acid stimulus	0,012573939	0,043627834
GO:0070997	neuron death	0,012589137	0,043627834
GO:0006338	chromatin remodeling	0,012623701	0,043627834
GO:0010821	regulation of mitochondrion organization	0,012623701	0,043627834
GO:0061138	morphogenesis of a branching epithelium	0,012623701	0,043627834
GO:1903050	regulation of proteolysis involved in cellular protein catabolic process	0,012756378	0,044038398
GO:0097237	cellular response to toxic substance	0,012908937	0,044384704
GO:0001678	cellular glucose homeostasis	0,01291265	0,044384704
GO:0016202	regulation of striated muscle tissue development	0,01291265	0,044384704
GO:0030902	hindbrain development	0,01291265	0,044384704
GO:0038061	NIK/NF-kappaB signaling	0,013046395	0,044747466
GO:0050770	regulation of axonogenesis	0,013046395	0,044747466
GO:0032388	positive regulation of intracellular transport	0,013144665	0,044867062
GO:0002062	chondrocyte differentiation	0,013161291	0,044867062
GO:0001974	blood vessel remodeling	0,013166116	0,044867062
GO:0042771	intrinsic apoptotic signaling pathway in response to DNA damage by p53 class mediator	0,013166116	0,044867062
GO:0048538	thymus development	0,013166116	0,044867062
GO:0048701	embryonic cranial skeleton morphogenesis	0,013166116	0,044867062
GO:0050810	regulation of steroid biosynthetic process	0,01318637	0,04488787
GO:0008088	axo-dendritic transport	0,013335879	0,045299605
GO:0071300	cellular response to retinoic acid	0,013335879	0,045299605
GO:0030168	platelet activation	0,013391274	0,045342133
GO:0051017	actin filament bundle assembly	0,013391274	0,045342133
GO:0090316	positive regulation of intracellular protein transport	0,013391274	0,045342133
GO:0043409	negative regulation of MAPK cascade	0,013479519	0,045592267
GO:0000075	cell cycle checkpoint	0,013541768	0,045754038
GO:0062012	regulation of small molecule metabolic process	0,013649314	0,046068346
GO:0070371	ERK1 and ERK2 cascade	0,013729939	0,046193884
GO:0006914	autophagy	0,013730189	0,046193884
GO:0061919	process utilizing autophagic mechanism	0,013730189	0,046193884
GO:0051258	protein polymerization	0,013805277	0,04627887
GO:0001945	lymph vessel development	0,013901319	0,04627887
GO:0003309	type B pancreatic cell differentiation	0,013901319	0,04627887
GO:0008334	histone mRNA metabolic process	0,013901319	0,04627887
GO:0032461	positive regulation of protein oligomerization	0,013901319	0,04627887
GO:0034110	regulation of homotypic cell-cell adhesion	0,013901319	0,04627887
GO:0060561	apoptotic process involved in morphogenesis	0,013901319	0,04627887
GO:0060571	morphogenesis of an epithelial fold	0,013901319	0,04627887
GO:1900101	regulation of endoplasmic reticulum unfolded protein response	0,013901319	0,04627887
GO:2000679	positive regulation of transcription regulatory region DNA binding	0,013901319	0,04627887
GO:1903708	positive regulation of hemopoiesis	0,013923217	0,046303186
GO:0060348	bone development	0,013947807	0,046336391
GO:0033077	T cell differentiation in thymus	0,014128358	0,046887109
GO:0072593	reactive oxygen species metabolic process	0,014159124	0,046907521
GO:0005978	glycogen biosynthetic process	0,014193649	0,046907521
GO:0009250	glucan biosynthetic process	0,014193649	0,046907521
GO:0022602	ovulation cycle process	0,014193649	0,046907521
GO:0032271	regulation of protein polymerization	0,014362898	0,047350017
GO:0010977	negative regulation of neuron projection development	0,014387241	0,047350017
GO:1901861	regulation of muscle tissue development	0,014387241	0,047350017
GO:1903707	negative regulation of hemopoiesis	0,014387241	0,047350017
GO:0014020	primary neural tube formation	0,014498158	0,047490829
GO:0051591	response to cAMP	0,014498158	0,047490829
GO:0060996	dendritic spine development	0,014498158	0,047490829
GO:0002446	neutrophil mediated immunity	0,014516058	0,047490829
GO:0034976	response to endoplasmic reticulum stress	0,01451984	0,047490829
GO:0051348	negative regulation of transferase activity	0,01451984	0,047490829
GO:0045667	regulation of osteoblast differentiation	0,01486437	0,048517664
GO:0048706	embryonic skeletal system development	0,01486437	0,048517664
GO:0016573	histone acetylation	0,014904958	0,048550246
GO:0048634	regulation of muscle organ development	0,014904958	0,048550246
GO:0007568	aging	0,015088537	0,049097814
GO:0045646	regulation of erythrocyte differentiation	0,01527062	0,049363129
GO:0050435	amyloid-beta metabolic process	0,01527062	0,049363129
GO:0061614	pri-miRNA transcription by RNA polymerase II	0,01527062	0,049363129
GO:0120163	negative regulation of cold-induced thermogenesis	0,01527062	0,049363129
GO:0031345	negative regulation of cell projection organization	0,015319223	0,049363129
GO:0030509	BMP signaling pathway	0,015436076	0,049363129
GO:0043271	negative regulation of ion transport	0,015436076	0,049363129
GO:0061572	actin filament bundle organization	0,015436076	0,049363129
GO:0018209	peptidyl-serine modification	0,015443715	0,049363129
GO:0071426	ribonucleoprotein complex export from nucleus	0,015465414	0,049363129
GO:0010996	response to auditory stimulus	0,015481253	0,049363129
GO:0022011	myelination in peripheral nervous system	0,015481253	0,049363129
GO:0032292	peripheral nervous system axon ensheathment	0,015481253	0,049363129
GO:0034114	regulation of heterotypic cell-cell adhesion	0,015481253	0,049363129
GO:0045992	negative regulation of embryonic development	0,015481253	0,049363129
GO:0060074	synapse maturation	0,015481253	0,049363129
GO:0060740	prostate gland epithelium morphogenesis	0,015481253	0,049363129
GO:0060914	heart formation	0,015481253	0,049363129
GO:0071624	positive regulation of granulocyte chemotaxis	0,015481253	0,049363129
GO:1903203	regulation of oxidative stress-induced neuron death	0,015481253	0,049363129
GO:0051346	negative regulation of hydrolase activity	0,015612601	0,049731958
GO:0071824	protein-DNA complex subunit organization	0,01564402	0,049782057
GO:0006644	phospholipid metabolic process	0,015792612	0,050149521
GO:0006305	DNA alkylation	0,015806916	0,050149521
GO:0006306	DNA methylation	0,015806916	0,050149521
GO:0070301	cellular response to hydrogen peroxide	0,015898111	0,05038846
GO:0043467	regulation of generation of precursor metabolites and energy	0,01598078	0,050599926
GO:0022409	positive regulation of cell-cell adhesion	0,016017847	0,050666727
GO:0010595	positive regulation of endothelial cell migration	0,016083539	0,050773276
GO:0071166	ribonucleoprotein complex localization	0,016083539	0,050773276
GO:0060759	regulation of response to cytokine stimulus	0,016305432	0,051422594
GO:0044764	multi-organism cellular process	0,016397732	0,051457927
GO:0046850	regulation of bone remodeling	0,016397732	0,051457927
GO:0055010	ventricular cardiac muscle tissue morphogenesis	0,016397732	0,051457927
GO:0055023	positive regulation of cardiac muscle tissue growth	0,016397732	0,051457927
GO:0090199	regulation of release of cytochrome c from mitochondria	0,016397732	0,051457927
GO:0034446	substrate adhesion-dependent cell spreading	0,016631979	0,051612173
GO:0035282	segmentation	0,016631979	0,051612173
GO:0055024	regulation of cardiac muscle tissue development	0,016631979	0,051612173
GO:0061640	cytoskeleton-dependent cytokinesis	0,016631979	0,051612173
GO:0033555	multicellular organismal response to stress	0,01669395	0,051612173
GO:1903524	positive regulation of blood circulation	0,01669395	0,051612173
GO:0003211	cardiac ventricle formation	0,016772244	0,051612173
GO:0007220	Notch receptor processing	0,016772244	0,051612173
GO:0032000	positive regulation of fatty acid beta-oxidation	0,016772244	0,051612173
GO:0032070	regulation of deoxyribonuclease activity	0,016772244	0,051612173
GO:0032463	negative regulation of protein homooligomerization	0,016772244	0,051612173
GO:0032464	positive regulation of protein homooligomerization	0,016772244	0,051612173
GO:0044803	multi-organism membrane organization	0,016772244	0,051612173
GO:0045792	negative regulation of cell size	0,016772244	0,051612173
GO:0060768	regulation of epithelial cell proliferation involved in prostate gland development	0,016772244	0,051612173
GO:0072015	glomerular visceral epithelial cell development	0,016772244	0,051612173
GO:0072203	cell proliferation involved in metanephros development	0,016772244	0,051612173
GO:0072610	interleukin-12 secretion	0,016772244	0,051612173
GO:1903799	negative regulation of production of miRNAs involved in gene silencing by miRNA	0,016772244	0,051612173
GO:2001225	regulation of chloride transport	0,016772244	0,051612173
GO:0019080	viral gene expression	0,016815629	0,051695537
GO:0071826	ribonucleoprotein complex subunit organization	0,016832938	0,051698656
GO:0097191	extrinsic apoptotic signaling pathway	0,017050079	0,052314911
GO:0003148	outflow tract septum morphogenesis	0,017158147	0,052342768
GO:0010971	positive regulation of G2/M transition of mitotic cell cycle	0,017158147	0,052342768
GO:0035883	enteroendocrine cell differentiation	0,017158147	0,052342768
GO:0036475	neuron death in response to oxidative stress	0,017158147	0,052342768
GO:1902175	regulation of oxidative stress-induced intrinsic apoptotic signaling pathway	0,017158147	0,052342768
GO:2000191	regulation of fatty acid transport	0,017158147	0,052342768
GO:0043406	positive regulation of MAP kinase activity	0,017317872	0,052779277
GO:0048839	inner ear development	0,017337409	0,05278811
GO:0005977	glycogen metabolic process	0,017613431	0,053321497
GO:0060998	regulation of dendritic spine development	0,017613431	0,053321497
GO:0072078	nephron tubule morphogenesis	0,017613431	0,053321497
GO:1900006	positive regulation of dendrite development	0,017613431	0,053321497
GO:1900182	positive regulation of protein localization to nucleus	0,017613431	0,053321497
GO:1903036	positive regulation of response to wounding	0,017613431	0,053321497
GO:0018393	internal peptidyl-lysine acetylation	0,017698213	0,053527082
GO:0046887	positive regulation of hormone secretion	0,018042762	0,054517178
GO:0060249	anatomical structure homeostasis	0,018092659	0,05461593
GO:0021549	cerebellum development	0,018169105	0,054794558
GO:0016236	macroautophagy	0,018522623	0,055620819
GO:0001894	tissue homeostasis	0,018525279	0,055620819
GO:0006457	protein folding	0,018525279	0,055620819
GO:0006073	cellular glucan metabolic process	0,018565801	0,055620819
GO:0006695	cholesterol biosynthetic process	0,018565801	0,055620819
GO:0043242	negative regulation of protein complex disassembly	0,018565801	0,055620819
GO:0044042	glucan metabolic process	0,018565801	0,055620819
GO:0001961	positive regulation of cytokine-mediated signaling pathway	0,018804977	0,055823195
GO:0014009	glial cell proliferation	0,018804977	0,055823195
GO:0045540	regulation of cholesterol biosynthetic process	0,018804977	0,055823195
GO:0106118	regulation of sterol biosynthetic process	0,018804977	0,055823195
GO:1903793	positive regulation of anion transport	0,018804977	0,055823195
GO:0001958	endochondral ossification	0,018932472	0,055823195
GO:0003180	aortic valve morphogenesis	0,018932472	0,055823195
GO:0008045	motor neuron axon guidance	0,018932472	0,055823195
GO:0032770	positive regulation of monooxygenase activity	0,018932472	0,055823195
GO:0035116	embryonic hindlimb morphogenesis	0,018932472	0,055823195
GO:0036075	replacement ossification	0,018932472	0,055823195
GO:0036296	response to increased oxygen levels	0,018932472	0,055823195
GO:0045577	regulation of B cell differentiation	0,018932472	0,055823195
GO:0051123	RNA polymerase II preinitiation complex assembly	0,018932472	0,055823195
GO:0060512	prostate gland morphogenesis	0,018932472	0,055823195
GO:1902003	regulation of amyloid-beta formation	0,018932472	0,055823195
GO:1903792	negative regulation of anion transport	0,018932472	0,055823195
GO:0048259	regulation of receptor-mediated endocytosis	0,018972986	0,05589071
GO:0034504	protein localization to nucleus	0,01917177	0,056423899
GO:0030198	extracellular matrix organization	0,019479649	0,057276877
GO:1902653	secondary alcohol biosynthetic process	0,019551483	0,057434864
GO:0043900	regulation of multi-organism process	0,019781596	0,057861794
GO:0050999	regulation of nitric-oxide synthase activity	0,020086296	0,057861794
GO:0010952	positive regulation of peptidase activity	0,020124968	0,057861794
GO:0046683	response to organophosphorus	0,020164109	0,057861794
GO:0051251	positive regulation of lymphocyte activation	0,02022077	0,057861794
GO:0003264	regulation of cardioblast proliferation	0,020225711	0,057861794
GO:0009950	dorsal/ventral axis specification	0,020225711	0,057861794
GO:0033327	Leydig cell differentiation	0,020225711	0,057861794
GO:0034115	negative regulation of heterotypic cell-cell adhesion	0,020225711	0,057861794
GO:0039532	negative regulation of viral-induced cytoplasmic pattern recognition receptor signaling pathway	0,020225711	0,057861794
GO:0045793	positive regulation of cell size	0,020225711	0,057861794
GO:0048096	chromatin-mediated maintenance of transcription	0,020225711	0,057861794
GO:0048853	forebrain morphogenesis	0,020225711	0,057861794
GO:0060379	cardiac muscle cell myoblast differentiation	0,020225711	0,057861794
GO:0060439	trachea morphogenesis	0,020225711	0,057861794
GO:0060525	prostate glandular acinus development	0,020225711	0,057861794
GO:0060736	prostate gland growth	0,020225711	0,057861794
GO:0060767	epithelial cell proliferation involved in prostate gland development	0,020225711	0,057861794
GO:0060837	blood vessel endothelial cell differentiation	0,020225711	0,057861794
GO:0061307	cardiac neural crest cell differentiation involved in heart development	0,020225711	0,057861794
GO:0061308	cardiac neural crest cell development involved in heart development	0,020225711	0,057861794
GO:0070106	interleukin-27-mediated signaling pathway	0,020225711	0,057861794
GO:0070278	extracellular matrix constituent secretion	0,020225711	0,057861794
GO:0070587	regulation of cell-cell adhesion involved in gastrulation	0,020225711	0,057861794
GO:0070757	interleukin-35-mediated signaling pathway	0,020225711	0,057861794
GO:0072310	glomerular epithelial cell development	0,020225711	0,057861794
GO:1904321	response to forskolin	0,020225711	0,057861794
GO:1904322	cellular response to forskolin	0,020225711	0,057861794
GO:1905383	protein localization to presynapse	0,020225711	0,057861794
GO:0018105	peptidyl-serine phosphorylation	0,02033637	0,058125955
GO:0000288	nuclear-transcribed mRNA catabolic process, deadenylation-dependent decay	0,020570886	0,058585137
GO:0006278	RNA-dependent DNA biosynthetic process	0,020570886	0,058585137
GO:0006809	nitric oxide biosynthetic process	0,020570886	0,058585137
GO:1901224	positive regulation of NIK/NF-kappaB signaling	0,020570886	0,058585137
GO:0055017	cardiac muscle tissue growth	0,020652893	0,058765937
GO:0046890	regulation of lipid biosynthetic process	0,020719177	0,058775721
GO:0014044	Schwann cell development	0,020804538	0,058775721
GO:0021799	cerebral cortex radially oriented cell migration	0,020804538	0,058775721
GO:0030511	positive regulation of transforming growth factor beta receptor signaling pathway	0,020804538	0,058775721
GO:0070229	negative regulation of lymphocyte apoptotic process	0,020804538	0,058775721
GO:1902624	positive regulation of neutrophil migration	0,020804538	0,058775721
GO:1903846	positive regulation of cellular response to transforming growth factor beta stimulus	0,020804538	0,058775721
GO:2000171	negative regulation of dendrite development	0,020804538	0,058775721
GO:0006475	internal protein amino acid acetylation	0,020848971	0,058848846
GO:0030010	establishment of cell polarity	0,020908298	0,058963846
GO:0031529	ruffle organization	0,021420132	0,060300079
GO:1900024	regulation of substrate adhesion-dependent cell spreading	0,021420132	0,060300079
GO:0035051	cardiocyte differentiation	0,021523932	0,060446045
GO:0021510	spinal cord development	0,0215295	0,060446045
GO:0042136	neurotransmitter biosynthetic process	0,0215295	0,060446045
GO:0043405	regulation of MAP kinase activity	0,021571773	0,060446045
GO:0055021	regulation of cardiac muscle tissue growth	0,021624402	0,060446045
GO:0061333	renal tubule morphogenesis	0,021624402	0,060446045
GO:0072332	intrinsic apoptotic signaling pathway by p53 class mediator	0,021624402	0,060446045
GO:1903313	positive regulation of mRNA metabolic process	0,021624402	0,060446045
GO:0048738	cardiac muscle tissue development	0,021754227	0,060755412
GO:0051056	regulation of small GTPase mediated signal transduction	0,022036762	0,061490348
GO:0043312	neutrophil degranulation	0,02205778	0,061494913
GO:0007265	Ras protein signal transduction	0,022216862	0,061884037
GO:0021675	nerve development	0,022712407	0,06281086
GO:0030193	regulation of blood coagulation	0,022712407	0,06281086
GO:1900034	regulation of cellular response to heat	0,022712407	0,06281086
GO:0021772	olfactory bulb development	0,022774503	0,06281086
GO:0050690	regulation of defense response to virus by virus	0,022774503	0,06281086
GO:0070168	negative regulation of biomineral tissue development	0,022774503	0,06281086
GO:0090022	regulation of neutrophil chemotaxis	0,022774503	0,06281086
GO:1902751	positive regulation of cell cycle G2/M phase transition	0,022774503	0,06281086
GO:0060326	cell chemotaxis	0,022785696	0,06281086
GO:0000186	activation of MAPKK activity	0,022806969	0,06281086
GO:0030195	negative regulation of blood coagulation	0,022806969	0,06281086
GO:0032964	collagen biosynthetic process	0,022806969	0,06281086
GO:0070228	regulation of lymphocyte apoptotic process	0,022806969	0,06281086
GO:0006469	negative regulation of protein kinase activity	0,022916325	0,063057291
GO:0002283	neutrophil activation involved in immune response	0,023237752	0,06388633
GO:1904659	glucose transmembrane transport	0,023357424	0,06415974
GO:0009199	ribonucleoside triphosphate metabolic process	0,023476622	0,064431377
GO:0071772	response to BMP	0,023641432	0,06444119
GO:0071773	cellular response to BMP stimulus	0,023641432	0,06444119
GO:0022617	extracellular matrix disassembly	0,023835261	0,06444119
GO:1900046	regulation of hemostasis	0,023835261	0,06444119
GO:0002551	mast cell chemotaxis	0,023947364	0,06444119
GO:0002903	negative regulation of B cell apoptotic process	0,023947364	0,06444119
GO:0003161	cardiac conduction system development	0,023947364	0,06444119
GO:0003207	cardiac chamber formation	0,023947364	0,06444119
GO:0033197	response to vitamin E	0,023947364	0,06444119
GO:0042118	endothelial cell activation	0,023947364	0,06444119
GO:0045472	response to ether	0,023947364	0,06444119
GO:0045579	positive regulation of B cell differentiation	0,023947364	0,06444119
GO:0045955	negative regulation of calcium ion-dependent exocytosis	0,023947364	0,06444119
GO:0060742	epithelial cell differentiation involved in prostate gland development	0,023947364	0,06444119
GO:0070586	cell-cell adhesion involved in gastrulation	0,023947364	0,06444119
GO:0072017	distal tubule development	0,023947364	0,06444119
GO:0090331	negative regulation of platelet aggregation	0,023947364	0,06444119
GO:0097531	mast cell migration	0,023947364	0,06444119
GO:0098974	postsynaptic actin cytoskeleton organization	0,023947364	0,06444119
GO:0099640	axo-dendritic protein transport	0,023947364	0,06444119
GO:1902337	regulation of apoptotic process involved in morphogenesis	0,023947364	0,06444119
GO:1903894	regulation of IRE1-mediated unfolded protein response	0,023947364	0,06444119
GO:1905244	regulation of modification of synaptic structure	0,023947364	0,06444119
GO:0035304	regulation of protein dephosphorylation	0,024076138	0,064732811
GO:1900047	negative regulation of hemostasis	0,024247248	0,065137666
GO:0000910	cytokinesis	0,024378676	0,065435328
GO:0006473	protein acetylation	0,024552575	0,065790773
GO:0034764	positive regulation of transmembrane transport	0,024552575	0,065790773
GO:0021988	olfactory lobe development	0,024842376	0,066231688
GO:0060292	long-term synaptic depression	0,024842376	0,066231688
GO:0097106	postsynaptic density organization	0,024842376	0,066231688
GO:1902230	negative regulation of intrinsic apoptotic signaling pathway in response to DNA damage	0,024842376	0,066231688
GO:1902253	regulation of intrinsic apoptotic signaling pathway by p53 class mediator	0,024842376	0,066231688
GO:1902895	positive regulation of pri-miRNA transcription by RNA polymerase II	0,024842376	0,066231688
GO:0016126	sterol biosynthetic process	0,024993306	0,066578129
GO:0032874	positive regulation of stress-activated MAPK cascade	0,025131888	0,066835056
GO:0051262	protein tetramerization	0,025131888	0,066835056
GO:0043414	macromolecule methylation	0,025446414	0,067614821
GO:0010823	negative regulation of mitochondrion organization	0,025741365	0,068170172
GO:0042306	regulation of protein import into nucleus	0,025741365	0,068170172
GO:0044380	protein localization to cytoskeleton	0,025741365	0,068170172
GO:0070830	bicellular tight junction assembly	0,025741365	0,068170172
GO:0016525	negative regulation of angiogenesis	0,025901222	0,068479196
GO:0070304	positive regulation of stress-activated protein kinase signaling cascade	0,025901222	0,068479196
GO:0016925	protein sumoylation	0,026186869	0,069119206
GO:0046209	nitric oxide metabolic process	0,026186869	0,069119206
GO:0006406	mRNA export from nucleus	0,026290054	0,069161405
GO:0022037	metencephalon development	0,026290054	0,069161405
GO:0035305	negative regulation of dephosphorylation	0,026290054	0,069161405
GO:0071427	mRNA-containing ribonucleoprotein complex export from nucleus	0,026290054	0,069161405
GO:1990778	protein localization to cell periphery	0,026572007	0,069845226
GO:0099003	vesicle-mediated transport in synapse	0,026647465	0,069898467
GO:0010675	regulation of cellular carbohydrate metabolic process	0,026658357	0,069898467
GO:0038127	ERBB signaling pathway	0,026658357	0,069898467
GO:0046488	phosphatidylinositol metabolic process	0,02668683	0,069915341
